# Heterogeneity of foam cell biogenesis across diseases

**DOI:** 10.1101/2023.06.08.542766

**Published:** 2023-06-08

**Authors:** Valentina Guerrini, Brendan Prideaux, Rehan Khan, Selvakumar Subbian, Yina Wang, Evita Sadimin, Siddhi Pawar, Rahul Ukey, Eric A. Singer, Chaoyang Xue, Maria Laura Gennaro

**Affiliations:** 1 Public Health Research Institute and Department of Medicine, Rutgers New Jersey Medical School, Rutgers Biomedical and Health Sciences, Newark, NJ 07103; 2 Department of Neurobiology, University of Texas Medical Branch, Galveston, TX 77555; 3 Public Health Research Institute and Department of Microbiology, Rutgers New Jersey Medical School, Rutgers Biomedical and Health Sciences, Newark, NJ 07103; 4 Section of Urologic Pathology, Rutgers Cancer Institute of New Jersey and Rutgers Robert Wood Johnson Medical School, New Brunswick, NJ 08901

**Keywords:** kidney disease, tuberculosis, cryptococcosis, papillary renal cell carcinoma, lipid metabolism, storage lipids

## Abstract

Foam cells are dysfunctional, lipid-laden macrophages associated with chronic inflammation of infectious and non-infectious origin. For decades, the paradigm of foam cell biology has been atherogenesis, in which macrophages accumulate cholesteryl esters. Our previous work showed that foam cells in tuberculous lung lesions are surprisingly triglyceride-rich, suggesting multiple modalities of foam cell biogenesis. In the present study, we used matrix-assisted laser desorption/ionization mass spectrometry imaging to assess the spatial distribution of storage lipids relative to foam-cell-rich areas in murine lungs infected with the fungal pathogen *Cryptococcus neoformans* and in human papillary renal cell carcinoma resection tissues. We also analyzed neutral lipid content and the transcriptional program of lipid-laden macrophages generated under corresponding in vitro conditions. The in vivo data were consistent with in vitro findings showing that *C. neoformans*-infected macrophages accumulated triglycerides, while macrophages exposed to human renal cell carcinoma-conditioned medium accumulated both triglycerides and cholesteryl esters. Moreover, macrophage transcriptome analyses provided evidence for condition-specific metabolic remodeling. The in vitro data also showed that although both *Mycobacterium tuberculosis* and *C. neoformans* infections induced triglyceride accumulation in macrophages, they did so by different molecular mechanisms, as evidenced by different sensitivity of lipid accumulation to the drug rapamycin and the characteristics of macrophage transcriptome remodeling. Collectively, these data demonstrate that the mechanisms of foam cell formation are specific to the disease microenvironment. Since foam cells have been regarded as targets of pharmacological intervention in several diseases, recognizing that their formation is disease-specific opens new research directions of biomedical significance.

Chronic inflammation of infectious and non-infectious origin is often associated with the presence of foam cells, lipid-laden macrophages that exhibit impaired immune function and can contribute to pathogenesis (1). Foam cells form when, due to dysregulated metabolism, lipids accumulate beyond the homeostatic capacity of macrophages. The lipids are stored as droplets that confer a foamy appearance to the macrophages (2). Our understanding of foam cell biology has been largely based on studies of atherogenesis, a disease in which uptake of normal and proinflammatory lipoproteins by macrophages in the arterial wall leads to imbalanced cholesterol metabolism and formation of cholesterol-laden foam cells (3). The accumulation of foam cells in the arterial intima leads to chronic inflammation, cell death, and tissue necrosis (3). A similar situation is observed in tuberculosis, a chronic inflammatory disease of the lung caused by *Mycobacterium tuberculosis*. In the tuberculous lung lesions, which are called granulomas, the presence of tissue necrosis is associated with foam cell accumulation (1). Indeed, foam cells are a hallmark of both the atherosclerotic plaque and the necrotizing tuberculous granuloma (3, 4). We were surprised to find that the foam cells of necrotic tuberculous lung lesions are enriched in triglycerides (TAG) (5). Work with cultured human macrophages and mice further established that *M. tuberculosis* infection induces TAG accumulation in macrophages (5, 6). Thus, the atherosclerosis and tuberculosis models of foam cells are fundamentally different, indicating that foam cells may form via different mechanisms in different diseases.

To test the hypothesis that foam cell biogenesis is disease-specific, we began a study of foam cells resulting from another infectious disease, cryptococcosis, and from a form of cancer. Cryptococcosis is a clinically heterogeneous disease caused by the fungal pathogen *Cryptococcus neoformans*. It affects the lung and other organ systems, including the central nervous system, particularly in immunocompromised individuals (7). Foam cells have been observed in human tissue biopsies from pulmonary and extrapulmonary cryptococcosis (8, 9) and in the lungs of infected mice (10). Foam cells have also been associated with several forms of cancer of many organ systems that include liver, lung, colon/rectum, and kidney (11–14). Indeed, the presence of foam cells is a histological feature of papillary renal cell carcinoma (pRCC) (15, 16). Factors released by cultured pRCC cells induce lipid accumulation in macrophages (13), indicating that the microenvironment of this tumor is lipogenic for macrophages. The nature of storage lipids and the mechanism of foam cell formation are poorly understood in these pathologies.

In the present work, we assessed the spatial distribution of foam cells and storage lipids in *C. neoformans*-infected murine lungs and in human pRCC specimens. We then analyzed lipid content and the transcriptional program of lipid-laden macrophages generated under in vitro conditions that corresponded to these two diseases. The data established that the mechanism underlying foam cell formation varies with disease context. We can no longer base our understanding of foam cell biogenesis only on work focused on atherogenesis. Expanding our view of foam cell biogenesis may provide new targets for therapeutic intervention into diseases -- such as atherosclerosis, tuberculosis, multiple sclerosis, and certain cancers -- in which foam cell appearance and poor clinical outcome are associated (reviewed in (1)).

## Materials and Methods.

The supplementary materials include the description of the materials and methods utilized to generate, culture, infect and/or treat human monocyte-derived macrophages; to perform cell culture processing and measurements of released cytokines and chemokines; neutral lipid content; RNA extraction and bulk RNA sequencing with the associated statistical analyses; to conduct mouse infections with *C. neoformans*; to obtain and process cryptococcus-infected murine lungs and human cancerous kidney specimens for histopathology and analysis of spatial distribution of cholesteryl esters and triglycerides by matrix-assisted laser desorption/ionization mass spectrometry.

## Results

### Foam cells cluster peri- or extra-lesionally and associate with TAG species in *C. neoformans*-infected murine lungs.

**Fig. 1.**
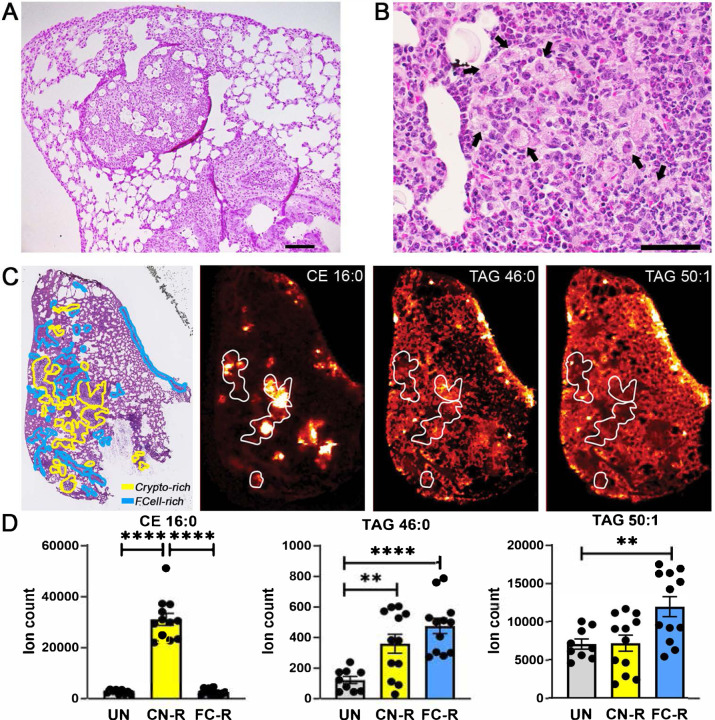
Spatial distribution of foam cells and storage lipids in *C. neoformans*-infected murine lungs. **A-B.** H&E staining of formalin-fixed, paraffin-embedded lung sections from *C. neoformans* H99-infected mice. Images were photographed at (A) 100x magnification; scale bar is 100 μm; and (B) 400x magnification; scale bar is 10 μm. Black arrows indicate foam cells. **C.** MALDI imaging of representative CE and TAG species in infected lung sections. The left panel shows H&E staining of infected tissue sections (scale bar is 2 mm). The yellow lines delineate areas enriched in fungal cells (Crypto-rich), while the blue lines define areas enriched in foam cells (FCell-rich). The three additional panels show MALDI imaging of storage lipids in lung sections contiguous to those used for H&E staining. Representative species are shown: CE (16:0) [M+K]^+^ *m/z* 663.48 and TAG (46:0) [M+K]^+^ *m/z* 817.669 signals tend to correspond to cryptococci-enriched areas, while TAG (50:1) [M+K]^+^ *m/z* 871.716 tends to be reduced in those same areas. Areas delimited by white lines correspond to some cryptococci-enriched areas in the H&E-stained section. Corresponding images of uninfected lung sections are shown in Fig. S2. **D.** Quantification of CE and TAG MALDI imaging intensity (expressed as ion count). Quantification of lipid species was performed in uninfected tissue and in areas A and B of the infected tissue. Mean and SEM of 9 sections from three uninfected animals (3 sections per animal) and 12 sections from four infected animals (3 sections per animal) are shown. **, *p* < 0.05; ****, *p* < 0.001 (unpaired *t*-test).

Foam cells form during pulmonary and extrapulmonary cryptococcal infection (8, 9). We used a model of pulmonary cryptococcosis in C57BL/6 mice to assess the spatial relationship between foam cells and neutral lipids (CE and TAG) in infected lungs. At 7 days post intranasal infection, infected mouse lungs exhibited several granulomatous nodular lesions visible at low magnification (Fig. S1). The lesions consisted of large aggregates of fungal cells surrounded by inflammatory infiltrates comprised mostly of polymorphonuclear cells, macrophages and lymphocyte aggregates, and epithelioid cells (Fig. 1A). Foam cells tended to form clusters in peri- or extra-lesional areas of the infected foci in the lungs [hematoxylin and eosin (H&E)-stained lung slices in Fig. 1BC]. When we used matrix-assisted laser desorption/ionization mass spectrometry (MALDI) imaging of sections adjacent to those used for H&E staining, we detected multiple TAG and CE species in the infected lungs (Table S1). All CE species localized in the fungus-rich lesions (e.g., compare H&E staining and MALDI imaging for CE 16:0 in Fig. 1C). In contrast, TAG species were distributed throughout the lung tissue, with some species, such as TAG 46:0, more prominently found within the lesions and others, such as TAG 50:1, found extra-lesionally (Fig. 1C, with corresponding ion counts in Fig. 1D) (see Fig. S2 for uninfected control tissue). Localization of some TAG species and CE species in the fungus-rich lesions is consistent with the presence of TAG and sterols in fungal cells (17, 18). In addition, the spatial distribution of some TAG species, such as TAG 50:1 in Fig. 1C, which was present throughout the tissue but approximately two-fold lower in the fungus-rich lesions, corresponds to that of foam cells (compare H&E staining and MALDI imaging in Fig. 1C), suggesting that *Cryptococcus*-induced foam cells are TAG enriched.

### *Cryptococcus neoformans* infection induces accumulation of TAG-rich lipid droplets in macrophages via an mTORC1-independent pathway.

**Fig. 2.**
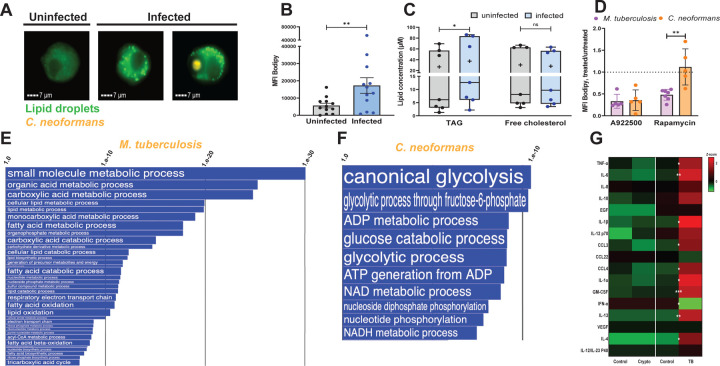
Characterization of lipid droplets induced by *C. neoformans* infection and comparisons with *M. tuberculosis*-induced effects. **A.** Lipid droplet content of MDM determined by imaging flow cytometry. Representative images of MDM uninfected and infected with mCherry-tagged *C. neoformans* (orange fluorescence) and stained with Bodipy 493/503 (neutral lipid dye, green fluorescence) acquired by imaging flow cytometry at 24 hrs post-infection. **B.** Quantification of lipid droplet content by imaging flow cytometry expressed as median fluorescence intensity of Bodipy 493/503. Each dot represents one human donor. **C.** Neutral lipid measurements in MDM. TAG and free cholesterol were measured in infected and uninfected cells using a commercially available kit. The box plots show lower quartile, median, and upper quartile of the distribution of multiple donors. The whiskers represent minimum and maximum values. The plus symbol indicates the mean. ns, non-significant; *, *p* < 0.05 (paired *t*-test). Each dot represents one human donor. **D.** Effect on lipid droplets of MDM treatment with A922500 (DGAT-1 inhibitor) or rapamycin (mTORC1 inhibitor). MDM were infected with *C. neoformans* or with *M. tuberculosis* for 24h with an MOI = 4 in both infections. DMSO (vehicle control) and each drug were added for the duration of infection. Lipid droplet content was quantified by imaging flow cytometry and expressed as Bodipy MFI, as described in panel A and in Fig. S3AB. Results are shown as ratios of Bodipy MFI of drug-treated to vehicle-treated infected cells. Mean and SD are shown. ns, non-significant; **, *p* < 0.01 (unpaired *t*-test). Each dot represents one human donor. **E-F.** Pathway analysis of the transcriptomes of *M. tuberculosis*- and *C. neoformans*-infected MDM. The two panels show Gene Ontology (GO) annotations related to metabolic processes that were (**E**) downregulated in *M. tuberculosis*-infected MDM and (**F**) upregulated in *C. neoformans*-infected MDM, relative to uninfected control cells. The differential expression between sample classes (infected vs uninfected) was tested with coincident extreme ranks in numerical observations (CERNO). Pathways were selected using a cutoff false discovery rate of 0.05; the *p*-values for these pathways are plotted onto the x-axis. To represent effect size, pathway gene sets containing fewer genes were given greater bar height/font size than were larger sets that yielded similar *p* values. **G.** Cytokine/chemokine levels in MDM. MDM from five donors were infected with *C. neoformans* (Crypto) or *M. tuberculosis* (TB), as in panel C. A Luminex platform was used to measure cytokine/chemokine/growth factor concentration in the supernatants of uninfected (control) and infected cells. Each sample was measured in duplicate. Average Z-score of 5 donors was calculated for each analyte (gradient from green to red represents increasing concentrations). White asterisks indicate significant differences between uninfected and infected cells. *, *p* < 0.05; **, *p* < 0.01; ***, *p* < 0.001 (paired *t*-test).

MALDI imaging provides information about the spatial distribution of analytes in tissues, but it does not have the single-cell resolution needed to precisely assign a particular neutral lipid to a specific cell type. Thus, we utilized an in vitro infection model to study neutral lipid accumulation in macrophages infected with *C. neoformans*. We infected primary human monocyte-derived macrophages (MDM) with mCherry-expressing *C. neoformans* H99, quantified lipid droplet content by imaging flow cytometry, and observed a significant lipid droplet accumulation in infected macrophages (3.5-fold increase relative to uninfected cells) (Fig. 2AB). Lipid-droplet-enriched macrophages in the infected culture wells included both those containing fungal cells and those that did not (Fig. 2A and quantitative data in Fig. S3A). These data indicated that lipid droplet formation does not require internalization of fungal cells. Since lipid droplets accumulated in macrophages exposed to cell-free *C. neoformans* culture filtrate (Fig. S3B) but not in macrophages exposed to heat-killed fungi (Fig. S3C), lipid droplet accumulation in macrophages appears to involve a factor(s) released from live *C. neoformans* cells.

When we measured storage lipid content in *C. neoformans*-infected cells by an enzymatic assay, we found that infection increased the content of intracellular TAG but not cholesterol derivatives (Fig. 2C). Moreover, lipid droplet accumulation in *C. neoformans*-infected cells was essentially abrogated by treatment with an inhibitor of diglyceride acetyl transferase (A9222500), the enzyme that catalyzes the conversion of di- to tri-glycerides (Fig. 2D). This finding supports the conclusion that *C. neoformans*-induced lipid droplets are TAG enriched, as previously seen with *M. tuberculosis* infection ((5) and Fig. 2D).

Our previous work showed that the accumulation of TAG-rich lipid droplets in macrophages infected with *M. tuberculosis* requires mTORC1 signaling, as it is inhibited by rapamycin treatment (5). Unlike the *M. tuberculosis* case, however, rapamycin had no effect on lipid droplet accumulation in *C. neoformans*-infected macrophages (Fig. 2D). Thus, even though *M. tuberculosis* and *C. neoformans* both induce accumulation of TAG-rich lipid droplets, the two pathogens do so by utilizing different signaling mechanisms.

### Transcriptomics identify different molecular mechanisms of TAG accumulation in *M. tuberculosis*- and *C. neoformans*-infected macrophages.

We investigated the pathways underlying TAG accumulation in *M. tuberculosis*- and *C. neoformans*- infected macrophages by conducting transcriptomics analyses of macrophages obtained from the same donors and infected in vitro with either pathogen. When we analyzed the Gene Ontology (GO) annotations related to metabolic processes, we found that the most informative signatures of macrophage metabolic reprogramming associated with TAG accumulation derived from the downregulated pathways in *M. tuberculosis*-infected macrophages and from the upregulated pathways in *C. neoformans*-infected macrophages. The former included lipid catabolism, fatty acid oxidation, oxidative phosphorylation, and electron transport chain (Fig. 2E), while the latter were enriched for glycolysis (Fig. 2F).

Metabolism-related analyses at the gene level (Table S2) showed that, in *M. tuberculosis*-infected macrophages, the top three downregulated metabolic genes encoded: (i) acyl-CoA synthase (ACSM5), (ii) carnitine octanoyl transferase (CROT), which converts acyl-CoA to acyl-carnitine, a step required for transport across the mitochondrial membrane, and iii) aldehyde hydrogenase (ALDH3A2), which oxidizes fatty aldehydes to fatty acids. Downregulation of these genes likely leads to defective fatty acid oxidation. In *C. neoformans* infection, the top five upregulated metabolic genes all encoded glycolytic enzymes (Table S3). We also found indicators of reduced mitochondrial functions in *C. neoformans*-infected macrophages, including downregulation of polyribonucleotide nucleotidyl transferase 1 (PNPT1) and a glutaminyl-tRNA amidotransferase subunit 1 (QRSL1) (Table S3). The PNTP1 product regulates mitochondrial homeostasis and the abundance of electron transport chain components (19). Missense mutations in the human QRSL1 locus have been associated with defects in oxidative phosphorylation (20). In addition, several aldehyde dehydrogenases were downregulated in *C. neoformans*-infected macrophages, an indicator of reduced fatty acid oxidation (Table S3).

We identified additional gene expression markers of TAG accumulation in the two infections. For example, in *M. tuberculosis* infection, we observed downregulation of lipolytic genes, upregulation of sirtuins and sirtuin-stabilizing functions, and expression changes in genes signifying increased production of ceramide and altered cellular redox. These can all lead to TAG accumulation (see Table S2). In *C. neoformans* infection, additional indicators of metabolic remodeling toward TAG biosynthesis included (i) upregulation of genes for the production of dihydroxyacetone phosphate, which can be routed toward TAG biosynthesis, (ii) upregulation of glycolytic enzymes, such as hexokinase (HK2) and lactate dehydrogenase (LDHA), that indirectly inhibit lipolysis, and (iii) downregulation of AMP-activated protein kinase (AMPK), which inhibits de novo biosynthesis of fatty acids and stimulates fatty acid oxidation (21) (see Table S3).

The transcriptomics data also shed light on the requirement in *M. tuberculosis* infection for signaling by mechanistic target of rapamycin complex 1 (mTORC1) (Fig. 2D), which is lipogenic in multiple ways (22). *M. tuberculosis*-infected macrophages showed downregulated TP53 gene and upregulated TP53-specific E3 ligases that target this factor for proteasomal degradation (see Table S2). Decreased activity of TP53 correlates well with increased mTORC1 signaling, since TP53 induces expression of Deptor (Table S2) and leads to activation of AMPK, two factors that inhibit mTORC1 (23, 24).

We conclude that, with both infections, the accumulation of TAG results from decreased oxidative phosphorylation, increased glycolysis, increased lipid biosynthesis, and decreased lipid catabolism. However, the molecular modalities of macrophage metabolic reprogramming differ between the two infections.

### Cytokine profiles differ between *C. neoformans*- and *M. tuberculosis*-infected macrophages.

Key elements of macrophage phenotypes are the cytokines and chemokines they produce when stimulated. Cytokine/chemokine production may be relevant to lipid metabolism dysregulation since, for example, lipid droplet accumulation in *M. tuberculosis*-infected macrophages requires signaling through the receptor for TNFα (5), a proinflammatory cytokine involved in lipid homeostasis (25). Indeed, treating with TNFα induces lipid droplet accumulation in human macrophages (Fig. S5). Thus, autocrine/paracrine mechanisms may be at play in lipid droplet formation. When we tested for the production of selected cytokines/chemokines during *C. neoformans* infection, we found no quantitative differences for 43 cytokines and chemokines in the culture media of non-infected vs. infected macrophages (Fig. 2G and not shown). In contrast, in response to *M. tuberculosis* infection, macrophages produced many cytokines and chemokines that were mostly proinflammatory (Fig. 2G). Thus, at least in vitro, the cytokine/chemokine milieu of macrophages infected with *C. neoformans* and *M. tuberculosis* differed greatly. That difference may contribute to the different mechanisms of lipid accumulation seen in the two infections.

### Foam cells preferentially associate with CE- and TAG-enriched kidney areas in papillary renal cell carcinoma.

**Fig. 3.**
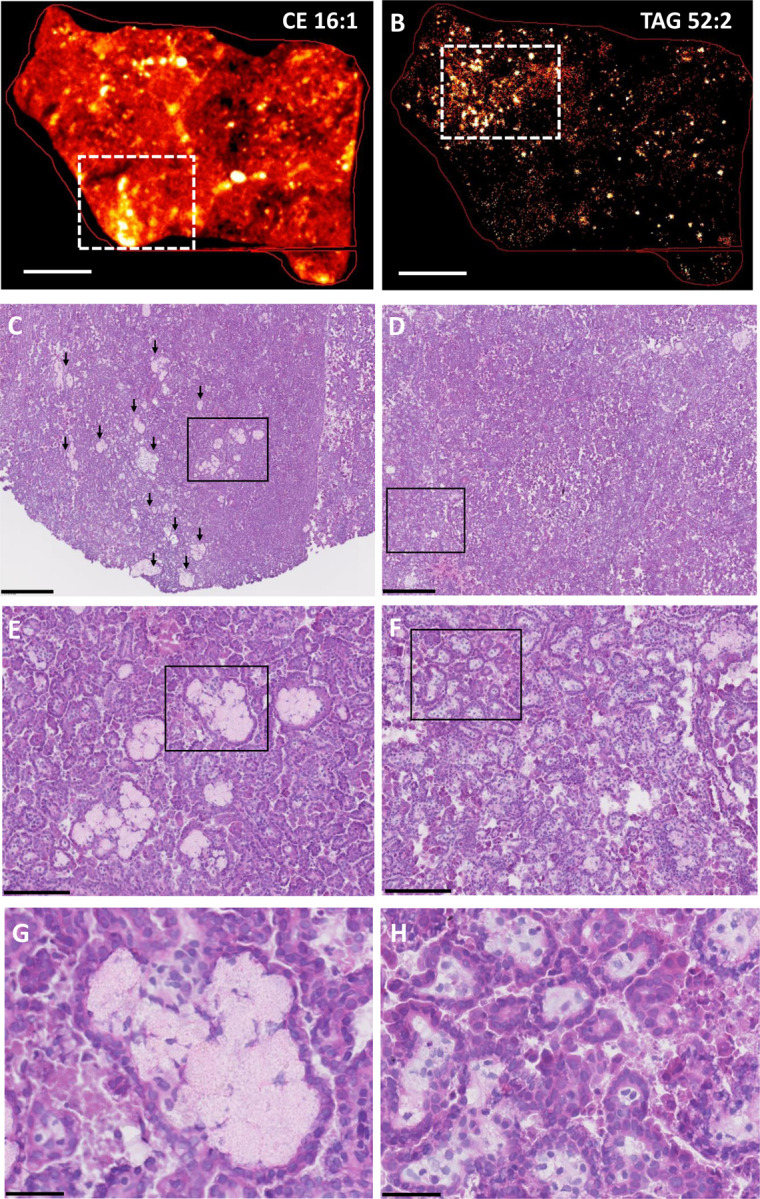
Spatial distribution of foam cells and storage lipids in papillary renal cell carcinoma (pRCC). **A-B.** MALDI imaging of representative CE and TAG species in pRCC resected tissues. MALDI-2 MSI ion distribution for CE 16:1 [M+K]^+^ *m/z* 661.532 (panel A) and TAG 52:2 [M+K]^+^ *m/z* 897.731 (panel B) are shown in frozen pRCC tissue sections; scale bar is 3 mm. The white rectangles delineate areas of high signal intensity that are magnified in the corresponding histology panels C-D. **C-H.** H&E staining of frozen pRCC tissue sections. Serial sections to those used for MALDI imaging were H&E stained. Each column corresponds to the top MALDI image (left panels, CE 16:1; right panels, TAG 52:2). The black box in each row marks the area of tissue shown at higher magnification in the corresponding panel below. **C-D**. scale bar is 500 mm. Black arrows in panel C mark large foam cell aggregates. The black box in C marks an area enriched for foam cell aggregates, which are further magnified in panel E. The black box in D marks an area enriched for foam cells interspersed among tumor cells, which is further magnified in panel F. **E-F.** scale bar is 150 mm. The black boxes in these panels mark areas further magnified in panels G and H, respectively. **G-H.** scale bar is 50 mm. Panel G shows a foam cell aggregate; panel H shows foam cells interspersed among tumor cells.

Papillary renal cell carcinoma (pRCC) is useful for studies of foam cell biogenesis in a cancer context, since foam cells are a frequent histopathologic finding in this type of cancer (15, 16). To characterize pRCC-associated foam cells, we used pRCC specimens obtained from patients who underwent partial or radical nephrectomy and performed MALDI imaging and H&E staining of adjacent sections of the resected tissues. Nine CE species were detected in the pRCC tissues. Most were distributed throughout the tissue, but their localization varied with the degree of saturation of the esterified fatty acid (Fig. S6, Table S1). In particular, the two monounsaturated species (CE 16:1 and CE 18:1), which were the most abundant in the tissues, associated with highly localized, intense signals (CE 16:1 tissue localization is shown in Fig. 3A). In contrast, TAG species yielded only localized signals, which were similar for all detected TAGs (Fig. 3B shows the distribution of TAG 52:2, which is representative of all TAG species; see Fig. S7 for other TAG species). H&E staining revealed that the intense localized CE signals corresponded to large foam cell aggregates (Fig. 3CEG show one such area at increasing magnification), while the TAG signals corresponded to tissue regions containing large numbers of foam cells interspersed among cancer cells (Fig. 3DFH show a representative area at increasing magnification). Given that CE species were detected throughout the tissue (Fig. S6), the TAG-rich regions also contained CE, albeit at lower levels than the large foam cell aggregates shown in the left panels of Fig. 3. In summary, MALDI imaging showed associations between foam-rich areas with TAG species, or CE species, or both, suggesting that, in pRCC, foam cells may accumulate either or both classes of these storage lipids.

### Factor(s) released by a papillary renal cell carcinoma-like cell line induce macrophage accumulation of both TAG and CE.

**Fig. 4.**
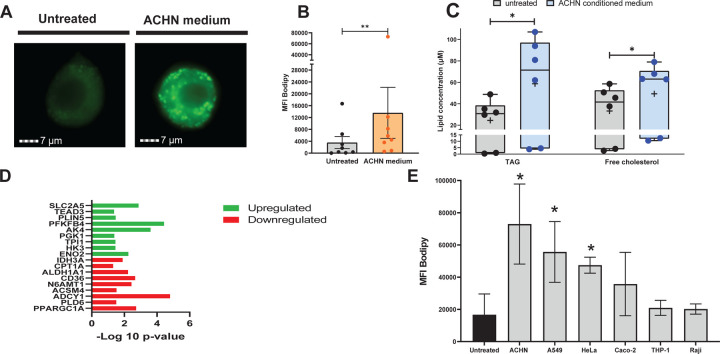
Characterization of the lipogenic effects of treatment of human primary macrophages with conditioned medium from ACHN cell cultures. Monocyte-derived macrophages (MDM) were incubated with ACHN-conditioned medium; lipid droplet and neutral lipid content was measured after 7 days of treatment. **A.** Lipid droplet content of MDM determined by imaging flow cytometry. Representative images of MDM untreated and ACHN-treated stained with Bodipy 493/503 (neutral lipid dye, green fluorescence) acquired by imaging flow cytometry. **B.** Quantification of lipid droplet content by imaging flow cytometry expressed as median fluorescence intensity (MFI) of Bodipy 493/503. Each dot represents one human donor. **C.** Neutral lipid measurements in MDM. TAG and free cholesterol were measured in infected and uninfected cells using a commercially available kit. The box plots show lower quartile, median, and upper quartile of the distribution of multiple donors. The whiskers represent minimum and maximum values. The plus symbol indicates the mean. *, *p* < 0.05; **, *p* < 0.01 (Wilcoxon signed-rank test). **D.** Gene expression responses to ACHN medium treatment. Differential expression (treated vs untreated) of metabolism-related genes was ranked according to their significance level. **E.** MDM treatment with conditioned media from various cancer cell lines. MDM were treated with conditioned media from various cancer cell lines, as indicated. Lipid droplet content was measured by imaging flow cytometry, as in panel A, and expressed as MFI of Bodipy 493/503.

We next investigated the effects of pRCC on storage lipid accumulation in macrophages in vitro by exposing human macrophages to cell-free conditioned medium from cultures of the ACHN cell line, which is derived from a human renal cell carcinoma and exhibits pRCC features (26). Exposure to ACHN-conditioned medium induced macrophage production of chemokines and cytokines, including IL-8, IL-6, CCL2, and CXCL16 (Fig. S8A), which are characteristic of the response of tumor-associated macrophages to factors released by cancer cells (27–29). ACHN-medium-treated macrophages also exhibited lipid droplet accumulation (Fig. 4AB), in agreement with previous observations (13), indicating the presence in the medium of lipogenic bioproduct(s) of the ACHN cells. Since IL-8 produced by cultured pRCC cells has been proposed as a foam-cell-inducing factor (13), we investigated the relationship among pRCC, foam cell formation, and IL-8 in our experimental system. We found that IL-8 is lipogenic for macrophages, but at a concentration ~10-fold higher than that measured in the ACHN-conditioned medium (~0.5ng/ml) (Fig. S8BC and Table S4). Moreover, blocking IL-8 activity with neutralizing antibodies did not prevent lipid droplet formation in macrophages exposed to ACHN medium, and IL-8 depletion from the ACHN medium had no effect on lipid droplet formation (Fig. S8D-F). Thus, the lipogenic effect of the ACHN medium cannot be ascribed to IL-8. We cannot exclude, however, that IL-8 is lipogenic via paracrine-autocrine mechanisms in the tumor microenvironment in vivo since, for example, ACHN-medium-treated macrophages produce lipogenic concentrations of this cytokine (~5ng/ml) (Fig. S8A and Table S4).

Storage lipid analysis by enzymatic assays showed that lipid droplet accumulation in ACHN-medium-treated macrophages correlated with increased levels of TAG and free cholesterol (Fig. 4C), suggesting yet another context-specific mechanism of foam cell formation. Indeed, metabolism-related gene expression analysis identified mechanisms for both TAG and cholesterol accumulation. Genes encoding enzymes associated with glycolysis were upregulated (Fig. 4D and Table S5). These included triosephosphate isomerase 1 (TPI1), which catalyzes the conversion of glyceraldehyde-3P to the TAG precursor dihydroxy-acetone phosphate, and enolase 2 (ENO2), which converts phosphoenolpyruvate to pyruvate (Fig. 4D). We also found gene markers of reduced TCA cycle, including upregulation of adenylate kinase 4 (AK4), a key metabolic regulator increasing glycolysis and inhibiting TCA cycle and oxidative phosphorylation (30) and downregulation of PPARGC1A. The latter gene encodes PGC-1α, a master regulator of energy metabolism that promotes fatty acid oxidation and TCA cycle and decreases TAG storage (31) (Fig. 4D). Together, increased glycolysis and reduced TCA cycle would result in routing of pyruvate towards de novo lipogenesis. Additional gene expression markers of TAG accumulation included the upregulation of perilipin 5 (PLIN5) (Fig. 4D), a member of a protein family protecting TAG from enzymatic degradation (32), and downregulation of the phospholipase PLD6 (Fig. 4D), which inhibits YAP/TAZ mediated lipogenesis (33). Moreover, reduced fatty acid oxidation might result from the downregulation of the acyl-CoA synthase ACSM4 (Fig. 4D) and genes expressing associated functions, such as acyl-CoA conversion to acyl-carnitine (carnitine palmitoyltransferase I, CPT1) and oxidation of fatty aldehydes to fatty acids (aldehyde hydrogenases, ALDH genes) (Fig. 4D and Table S5). The downregulation of N6AMT1 (Fig. 4D), which encodes a methyltransferase mostly implicated in DNA and protein modification, is also consistent with cellular lipid accumulation, since it has been associated with lipid catabolism (34) by yet unknown mechanisms.

The ACHN-medium-treated macrophages also exhibited gene expression changes associated with dysregulation of cholesterol metabolism. For example, the scavenger receptor CD36, which is a key regulator of cholesterol homeostasis, was downregulated (Table S5), presumably as a consequence of PPARGC1A downregulation (Fig. 4D) (31). CD36 induces cholesterol depletion by promoting macrophage cholesterol efflux and proteasomal degradation of HMG-CoA reductase, the rate-limiting enzyme in sterol synthesis (35). An additional marker of dysregulated cholesterol homeostasis is the downregulation of adenylate cyclase (ADCY1), which generates cAMP signaling for cholesterol efflux in atherogenic foam cells (36).

Collectively, gene expression data point toward macrophage metabolic reprogramming in response to factors in the ACHN-conditioned medium. This reprogramming includes increased glycolysis, impaired TCA cycle and oxidative metabolism, decreased lipolysis, and dysregulated cholesterol homeostasis.

### Conditioned media from several cancer cell lines induce lipid droplets in macrophages.

As mentioned above, foam cells have been seen in several cancers (11, 12, 14). To begin to examine the relationship between foam cells and cancers other than pRCC, we tested the lipid-droplet-inducing ability of conditioned medium obtained from cultures of various cell lines of cancerous origin. We found that, in addition to that of ACHN cells, cell-free conditioned media of A549 (lung epithelial) and HeLa (cervical epithelial) cancer cells were lipogenic (Fig. 4E). The effect is cancer-type-specific, since conditioned media from other cancer cell lines, such as Caco-2 (colon carcinoma), THP-1 (acute monocytic leukemia), and Raji (B-cell lymphoma), did not induce lipid droplet formation in macrophages (Fig. 4E). These results show that some cancer cells release lipogenic factors for macrophages, suggesting that certain tumor microenvironments may induce lipid accumulation in tumor-associated macrophages.

## Discussion

The data reported above show that the mechanisms of foam cell biogenesis differ with disease context. That is the case regardless of the chemical nature of the storage lipids they accumulate. For example, macrophages infected with *M. tuberculosis* and *C. neoformans* are enriched in TAG. In both cases, the accumulation of TAG results from a switch from oxidative to glycolytic metabolism, including increased biosynthesis and decreased catabolism of lipids. However, the molecular mechanisms underlying the metabolic reprogramming of macrophages differ between the two infections, as indicated by the transcriptome analyses and by the different effect of rapamycin on lipid droplet accumulation in the two infections. Still other mechanisms are found in pRCC-associated macrophages, which accumulate neutral lipids by reprogramming both cholesterol and TAG metabolism. Therefore, while foam cells are similar across disease contexts histochemically (lipid droplets are stained by Oil Red O and confer a “pale bubbly” appearance at H&E staining) and perhaps even functionally (discussed below), they are mechanistically heterogeneous, since multiple cellular pathways can lead to increased lipid uptake and anabolism and/or decreased catabolism.

A key to understanding foam cell heterogeneity is the biochemical diversity of the microenvironments driving their biogenesis. It is well established that uptake of exogenous lipids can drive foam cell formation. This is the case in atherogenesis, where sequestration of cholesterol-rich lipoproteins in the arterial wall leads to endothelial activation, recruitment of monocytes, and monocyte differentiation into lipoprotein-ingesting phagocytes that become foam cells (3). Moreover, in some cancers, such as colon cancer, the fatty acid-enriched environment induces lipid droplet accumulation in tumor-associated macrophages (14). It would be fallacious, however, to associate foam cell formation exclusively with exogenous lipid uptake. Instead, our work points to additional scenarios in which various microenvironment-specific signals trigger a metabolic remodeling of local macrophages that leads to excess intracellular fatty acids that are detoxified by storage in droplets. These environmental triggers may include pathogen-derived molecules, cancer cell products, inflammatory signals from activated and/or damaged cells that, for example, activate Toll-like receptors (TLR) (or other pattern recognition receptors) resulting in reshaping of the macrophage lipidome (37). Additional heterogeneity may result from the combinatorial effects of multiple foam-cell-inducing signals in some microenvironments. For example, foam cells may be induced in pRCC by yet unidentified cancer cell products, as indicated by our results with ACHN-conditioned medium, together with lipogenic proinflammatory cytokines, such as IL-8, produced by tumor-associated-macrophages. Moreover, additional mediators might be produced by cell types that are not represented in our in vitro system, such as the lipid-filled tumor cells that we observed in the pRCC bioptic tissue. In tuberculosis, we showed that, in addition to bacterial components that presumably trigger TLR2 signaling (38, 39), macrophage lipid accumulation requires another lipogenic proinflammatory cytokine, TNFα, produced by infected macrophages (5). Additional work is needed to identify the exogenous (i.e., generated by microbes, cancer cells, or other cell types) trigger signals and to determine how commonly foam cells are induced by combinations of exogenous and autocrine/paracrine signals.

It is reasonable to assume that, despite the different mechanisms of foam cell biogenesis, their presence represents a maladaptive immune response in the pathological contexts they form. Generally, lipid-laden macrophages tend to lose protective immune functions, including phagocytosis, efferocytosis, and autophagy. They can also induce tissue damage, contribute to necrosis, exhibit impaired antimicrobial activity, and even sustain survival of intracellular pathogens (reviewed in (1)). Indeed, given their contribution to pathogenesis, foam cells have been recognized as targets of pharmacological intervention, for example in atherosclerosis and some cancers (14, 40). Moreover, foam cells are often associated with kidney disease, such as focal and segmental glomerulosclerosis and diabetic nephropathy (41), in addition to the pRCC investigated in the present work. Their pathophysiological significance in the kidney remains puzzling, and all mechanistic hypotheses on their biogenesis derive from the atherosclerosis literature (41). Recognizing that foam cells do not result only from macrophage uptake of exogenous lipids and that the mechanisms of their formation are microenvironment-specific opens new directions for mechanistic and drug development research of high biomedical significance.

## Supplementary Material

Supplement 1

## Significance statement.

Chronic inflammatory states of infectious and non-infectious etiology are associated with dysfunctional immune responses. Primary contributors are foam cells, lipid-laden macrophages exhibiting impaired or pathogenic immune functions. In contrast with the long-standing paradigm derived from atherosclerosis in which foam cells are cholesterol-laden, our work demonstrates that foam cells are heterogeneous. Utilizing bacterial, fungal, and cancer models, we show that foam cells may accumulate various storage lipids (triglycerides and/or cholesteryl esters) by mechanisms that depend on disease-specific microenvironments. Thus, we present a new framework for foam cell biogenesis in which the atherosclerosis paradigm represents only a specific case. Since foam cells are a potential therapeutic target, understanding their mechanisms of biogenesis will provide knowledge needed for novel therapeutic approaches.

## Abbreviations:

CE: cholesteryl esters
H&E: hematoxylin and eosin
MALDI: matrix-assisted laser desorption/ionization mass spectrometry
MDM: monocyte-derived macrophages
pRCC: papillary renal cell carcinoma
TAG: triglycerides
