# Heterogeneity of foam cell biogenesis across diseases

**DOI:** 10.1101/2023.06.08.542766

**Published:** 2024-04-20

**Authors:** Valentina Guerrini, Brendan Prideaux, Rehan Khan, Selvakumar Subbian, Yina Wang, Evita Sadimin, Siddhi Pawar, Rahul Ukey, Eric A. Singer, Chaoyang Xue, Maria Laura Gennaro

**Affiliations:** 1 Public Health Research Institute, Rutgers New Jersey Medical School, Rutgers Biomedical and Health Sciences, Newark, NJ 07103; 2 Department of Medicine, Rutgers New Jersey Medical School, Rutgers Biomedical and Health Sciences, Newark, NJ 07103; 3 Department of Microbiology, Rutgers New Jersey Medical School, Rutgers Biomedical and Health Sciences, Newark, NJ 07103; 4 Department of Neurobiology, University of Texas Medical Branch, Galveston, TX 77555; 5 Section of Urologic Pathology and Rutgers Cancer Institute of New Jersey and Rutgers Robert Wood Johnson Medical School, New Brunswick, NJ 08901; 6 Section of Urologic Oncology, Rutgers Cancer Institute of New Jersey and Rutgers Robert Wood Johnson Medical School, New Brunswick, NJ 08901

**Keywords:** kidney disease, tuberculosis, cryptococcosis, papillary renal cell carcinoma, lipid metabolism, storage lipids

## Abstract

Foam cells are dysfunctional, lipid-laden macrophages associated with chronic inflammation of diverse origin. The long-standing paradigm that foam cells are cholesterol-laden derives from atherosclerosis research. We previously showed that, in tuberculosis, foam cells surprisingly accumulate triglycerides. Here, we utilized bacterial (*Mycobacterium tuberculosis*), fungal (*Cryptococcus neoformans*), and human papillary renal cell carcinoma (pRCC) models. We applied mass spectrometry-based imaging to assess the spatial distribution of storage lipids relative to foam-cell-rich areas in lesional tissues, and characterized lipid-laden macrophages generated under corresponding *in vitro* conditions. The *in vivo* data were consistent with in vitro findings showing that cryptococcus-infected macrophages accumulated triglycerides, while macrophages exposed to pRCC-conditioned-medium accumulated both triglycerides and cholesterol. Moreover, cryptococcus- and mycobacterium-infected macrophages accumulated triglycerides by different mechanisms. Collectively, our data indicate that the mechanisms of foam cell formation are disease-microenvironment-specific. Since foam cells are potential therapeutic targets, recognizing that their formation is disease-specific opens new biomedical research directions.

Chronic inflammation of infectious and non-infectious origin is often associated with the presence of foam cells, lipid-laden macrophages that exhibit impaired immune function and can contribute to pathogenesis [1]. Foam cells (or foamy macrophages) form when, due to dysregulated metabolism, lipids accumulate beyond the homeostatic capacity of macrophages. The lipids are stored as droplets that confer a foamy appearance to the macrophages [2]. Our understanding of foam cell biology has been largely based on studies of atherogenesis, a disease in which uptake of normal and proinflammatory lipoproteins by macrophages in the arterial wall leads to imbalanced cholesterol metabolism and formation of cholesterol-laden foam cells [3]. The accumulation of foam cells in the arterial intima leads to chronic inflammation, cell death, and tissue necrosis [3]. A similar situation is observed in tuberculosis, a chronic inflammatory disease of the lung caused by *Mycobacterium tuberculosis*. In the tuberculous lung lesions, which are called granulomas, the presence of tissue necrosis is associated with foam cell accumulation [1]. Indeed, foam cells are a hallmark of both the atherosclerotic plaque and the necrotizing tuberculous granuloma [3, 4]. We were surprised to find that the foam cells of necrotic tuberculous lung lesions are enriched in triglycerides (TAG), as demonstrated by laser-capture microdissection and mass spectrometry analysis of lesional tissue [5]. Work with cultured human macrophages and mice further established that *M. tuberculosis* infection induces TAG accumulation in macrophages [5, 6]. Thus, the atherosclerosis and tuberculosis models of foam cells are fundamentally different, indicating that foam cells may form via different mechanisms in different diseases. To test the hypothesis that foam cell biogenesis is disease-specific, we began a study of foam cells resulting from another infectious disease, cryptococcosis, and from a form of cancer. Cryptococcosis is a clinically heterogeneous disease caused by the fungal pathogen *Cryptococcus neoformans*. It affects the lung and other organ systems, including the central nervous system, particularly in immunocompromised individuals [7]. Foamy macrophages have been observed in human tissue biopsies from pulmonary and extrapulmonary cryptococcosis [8–10] and in the lungs of infected mice [11]. Foam cells have also been associated with several forms of cancer of many organ systems that include liver, lung, colon/rectum, and kidney [12–15]. Indeed, the presence of foamy macrophages is a histological feature of papillary renal cell carcinoma (pRCC) [16, 17]. Factors released by cultured pRCC cells induce lipid accumulation in macrophages [14], indicating that the microenvironment of this tumor is lipogenic for macrophages. The nature of storage lipids and the mechanism of foam cell formation are poorly understood in these pathologies.

In the present work, we assessed the spatial distribution of foamy macrophages and storage lipids in *C. neoformans*-infected murine lungs and in human pRCC specimens. We then analyzed lipid content and the transcriptional program of lipid-laden macrophages generated under *in vitro* conditions that corresponded to these two diseases. The data established that the mechanism underlying foam cell formation varies with disease context. We can no longer base our understanding of foam cell biogenesis only on work focused on atherogenesis. Expanding our view of foam cell biogenesis may provide new targets for therapeutic intervention into diseases -- such as atherosclerosis, tuberculosis, multiple sclerosis, and certain cancers -- in which foam cell appearance and poor clinical outcome are associated (reviewed in [1]).

## Results

### Foamy macrophages cluster peri- or extra-lesionally and associate with TAG species in *C. neoformans*-infected murine lungs.

**Fig. 1.**
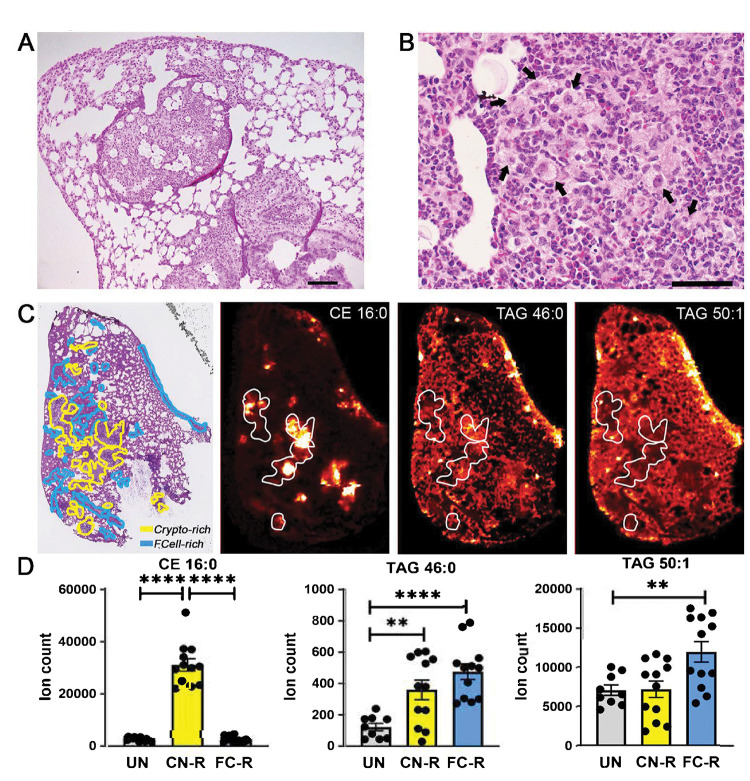
Spatial distribution of foam cells and storage lipids in *C. neoformans*-infected murine lungs. **A-B.** H&E staining of formalin-fixed, paraffin-embedded lung sections from *C. neoformans* H99-infected mice. Images were photographed at (A) 100x magnification; scale bar is 100 μm; and (B) 400x magnification; scale bar is 10 μm. Black arrows indicate foam cells. **C.** MALDI imaging of representative CE and TAG species in infected lung sections. The left panel shows H&E staining of infected tissue sections (scale bar is 2 mm). The yellow lines delineate areas enriched in fungal cells (Crypto-rich), while the blue lines define areas enriched in foam cells (FCell-rich). The three additional panels show MALDI imaging of storage lipids in lung sections contiguous to those used for H&E staining. Representative species are shown: CE (16:0) [M+K]^+^ *m/z* 663.48 and TAG (46:0) [M+K]^+^ *m/z* 817.669 signals tend to correspond to cryptococci-enriched areas, while TAG (50:1) [M+K]^+^ *m/z* 871.716 tends to be reduced in those same areas. Areas delimited by white lines correspond to some cryptococci-enriched areas in the H&E-stained section. Corresponding images of uninfected lung sections are shown in Fig. S2. **D.** Quantification of CE and TAG MALDI imaging intensity (expressed as ion count). Quantification of lipid species was performed in uninfected tissue and in fungus-rich (CN-R) and foam-cell-rich (FC-R) areas of the infected tissue. Mean and SEM of 9 sections from three uninfected animals (3 sections per animal) and 12 sections from four infected animals (3 sections per animal) are shown. **, *p* < 0.05; ****, *p* < 0.001 (unpaired *t*-test).

Foamy macrophages form during pulmonary and extrapulmonary cryptococcal infection [8, 9]. We used a model of pulmonary cryptococcosis in C57BL/6 mice to assess the spatial relationship between foam cells and neutral lipids [TAG and cholesteryl esters (CE)] in infected lungs. At 7 days post intranasal infection, infected mouse lungs exhibited several granulomatous nodular lesions visible at low magnification (Fig. S1). The lesions consisted of large aggregates of fungal cells surrounded by inflammatory infiltrates comprised mostly of polymorphonuclear cells, macrophages and lymphocyte aggregates, and epithelioid cells (Fig. 1A). Morphologically, macrophages contained small nuclei and cytoplasmic lipid droplets giving them a foamy/bubbly appearance. These foam cells tended to form clusters in peri- or extra-lesional areas of the infected foci in the lungs [hematoxylin and eosin (H&E)-stained lung slices in Fig. 1BC]. When we used matrix-assisted laser desorption/ionization mass spectrometry (MALDI) imaging of sections adjacent to those used for H&E staining, we detected multiple TAG and CE species in the infected lungs (Table S1). All CE species localized in the fungus-rich lesions (e.g., compare H&E staining and MALDI imaging for CE 16:0 in Fig. 1C). In contrast, TAG species were distributed throughout the lung tissue, with some species, such as TAG 46:0, more prominently found within the lesions and others, such as TAG 50:1, found extra-lesionally (Fig. 1C, with corresponding ion counts in Fig. 1D) (see Fig. S2 for uninfected control tissue). Localization of some TAG species and CE species in the fungus-rich lesions is consistent with the presence of TAG and sterols in fungal cells [18, 19]. In addition, the spatial distribution of some TAG species, such as TAG 50:1 in Fig. 1C, which was present throughout the tissue but approximately two-fold lower in the fungus-rich lesions, corresponds to that of foam cells (compare H&E staining and MALDI imaging in Fig. 1C), suggesting that *Cryptococcus*-induced foam cells are TAG enriched.

### *Cryptococcus neoformans* infection induces accumulation of TAG-rich lipid droplets in macrophages via an mTORC1-independent pathway.

**Fig. 2.**
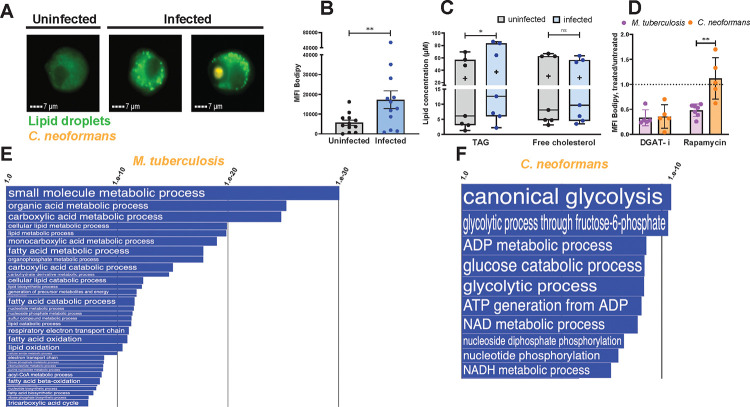
Characterization of lipid droplets induced by *C. neoformans* infection and comparisons with *M. tuberculosis*-induced effects. **A.** Lipid droplet content of MDM determined by imaging flow cytometry. Representative images of MDM uninfected (leftmost panel) and infected with mCherry-tagged *C. neoformans*. The two rightmost panels show macrophages in the infected culture wells carrying and not carrying intracellular fungi (orange fluorescence). Cells were stained with Bodipy 493/503 (neutral lipid dye, green fluorescence). Images were acquired by imaging flow cytometry at 24 hrs post-infection. **B.** Quantification of lipid droplet content by imaging flow cytometry expressed as median fluorescence intensity of Bodipy 493/503. Each dot represents one human donor. **C.** Neutral lipid measurements in MDM. TAG and cholesterol were measured in infected and uninfected cells using a commercially available kit. The box plots show lower quartile, median, and upper quartile of the distribution of multiple donors. The whiskers represent minimum and maximum values. The plus symbol indicates the mean. ns, non-significant; *, *p* < 0.05 (paired *t*-test). Each dot represents one human donor. **D.** Effect on lipid droplets of MDM treatment with a DGAT inhibitor (DGAT-i) or rapamycin (mTORC1 inhibitor). MDM were infected with *C. neoformans* or with *M. tuberculosis* for 24h with an MOI = 4 in both infections. DMSO (vehicle control), 0.4 nM rapamycin, or 30 nM DGAT-1 inhibitor (A922500; PubChem CID: 24768261) were added for the duration of infection. Lipid droplet content was quantified by imaging flow cytometry and expressed as Bodipy MFI, as described in panel A and in Fig. S3AB. Results are shown as ratios of Bodipy MFI of drug-treated to vehicle-treated infected cells. Mean and SD are shown. ns, non-significant; **, *p* < 0.01 (unpaired *t*-test). Each dot represents one human donor. **E-F.** Pathway analysis of the transcriptomes of *M. tuberculosis*- and *C. neoformans-*infected MDM. The two panels show Gene Ontology (GO) annotations related to metabolic processes that were (**E**) downregulated in *M. tuberculosis*-infected MDM and (**F**) upregulated in *C. neoformans*-infected MDM, relative to uninfected control cells. The differential expression between sample classes (infected vs uninfected) was tested with coincident extreme ranks in numerical observations (CERNO). Pathways were selected using a cutoff false discovery rate of 0.05; the *p*-values for these pathways are plotted onto the x-axis. To represent effect size, pathway gene sets containing fewer genes were given greater bar height/font size than were larger sets that yielded similar *p* values. For visualization purposes, top ranking annotations are shown. Additional pathway analysis data are shown in Supplementary Fig. S4.

MALDI imaging provides information about the spatial distribution of analytes in tissues, but it does not have the single-cell resolution needed to precisely assign a particular neutral lipid to a specific cell type. Thus, we utilized an in vitro infection model to study neutral lipid accumulation in macrophages infected with *C. neoformans*. We infected primary human monocyte-derived macrophages (MDM) with mCherry-expressing *C. neoformans* H99, quantified lipid droplet content by imaging flow cytometry, and observed a significant lipid droplet accumulation in infected macrophages (3.5-fold increase relative to uninfected cells) (Fig. 2AB). Lipid-droplet-enriched macrophages in the infected culture wells included both those containing fungal cells and those that did not (Fig. 2A and quantitative data in Fig. S3A). These data indicated that lipid droplet formation does not require internalization of fungal cells. Since lipid droplets accumulated in macrophages exposed to cell-free *C. neoformans* culture filtrate (Fig. S3B) but not in macrophages exposed to heat-killed fungi (Fig. S3C), lipid droplet accumulation in macrophages appears to involve a factor(s) released from live *C. neoformans* cells.

When we measured storage lipid content in *C. neoformans*-infected cells by an enzymatic assay, we found that infection increased the content of intracellular TAG but not cholesterol derivatives (Fig. 2C). Moreover, lipid droplet accumulation in *C. neoformans*-infected cells was essentially abrogated by treatment with an inhibitor of diglyceride acetyl transferase (DGAT), the enzyme that catalyzes the conversion of diglycerides to triglycerides (Fig. 2D). This finding supports the conclusion that *C. neoformans*-induced lipid droplets are TAG enriched, as previously seen with *M. tuberculosis* infection ([5] and Fig. 2D).

Our previous work showed that the accumulation of TAG-rich lipid droplets in macrophages infected with *M. tuberculosis* requires mTORC1 signaling, as it is inhibited by rapamycin treatment [5]. Unlike the *M. tuberculosis* case, however, rapamycin had no effect on lipid droplet accumulation in *C. neoformans-*infected macrophages (Fig. 2D). Thus, even though *M. tuberculosis* and *C. neoformans* both induce accumulation of TAG-rich lipid droplets, the two pathogens do so by utilizing different signaling mechanisms.

### Transcriptomics identify different molecular mechanisms of TAG accumulation in *M. tuberculosis*- and *C. neoformans*-infected macrophages.

We investigated the pathways underlying TAG accumulation in *M. tuberculosis*- and *C. neoformans-* infected macrophages by conducting transcriptomics analyses of macrophages obtained from the same donors and infected in vitro with either pathogen. When we analyzed the Gene Ontology (GO) annotations related to metabolic processes, we found that the most informative signatures of macrophage metabolic reprogramming associated with TAG accumulation derived from the downregulated pathways in *M. tuberculosis*-infected macrophages and from the upregulated pathways in *C. neoformans*-infected macrophages. The former included lipid catabolism, fatty acid oxidation, oxidative phosphorylation, and electron transport chain (Fig. 2E), while the latter were enriched for glycolysis (Fig. 2F).

Metabolism-related analyses at the gene level (Table S2 and Supplementary text) showed that, in *M. tuberculosis*-infected macrophages, the top three downregulated metabolic genes encoded: (i) acyl-CoA synthase (ACSM5), (ii) carnitine octanoyl transferase (CROT), which converts acyl-CoA to acyl-carnitine, a step required for transport across the mitochondrial membrane, and iii) aldehyde hydrogenase (ALDH3A2), which oxidizes fatty aldehydes to fatty acids. Downregulation of these genes likely leads to defective fatty acid oxidation. In *C. neoformans* infection, the top five upregulated metabolic genes all encoded glycolytic enzymes (Table S3 and Supplementary text). We also found indicators of reduced mitochondrial functions in *C. neoformans*-infected macrophages, including downregulation of polyribonucleotide nucleotidyl transferase 1 (PNPT1) and a glutaminyl-tRNA amidotransferase subunit 1 (QRSL1) (Table S3). The PNTP1 product regulates mitochondrial homeostasis and the abundance of electron transport chain components [20]. Missense mutations in the human QRSL1 locus have been associated with defects in oxidative phosphorylation [21]. In addition, several aldehyde dehydrogenases were downregulated in *C. neoformans*-infected macrophages, an indicator of reduced fatty acid oxidation (Table S3 and Supplementary text).

We identified additional gene expression markers of TAG accumulation in the two infections. For example, in *M. tuberculosis* infection, we observed downregulation of lipolytic genes, upregulation of sirtuins and sirtuin-stabilizing functions, and expression changes in genes signifying increased production of ceramide and altered cellular redox. These can all lead to TAG accumulation (see Table S2 and Supplementary text). In *C. neoformans* infection, additional indicators of metabolic remodeling toward TAG biosynthesis included (i) upregulation of genes for the production of dihydroxyacetone phosphate, which can be routed toward TAG biosynthesis, (ii) upregulation of hexokinase (HK2) and lactate dehydrogenase (LDHA), which indirectly inhibit lipolysis, and (iii) downregulation of AMP-activated protein kinase (AMPK), which inhibits de novo biosynthesis of fatty acids and stimulates fatty acid oxidation [22] (see Table S3). These and additional markers of lipid accumulation are discussed further in the Supplementary text.

The transcriptomics data also shed light on the requirement in *M. tuberculosis* infection for signaling by mechanistic target of rapamycin complex 1 (mTORC1) (Fig. 2D), which is lipogenic in multiple ways [23]. *M. tuberculosis*-infected macrophages showed downregulated TP53 gene and upregulated TP53-specific E3 ligases that target this factor for proteasomal degradation (see Table S2 and Supplementary text). Decreased activity of TP53 correlates well with increased mTORC1 signaling, since TP53 induces expression of Deptor (Table S2) and leads to activation of AMPK, two factors that inhibit mTORC1 [24, 25].

We conclude that, with both infections, the accumulation of TAG results from decreased oxidative phosphorylation, increased glycolysis, increased lipid biosynthesis, and decreased lipid catabolism. However, the molecular modalities of macrophage metabolic reprogramming differ between the two infections.

### Foamy macrophages preferentially associate with CE- and TAG-enriched kidney areas in papillary renal cell carcinoma.

**Fig. 3.**
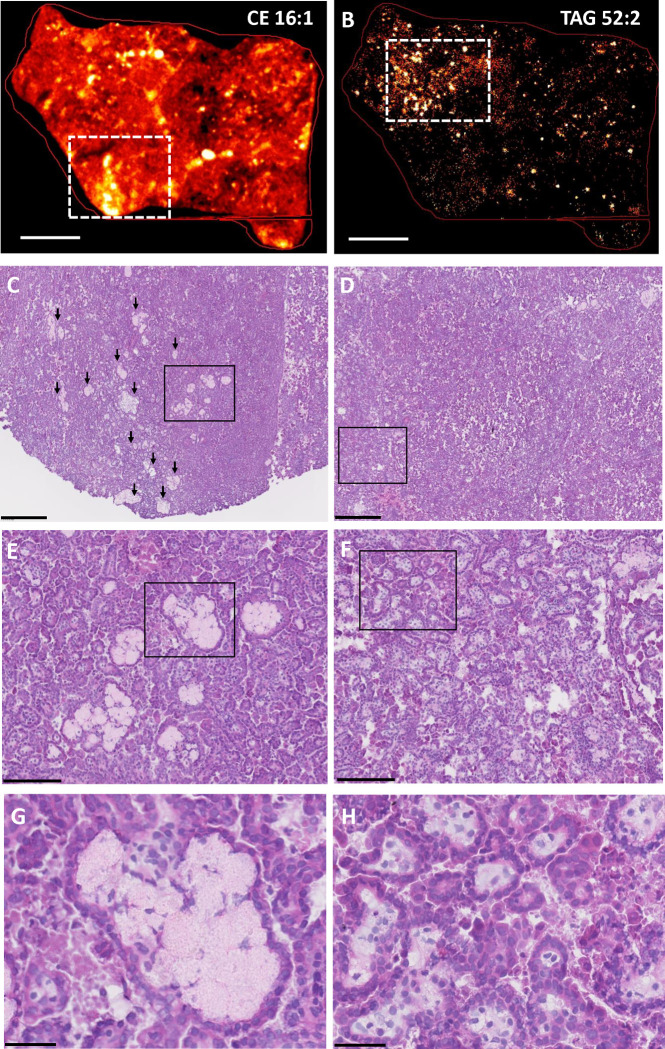
Spatial distribution of foam cells and storage lipids in papillary renal cell carcinoma (pRCC). **A-B.** MALDI imaging of representative CE and TAG species in pRCC resected tissues. MALDI-2 MSI ion distribution for CE 16:1 [M+K]^+^ *m/z* 661.532 (panel A) and TAG 52:2 [M+K]^+^ *m/z* 897.731 (panel B) are shown in frozen pRCC tissue sections; scale bar is 3 mm. The white rectangles delineate areas of high signal intensity that are magnified in the corresponding histology panels C-D. **C-H.** H&E staining of frozen pRCC tissue sections. Serial sections to those used for MALDI imaging were H&E stained. Each column corresponds to the top MALDI image (left panels, CE 16:1; right panels, TAG 52:2). The black box in each row marks the area of tissue shown at higher magnification in the corresponding panel below. **C-D**. scale bar is 500 mm. Black arrows in panel C mark large foam cell aggregates. The black box in C marks an area enriched for foam cell aggregates, which are further magnified in panel E. The black box in D marks an area enriched for foam cells interspersed among tumor cells, which is further magnified in panel F. **E-F.** scale bar is 150 mm. The black boxes in these panels mark areas further magnified in panels G and H, respectively. **G-H.** scale bar is 50 mm. Panel G shows a foam cell aggregate; panel H shows foam cells interspersed among tumor cells.

Papillary renal cell carcinoma (pRCC) is useful for studies of foam cell biogenesis in a cancer context, since foam cells are a frequent histopathologic finding in this type of cancer [16, 17]. To characterize pRCC-associated foam cells, we used pRCC specimens obtained from patients who underwent partial or radical nephrectomy and performed MALDI imaging and H&E staining of adjacent sections of the resected tissues. The foamy macrophages, which are characterized morphologically by foamy/bubbly cytoplasm and small nuclei, were seen interspersed throughout the inter-tumoral stroma. Nine CE species were detected in the pRCC tissues. Most were distributed throughout the tissue, but their localization varied with the degree of saturation of the esterified fatty acid (Fig. S5, Table S1). In particular, the two monounsaturated species (CE 16:1 and CE 18:1), which were the most abundant in the tissues, associated with highly localized, intense signals (CE 16:1 tissue localization is shown in Fig. 3A). In contrast, TAG species yielded only localized signals, which were similar for all detected TAGs (Fig. 3B shows the distribution of TAG 52:2, which is representative of all TAG species; see Fig. S6 for other TAG species). H&E staining revealed that the intense localized CE signals corresponded to large foam cell aggregates (Fig. 3CEG show one such area at increasing magnification), while the TAG signals corresponded to tissue regions containing large numbers of foam cells interspersed among cancer cells (Fig. 3DFH show a representative area at increasing magnification). Given that CE species were detected throughout the tissue (Fig. S6), the TAG-rich regions also contained CE, albeit at lower levels than the large foam cell aggregates shown in the left panels of Fig. 3. In summary, MALDI imaging showed associations between foam-rich areas with TAG species, CE species, or both, suggesting that, in pRCC, foam cells may accumulate either or both classes of these storage lipids. Bioptic tissues collected from two additional patients showed similar differential distribution of TAG and CE species in pRCC foam cells, with more intense TAG signals associated with large necrotic regions (Fig. S7).

### Factor(s) released by a papillary renal cell carcinoma-like cell line induce macrophage accumulation of both TAG and CE.

**Fig. 4.**
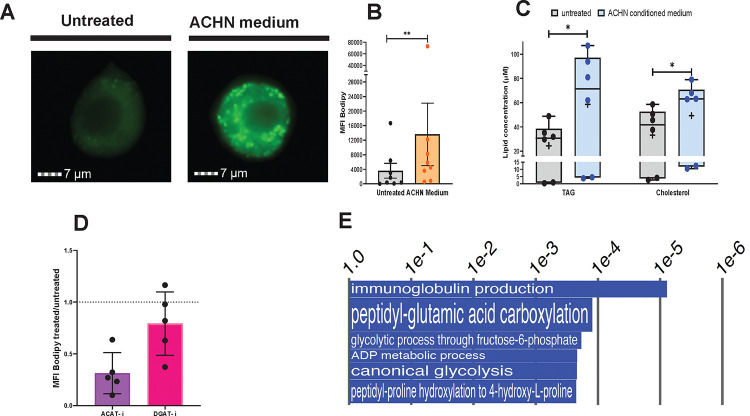
Characterization of the lipogenic effects of treatment of human primary macrophages with conditioned medium from ACHN cell cultures. Monocyte-derived macrophages (MDM) were incubated with ACHN-conditioned medium; lipid droplet and neutral lipid content was measured after 7 days of treatment. **A.** Lipid droplet content of MDM determined by imaging flow cytometry. Representative images of MDM untreated and ACHN-treated stained with Bodipy 493/503 (neutral lipid dye, green fluorescence) acquired by imaging flow cytometry. **B.** Quantification of lipid droplet content by imaging flow cytometry expressed as median fluorescence intensity (MFI) of Bodipy 493/503. Each dot represents one human donor. **C.** Neutral lipid measurements in MDM. TAG and cholesterol were measured in infected and uninfected cells using a commercially available kit. The box plots show lower quartile, median, and upper quartile of the distribution of multiple donors. The whiskers represent minimum and maximum values. The plus symbol indicates the mean. *, *p* < 0.05; **, *p* < 0.01 (Wilcoxon signed-rank test). D. Effect on lipid droplets of MDM treatment with a DGAT inhibitor and an ACAT inhibitor. MDM were treated with ACHN-conditioned medium containing DMSO (vehicle control), 90 nm DGAT-1 inhibitor (DGAT-i) (A922500; PubChem CID:24768261), or 10 μM ACAT inhibitor (ACAT-i) (PubChem CID:10019206) for 7 days. Lipid droplet content was quantified by imaging flow cytometry and expressed as Bodipy MFI,as described in Fig. 2A. Results are shown as ratios of Bodipy MFI of drug-treated to vehicle-treated cells. Mean and SD are shown. ns, non-significant; **, *p* < 0.01 (unpaired *t*-test). Each dot represents one human donor. E**.** Pathway analysis of the transcriptome of ACHN-medium-treated MDM. The panel shows Gene Ontology (GO) annotations related to metabolic processes that were upregulated in ACHN-medium-treated MDM, relative to untreated control cells. The differential expression between sample classes (infected vs uninfected) was tested with coincident extreme ranks in numerical observations (CERNO). Pathways were selected using a cutoff false discovery rate of 0.055; the *p*-values for these pathways are plotted onto the x-axis. To represent effect size, pathway gene sets containing fewer genes were given greater bar height/font size than were larger sets that yielded similar *p* values. It is noted that immunoglobulin production by macrophages in the context of tumor microenvironments has been described [42] - it is not discussed as it does not appear relevant to the topic of our report. Additional pathway analysis data are shown in Supplementary Fig. S9.

We next investigated the effects of pRCC on storage lipid accumulation in macrophages in vitro by exposing human macrophages to cell-free conditioned medium from cultures of the ACHN cell line, which is derived from a human renal cell carcinoma and exhibits pRCC features [26]. Exposure to ACHN-conditioned medium induced macrophage production of chemokines and cytokines, including IL-8, IL-6, CCL2, and CXCL16 (Fig. S8A), which are characteristic of the response of tumor-associated macrophages to factors released by cancer cells [27–29]. ACHN-medium-treated macrophages also exhibited lipid droplet accumulation (Fig. 4AB), in agreement with previous observations [14], indicating the presence in the medium of lipogenic bioproduct(s) of the ACHN cells. Since IL-8 produced by cultured pRCC cells has been proposed as a foam-cell-inducing factor [14], we investigated the relationship among pRCC, foam cell formation, and IL-8 in our experimental system. We found that IL-8 is lipogenic for macrophages, but at a concentration ~10-fold higher than that measured in the ACHN-conditioned medium (~0.5ng/ml) (Fig. S8BC and Table S4). Thus, the lipogenic effect of the ACHN medium is unlikely due to IL-8 produced by the tumor cells. We cannot exclude, however, that IL-8 is lipogenic via paracrine-autocrine mechanisms in the tumor microenvironment in vivo since, for example, ACHN-medium-treated macrophages produce lipogenic concentrations of this cytokine (~5ng/ml) (Fig. S8A and Table S4).

Storage lipid analysis by enzymatic assays showed that lipid droplet accumulation in ACHN-medium-treated macrophages correlated with increased levels of both TAG and cholesterol (Fig. 4C). Moreover, the lipid droplet content of these macrophages decreased upon treatment with a chemical inhibitor of acyl-coenzyme A:cholesterol acyltransferase (ACAT), the enzyme that converts cholesterol to cholesteryl esters, and, to some extent, also with a DGAT inhibitor (Fig. 4D). These results suggest yet another context-specific mechanism of foam cell formation. Indeed, metabolism-related gene expression analysis identified mechanisms for both TAG and cholesterol accumulation. Pathways related to glycolysis featured among the top ranked Gene Ontology (GO) annotations related to metabolic processes (Fig. 4E). At the gene-level, the top four upregulated metabolic genes encoded glycolytic enzymes (Table S5, and Supplementary text). We also found gene markers of reduced TCA cycle, including upregulation of adenylate kinase 4 (AK4), a key metabolic regulator increasing glycolysis and inhibiting TCA cycle and oxidative phosphorylation [30], and downregulation of PPARGC1A (Table S5). The latter gene encodes PGC-1α, a master regulator of energy metabolism that promotes fatty acid oxidation and TCA cycle and decreases TAG storage [31] (Table S5 and Supplementary text). Together, increased glycolysis and reduced TCA cycle would result in routing of pyruvate towards de novo lipogenesis.

Among additional markers of TAG accumulation is notable the increased expression of genes associated with or regulated by YAP/TAZ signaling, which regulates metastasis and metabolic reprogramming, including lipid metabolism, in cancer cells [32]. These genes include TEAD transcription factors, the perilipin PLIN5, and the fructose transporter SLC2A5 (increased fructose uptake may lead to lipogenesis via fructolysis) (Table S5 and Supplementary text). Increased YAP/TAZ signaling is also supported by the above-mentioned upregulation of AK4 and downregulation of phospholipase D family member 6 (PLD6), since both gene expression changes might result in decreased activity of AMPK, which inhibits YAP/TAZ [33] (Table S5 and Supplementary text). Gene expression markers of impaired fatty acid oxidation and additional mechanisms of lipid accumulation in ACHN-medium-treated macrophages are discussed in the Supplementary text.

The ACHN-medium-treated macrophages also exhibited gene expression changes associated with dysregulation of cholesterol metabolism. For example, the scavenger receptor CD36, which is a key regulator of cholesterol homeostasis, was downregulated, presumably as a consequence of PPARGC1A downregulation [31] (Table S5). CD36 induces cholesterol depletion by promoting macrophage cholesterol efflux and proteasomal degradation of HMG-CoA reductase, the rate-limiting enzyme in sterol synthesis [34]. An additional marker of dysregulated cholesterol homeostasis is the downregulation of adenylate cyclase (ADCY1), which generates cAMP signaling for cholesterol efflux in atherogenic foam cells [35]. The above-proposed increased YAP/TAZ signaling might also result in cholesterol accumulation, since YAP/TAZ is involved in the metabolism of fatty acids and sterols [32].

Collectively, gene expression data point toward macrophage metabolic reprogramming in response to factors in the ACHN-conditioned medium. This reprogramming includes increased glycolysis, impaired TCA cycle and oxidative metabolism, decreased lipolysis, and dysregulated cholesterol homeostasis.

## Discussion

The data reported above show that the mechanisms of foam cell biogenesis differ with disease context. That is the case regardless of the chemical nature of the storage lipids they accumulate. For example, macrophages infected with *M. tuberculosis* and *C. neoformans* are enriched in TAG, as demonstrated by the drastic lipid droplet decrease caused by pharmacological inhibition of TAG biosynthesis in the two infections. In both cases, the accumulation of TAG likely results from a switch from oxidative to glycolytic metabolism that includes increased biosynthesis and decreased catabolism of lipids. However, the molecular mechanisms underlying the metabolic reprogramming of macrophages differ between the two infections, as indicated by the different effect of rapamycin on lipid droplet accumulation in the two infections. Still other mechanisms are likely at play in pRCC-associated macrophages, which accumulate neutral lipids by reprogramming both cholesterol and TAG metabolism. Moreover, although gene expression levels do not directly translate into protein levels, protein activity, and metabolic fluxes, the gene expression data presented above clearly imply that the metabolic remodeling leading to neutral lipid accumulation occurs through signaling, regulatory, and effector mechanisms that are specific to each experimental condition. Therefore, our data show that macrophage foam cells in different diseases are mechanistically heterogeneous even though they may be similar histochemically (lipid droplets consistently confer a “pale bubbly” appearance upon H&E staining) and perhaps even functionally, as discussed below.

A key to understanding foam cell heterogeneity is the biochemical diversity of the microenvironments driving their biogenesis. It is well established that uptake of exogenous lipids can drive foam cell formation. This is the case in atherogenesis, where sequestration of cholesterol-rich lipoproteins in the arterial wall leads to endothelial activation, recruitment of monocytes, and monocyte differentiation into lipoprotein-ingesting phagocytes that become foam cells [3]. Moreover, in some cancers, such as colon cancer, the fatty acid-enriched environment induces lipid droplet accumulation in tumor-associated macrophages [15]. It would be fallacious, however, to associate foam cell formation exclusively with exogenous lipid uptake. Instead, our work points to additional scenarios in which various microenvironment-specific signals trigger a metabolic remodeling of local macrophages that leads to excess intracellular fatty acids that are detoxified by storage in droplets. These environmental triggers may include pathogen-derived molecules, cancer cell products, inflammatory signals from activated and/or damaged cells that, for example, activate Toll-like receptors (TLR) (or other pattern recognition receptors) resulting in the reshaping of the macrophage lipidome [36]. Additional heterogeneity may result from the combinatorial effects of multiple foam-cell-inducing signals in some microenvironments. For example, foam cells may be induced in pRCC by yet unidentified cancer cell products, as indicated by our results with ACHN-conditioned medium, together with lipogenic proinflammatory cytokines, such as IL-8, produced by tumor-associated-macrophages. Moreover, additional mediators might be produced by cell types that are not represented in our in vitro system, such as the lipid-filled tumor cells that we observed in the pRCC bioptic tissue. In tuberculosis, we showed that, in addition to bacterial components that presumably trigger TLR2 signaling [37, 38], macrophage lipid accumulation requires another lipogenic proinflammatory cytokine, TNFα, produced by infected macrophages [5]. In cryptococcosis, the lung epithelial cells secrete IL-8 [11], suggesting a possible role of this cytokine in cryptococcal foam cell formation. Additional work is needed to identify the exogenous (i.e., generated by microbes, cancer cells, or other cell types) trigger signals and to determine how commonly foam cells are induced by combinations of exogenous and autocrine/paracrine signals.

It is reasonable to assume that, despite the different mechanisms of biogenesis, the presence of foam cells represents a maladaptive immune response in all the pathological contexts they form. Generally, lipid-laden macrophages tend to lose protective immune functions, including phagocytosis, efferocytosis, and autophagy. They can also induce tissue damage, contribute to necrosis, exhibit impaired antimicrobial activity, and even sustain survival of intracellular pathogens (reviewed in [1]). Indeed, given their contribution to pathogenesis, foam cells have been recognized as targets of pharmacological intervention, for example in atherosclerosis and some cancers [15, 39]. Moreover, foam cells are often associated with kidney disease, such as focal and segmental glomerulosclerosis and diabetic nephropathy [40], in addition to the pRCC investigated in the present work. Their pathophysiological significance in the kidney remains puzzling, and all mechanistic hypotheses on their biogenesis derive from the atherosclerosis literature [40]. Recognizing that foam cells do not result only from macrophage uptake of exogenous lipids and that the mechanisms of their formation are microenvironment-specific opens new directions for mechanistic and drug development research of high biomedical significance.

## Materials and Methods

### Cell cultures.

Peripheral blood mononuclear cells (PBMC) were isolated and monocyte-derived macrophages (MDM) were generated as previously described [5]. Briefly, human blood was obtained from the New York Blood Center (Long Island City, NY, USA), and PBMC were isolated by Ficoll density gradient centrifugation (Ficoll-Paque, GE Healthcare, Uppsala, Sweden). Isolated PBMC were washed and resuspended in serum-free RPMI-1640 medium (Corning, Manassas, VA, USA) supplemented with 4 mM L-glutamine (Corning, Manassas, VA, USA) and seeded at a density of 1 × 10^7^ cells/ml in tissue culture multiwell plates or flasks. After 4 hr, non-adherent cells were removed by washing five times with 1× PBS (Corning, Manassas, VA, USA) and the adherent fraction was cultured over 7 days in complete RPMI medium (RPMI-1640 supplemented with 10% fetal bovine serum (Seradigm, Radnor, PA, USA) and 4 mM L-glutamine.

The ACHN cell line was obtained from the American Type Culture Collection (ATCC). To obtain conditioned medium, cells were grown in complete RPMI for 6–9 passages in humidified atmospheric air containing 5% CO_2_ at 37°C. Cell culture medium was collected 24 hr after the last passage, filtered through a 0.4 μM filter to remove any cell debris, and stored in aliquots at −80°C.

### Pathogens.

mCherry-expressing *M*. *tuberculosis* H_37_Rv strain was grown in liquid medium to mid-log phase and frozen stocks prepared for macrophage infection as described (5). mCherry-expressing *C. neoformans* H99 strain was grown in YPD medium overnight at 30°C with constant agitation, washed three times with 1× PBS, and resuspended in 1× PBS before use in macrophage infection. The final fungal concentration was adjusted to 1 × 10^8^ cell/ml with complete RPMI medium [RPMI-1640 supplemented with 10% heat-inactivated fetal bovine serum (Seradigm, Radnor, PA, USA) and 4 mM L-glutamine]. A suspension of heat-killed *C. neoformans* was prepared by resuspending the fungal cells in 1× PBS and incubating the suspension at 75°C for 1 hr in a heat block. To prepare cell-free culture supernatant, *C. neoformans* H99 was grown in complete RPMI medium for 24 hr (end of logarithmic growth phase), filtered through a 0.2 μM filter, and used for macrophage treatments.

### Macrophage treatments.

For infection experiments, following 7 days of MDM differentiation, MDM were counted and infected with either *M*. *tuberculosis or C. neoformans*. The pathogen inoculum for infection was prepared by adding the microorganisms to supplemented RPMI-1640 medium (as above) to obtain an MOI of 4 colony-forming units (cfu) per cell. Pathogen clumps were disrupted by vortexing with sterile 3-mm-diameter glass beads for 2 min; this suspension was used for MDM infection. MDM were incubated with *M*. *tuberculosis* for 24h. MDM were infected with *C. neoformans* for 3 h, washed with 1× PBS three times, and incubated with fresh medium for 24 hr. When chemical inhibitors were tested, they were added to the MDM at the time of infection. For treatment of uninfected MDMs with recombinant TNFα, the cytokine was incubated with the MDMs for 24 hr.

At 24 hr post-infection, supernatants were collected and frozen for cytokine analysis and MDM were washed with 1× PBS three times and collected for subsequent analysis. For treatment with ACHN conditioned medium or IL-8 recombinant cytokine (Abcam, Cambridge, England), after PBMC isolation, the adherent fraction of PBMC was treated with conditioned medium (50% fresh medium and 50% conditioned medium) or IL-8 for 7 days. Medium was replaced at 3 and 6 days of incubation. At day 7 post-treatment, supernatants were collected and frozen for cytokine analysis, and MDM were washed with 1× PBS three times and collected for subsequent analysis.

Cytokine or chemical inhibitor doses were selected based on available EC_50_ data and toxicity profiles with untreated macrophages (Trypan Blue staining or MTS assay; CellTiter 96 Aqueous One Solution Cell Proliferation Assay Promega, Madison, WI, USA). Only inhibitor doses resulting in >90% cell viability were utilized. The following concentrations were used: 30–90 nM DGAT inhibitor A922500 (PubChem CID: 24768261) (Santa Cruz Biotechnology, Dallas, TX, USA), 0.4 nM rapamycin (mTORC1 inhibitor) (Selleckchem, Houston, TX, USA), 10 μM ACAT inhibitor (PubChem CID: 10019206) (Santa Cruz Biotechnology, Dallas, TX, USA), and 0.2–6 ng/ml recombinant IL-8 (Research and Diagnostic Systems, Minneapolis, MN, USA).

### Imaging flow cytometry.

Imaging flow cytometry of MDM was performed as previously described [5]. MDM were detached from tissue culture plates by incubating with 5 mM EDTA in 1× PBS pH 8 for 30 min followed by gentle scraping, washed once with 1× PBS, and fixed with 4% paraformaldehyde in 1× PBS for 45 min at room temperature. Cells were then washed with 1× PBS containing 0.1% bovine serum albumin (PBS-BSA), resuspended in 50 μl of PBS-BSA containing 5 μl of Fc receptor blocking solution, FcX (BioLegend, San Diego, CA), and incubated at room temperature for 5 min. After incubation, 50 μl of PBS-BSA containing 2.5 μl of CD11c APC antibody (clone S-HCL-3) or 5 μl of CD11c BV421 antibody (clone B-ly6 RUO) (BD Biosciences) were added to each tube, and samples were incubated at 4°C for 30 min. After washing with PBS-BSA, cells were stained with 0.3 μg/ml Bodipy 493/503 (Life Technologies, Carlsbad, CA) in 1× PBS for 15 min. For each experimental condition, data from 5,000–10,000 CD11c+ cells were acquired with an ImageStream^X^Mark II imaging flow cytometer (Amnis Corporation, Seattle, WA) using 60× magnification. Image data were analyzed by IDEAS software version 6.0 (Amnis Corporation, Seattle, WA) after applying a compensation matrix and selecting the region of interest (lipid droplets) with the Spot Mask tool. Median fluorescence intensity and spots per cell were extracted.

### Lipid quantification in macrophages.

MDM were detached from the cell culture plates as described above and transferred to microcentrifuge tubes. Cell pellets were obtained by culture centrifugation at 300*× g* and frozen for subsequent lipid measurement. Triglyceride-Glo^™^ Assay and Cholesterol/Cholesterol Ester-Glo^™^ Assay kits (Promega; Madison, WI, USA) were used to quantify triglycerides and cholesterol, following the manufacturer’s instructions. Cholesterol was quantified following addition of cholesteryl esterase to convert cholesteryl esters to free cholesterol, following manufacturer’s instructions.

### Mouse infection and tissue collection.

Female mice with an average weight of 20–25 g were used. C57BL/6 mice were purchased from the Jackson Laboratories. Animal studies were performed at the Public Health Research Institute Animal facility. All studies were conducted following biosafety level 2 (BSL-2) protocols and procedures approved by the Institutional Animal Care and Use Committee (IACUC) and Institutional Biosafety Committee of Rutgers University under protocol 999901066. *C. neoformans* wild type strain H99 was cultured on YPD medium. To prepare fungal cells for infection, an overnight culture of *C. neoformans* H99 was washed three times with 1× PBS and the concentration of yeast cells was determined by hemocytometer counting. The final fungal concentration was adjusted with PBS to 2 × 10^6^ cell/ml. Each mouse was infected intranasally with 1 × 10^5^ H99 cells in a 50 μl volume after being anesthetized with a mix of Ketamine (12.5 mg/mL) and Xylazine (1 mg/mL). After infection, animals were weighed daily and monitored twice daily for progression of disease, including weight loss, gait changes, labored breathing, and fur ruffling. Infected animals were sacrificed at day 7 post-infection, according to the experimental design and the Rutgers University IACUC approved animal protocol. A part of the dissected lung tissues was fixed in 10% formalin solution and sent to the Rutgers histopathological core facility for section preparation and staining with hematoxylin and eosin (H&E) using standard protocols. Another portion of the lung tissues was placed on labeled plastic cryohistology trays and frozen on dry ice for 2 minutes in a Styrofoam container. The samples were then wrapped with aluminum foil and stored in labeled Ziploc bags at −80°C until use for MALDI imaging.

### Human Biopsies.

Papillary renal cell carcinoma tissue was obtained from the Rutgers CINJ Biorepository. Tissue collected during surgery was frozen by the Biorepository using the PrestoCHILL machine, which allows for ultrafast freezing. All tissues analyzed derived from radical nephrectomies; the RWJUH surgical pathology reports indicated papillary renal cell carcinoma, type 1, WHO grade 2. Utilization of archived de-identified biospecimens from the Rutgers Cancer Institute of New Jersey Biospecimen Repository Services (BRS) Shared Resources was performed under IRB protocols 001006 and 002002.

### Matrix-assisted laser desorption/ionization (MALDI) mass spectrometry.

Frozen lung samples were used to prepare 12 μm-thick cryosections using a Leica CM1860 cryostat. Tissue sections were mounted onto indium tin oxide coated glass slides (Delta Technologies Limited, Loveland, Colorado) and stored individually in Ziploc bags at −80°C. Adjacent sections were prepared on Superfrost Plus^™^ glass microscope slides (Fisherbrand) for H&E staining, which was performed using standard protocols at the UTMB Histology core. Sections for MALDI imaging were coated with 2′,4′,6′-Trihydroxyacetophenone monohydrate at 10 mg/ml in 50:50 cyclohexane/methanol using a HTX TM Sprayer. 20 passes were performed over each tissue at a spray volume of 50 ml/min and nozzle temperature of 50°C. Once sprayed, samples were placed individually in Ziploc bags and immediately transferred to a −80°C freezer for storage. Following MALDI-MS image acquisition, matrix was removed from the slides by serial rinses in 50% ethanol, and tissue sections were allowed to airdry at room temperature for 30 min. The tissue sections were then transferred to the UTMB histology core for H&E and Oil Red staining using standard protocols.

MALDI MS imaging was performed using a Q-Exactive HF mass spectrometer (Thermo Scientific, Bremen, DE) fitted with a MALDI/ESI Injector (Spectroglyph LLC, Kennewick, WA). Laser post-ionization (MALDI-2) was used to enhance analytical sensitivity for triglycerides and cholesteryl esters. Images were acquired at 20 micrometer voxel size, using a pulse energy of ~6 mJ and repetition rate of 30Hz. Q Exactive HF MS Scan parameters were optimized for triglyceride and cholesteryl ester detection: polarity – positive, scan range – 350–1500 m/z, resolution – 120,000, automatic gain control - off, maximum inject time 250 ms. ImageInsight^™^ (Spectroglyph LLC) software was used for initial data visualization and to convert data files into imzML format for visualization and further processing in SCiLS^™^ software (Bruker, Billerica, MA). TAG and CE species were predominantly detected as potassium adducts and lipid identifications were assigned within a 3pm mass tolerance range using Lipid Maps database. All lipid images produced were normalized to the total ion chromatogram.

### Cytokine measurement.

A panel of cytokines/chemokines/growth factors was measured using Luminex technology at the Rutgers Immune Monitoring and Advanced Genomics Core Facility. Assays were performed in microtiter plates and plates were read with the Luminex 200 instrument. Each sample was measured in duplicate.

### Transcriptomics and pathway analysis.

RNA was isolated by using TRI reagent (Molecular Research Center, Cincinnati, OH, USA) according to manufacturer’s instructions. RNA-Seq was conducted at the Rutgers Genomics Center. The quality of RNA was first assessed for integrity on an Agilent TapeStation using high sensitivity RNA kit, PN-5067–5579 (Agilent technologies Inc, CA). Samples with RNA integrity number (RIN) >7.0 were considered to have sufficient quality for subsequent processing. Illumina compatible cDNA libraries generated for polyA selected mRNA using NEB next ultra RNA library preparation kit Cat#E7530L (New England Biolabs Inc, MA). The cDNA libraries were purified using AmpureXP beads, Product No: A63882 (Beckman Coulter) and analyzed on an Agilent TapeStation using HS D1000 screen tape PN- 5067–5584 (Agilent Technologies Inc, CA) to estimate the size of the library and quantitated using Qubit 4 Fluorometer, using HS reagent kit, Cat. No. Q33231 (Thermofisher Scientific, MA). Equimolar amounts of barcoded libraries pooled together and sequenced on Illumina NovaSeq 6000 Instrument (Illumina, San Diego, CA) using SP flow cells kits (cat # 20040326) with 2×150 cycles configuration.

Read count and gene-level transcript abundance analysis were performed by the Rutgers Molecular and Genomics Informatics Core. In brief, raw transcriptome reads were assessed for quality control (FASTQC v0.11.8) and trimmed for quality and adapter contaminant (cutadapt v 2.5). Trimmed reads were aligned to the mouse genome (GRCh37) using STAR (v2.6.1), followed by transcript abundance calculation and hit count extraction with StringTie (v2.0) and featureCounts (v1.6.4), respectively. The differential expression between sample classes (treated vs non treated for each condition) was tested with coincident extreme ranks in numerical observations (CERNO) [41] for metabolism-related Gene Ontology gene sets. The Benjamini–Hochberg method was used to calculate the false discovery rate (FDR). Gene sets (pathways) were identified at a cutoff false discovery rate of 0.05. Genes in differentially expressed metabolism-related Gene Ontology gene sets having FDR < 0.05 were selected based on individual *p* value (*p* << 0.05) and ranked by log2 fold change (treated vs non-treated in each condition) and number of occurrences in the differentially expressed gene sets. The top five down- and up-regulated genes were selected for further analysis. Differentially expressed genes (*p* < 0.05) reported in the published literature as functionally related to the top five up- and down-regulated genes in each condition were also selected for analysis and data interpretation.

## Supplementary Material

Supplement 1

## Abbreviations:

CE: cholesteryl esters
H&E: hematoxylin and eosin
MALDI: matrix-assisted laser desorption/ionization mass spectrometry
MDM: monocyte-derived macrophages
pRCC: papillary renal cell carcinoma
TAG: triglycerides
