# Supplementary material for: Heterogeneity of foam cell biogenesis across diseases
Source: bioRxiv. 2024 Apr 20:2023.06.08.542766. Originally published 2023 Jun 8. Preprint. [Version 3] doi: 10.1101/2023.06.08.542766 (PMC10274805; doi:10.1101/2023.06.08.542766)
Supplement: Supplementary file 1 [file NIHPP2023.06.08.542766v3-supplement-1.pdf]

bioRxiv preprint doi: https://doi.org/10.1101/2023.06.08.542766; this version posted April 20, 2024. The copyright holder for this preprint (which was not certified by peer review) is the author/funder, who has granted bioRxiv a license to display the preprint in perpetuity. It is made available under aCC-BY 4.0 International license.

Supplementary Information

Heterogeneity of foam cell biogenesis across diseases

Valentina Guerrini[1*], Brendan Prideaux[2], Rehan Khan[1], Selvakumar Subbian[1], Yina Wang[3], Evita Sadimin[4*], Siddhi Pawar[3], Rahul Ukey[1], Eric A. Singer[4*], Chaoyang Xue[3], Maria Laura Gennaro[1**]

Supplementary Figures

Fig. S1. Low-magnification images of murine lungs uninfected and infected with *C. neoformans* H99.

Fig. S2. Hematoxylin-eosin staining and MALDI imaging of uninfected murine lung sections.

Fig. S3. Macrophage lipid droplet accumulation in response to infection with live *C. neoformans*, or treatment with cell-free fungal culture medium, or with heat-killed fungal cells.

Fig. S4. Metabolism-associated pathways (A) upregulated in *M. tuberculosis*-infected human monocyte-derived macrophages and (B) downregulated in *C. neoformans*-infected human monocyte-derived macrophages.

Fig. S5. Spatial distributions of cholesterol ester (CE) species in papillary renal cell carcinoma (pRCC) (Patient P1).

Fig. S6. Spatial distribution of triglyceride (TAG) species in papillary renal cell carcinoma (pRCC) (Patient P1).

Fig. S7. Spatial distribution of triglyceride (TAG) and cholesteryl ester (CE) species in papillary renal cell carcinoma biopsies collected from patients P2 and P3**.**

Fig. S8. Production of cytokines by human monocyte-derived macrophages treated with ACHN-conditioned culture medium and lipogenic effect of IL-8 at different concentrations.

Fig. S9. Metabolism-associated pathways downregulated in ACHN-medium-treated human monocyte-derived macrophages.

Supplementary Tables

Table S1. List of triglyceride and cholesteryl ester species detected in tissues by MALDI-MSI.

Table S2a. Top five down- and up-regulated genes in macrophages infected with *M. tuberculosis.*

bioRxiv preprint doi: https://doi.org/10.1101/2023.06.08.542766; this version posted April 20, 2024. The copyright holder for this preprint (which was not certified by peer review) is the author/funder, who has granted bioRxiv a license to display the preprint in perpetuity. It is made available under aCC-BY 4.0 International license.

Table S2b. Genes functionally related to top five down- and up-regulated genes in macrophages infected with *M. tuberculosis.*

Table S3a. Top five down- and up-regulated genes in macrophages infected with *C. neoformans.*

Table S3b. Genes functionally related to top five down- and up-regulated genes in macrophages infected with *C. neoformans.*

Table S4. Levels of cytokines in supernatants of ACHN cell cultures and MDM treated with ACHN-conditioned culture medium.

Table S5a. Top five down- and up-regulated genes in macrophages treated with ACHN-conditioned medium.

Table S5b. Genes functionally related to top five down- and up-regulated genes in macrophages treated with ACHN-conditioned culture medium.

Supplementary Text on transcriptomics analyses

Supplementary References

bioRxiv preprint doi: https://doi.org/10.1101/2023.06.08.542766; this version posted April 20, 2024. The copyright holder for this preprint (which was not certified by peer review) is the author/funder, who has granted bioRxiv a license to display the preprint in perpetuity. It is made available under aCC-BY 4.0 International license.

Supplementary Figures

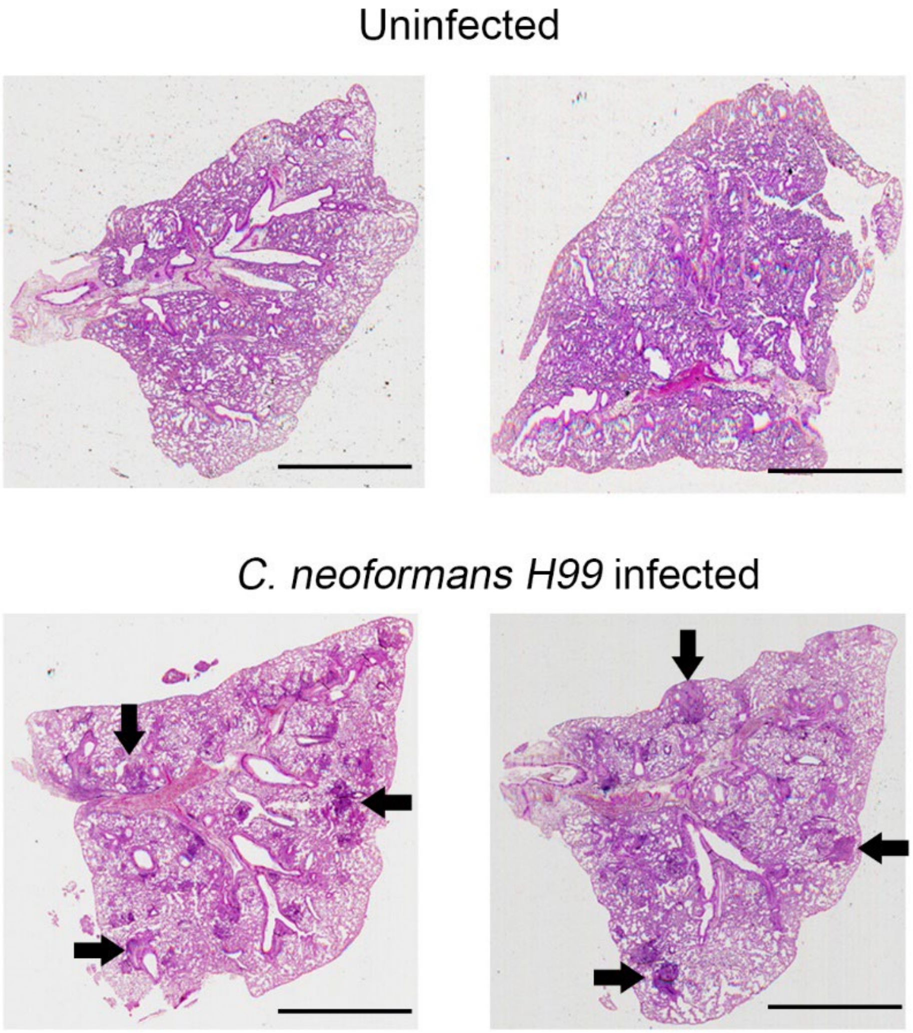

Supplementary Fig. S1. Low-magnification images of murine lungs uninfected and infected with *C. neoformans* H99. H&E staining of uninfected (top panels) and infected (bottom panels) lung sections. Black arrows show cellular aggregates in the infected tissue. Images were photographed at 20x magnification; scale bar is 2 mm.

bioRxiv preprint doi: https://doi.org/10.1101/2023.06.08.542766; this version posted April 20, 2024. The copyright holder for this preprint (which was not certified by peer review) is the author/funder, who has granted bioRxiv a license to display the preprint in perpetuity. It is made available under aCC-BY 4.0 International license.

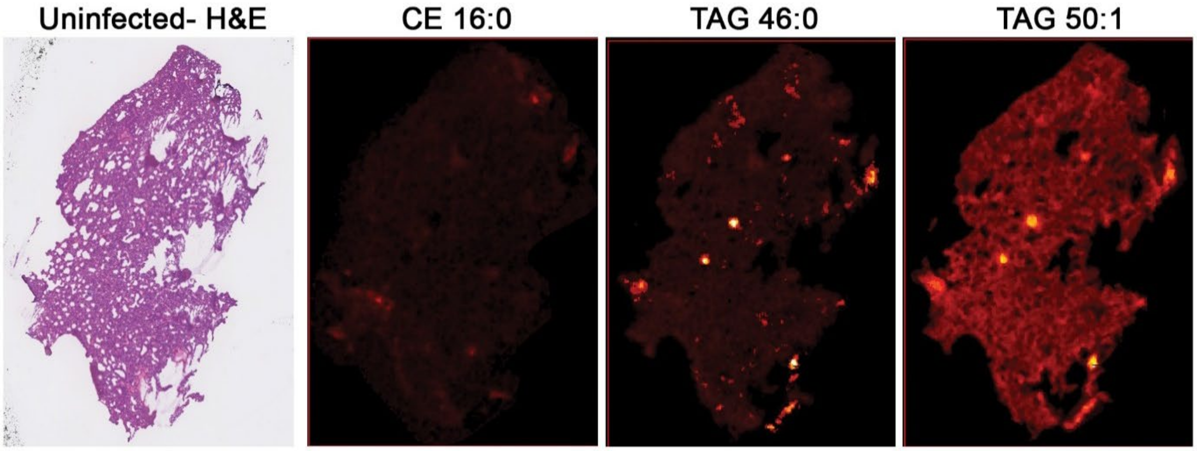

Supplementary Fig. S2. Hematoxylin-eosin staining and MALDI imaging of uninfected murine lung sections. The left panel shows H&E staining of uninfected lung sections. The three additional panels show MALDI imaging of storage lipids in lung sections contiguous to those used for H&E staining. Representative species are shown, CE (16:0) and TAG (46:0 and 50:1), corresponding to those shown in Fig. 1C (TAG and CE species detected are listed in **Table S1**).

bioRxiv preprint doi: https://doi.org/10.1101/2023.06.08.542766; this version posted April 20, 2024. The copyright holder for this preprint (which was not certified by peer review) is the author/funder, who has granted bioRxiv a license to display the preprint in perpetuity. It is made available under aCC-BY 4.0 International license.

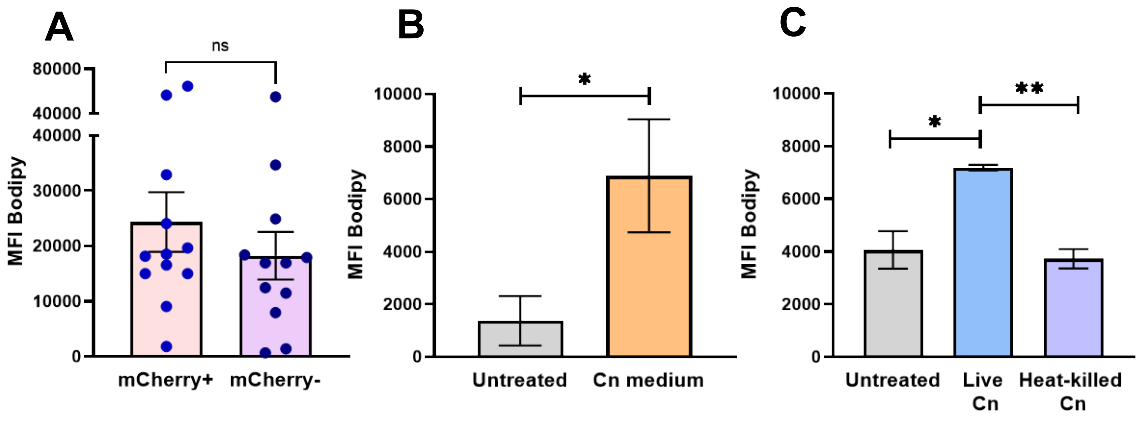

Supplementary Fig. S3. Macrophage lipid droplet accumulation in response to infection with live *C. neoformans*, or treatment with cell-free fungal culture medium, or with heat-killed fungal cells. In all panels, lipid droplet content of monocyte-derived macrophages (MDM) was measured by imaging flow cytometry and expressed as median fluorescence intensity (MFI) of Bodipy 493/503. **A.** MDM were infected with *C. neoformans* expressing mCherry and lipid droplet content was measured at 24 h post-infection in macrophages in the infected culture wells carrying or not carrying intracellular fungi (mCherry+ and mCherry-). Each dot represents one human donor. **B.** MDM were treated for 7 days with cell-free culture filtrate of *C. neoformans* grown in RPMI medium (Cn medium) or sterile RPMI medium (untreated). **C.** THP1 cells were treated with live or heat-killed *C. neoformans* (Cn) for 24 h. Statistics: mean and SEM (A-B) and mean and SD of triplicates (C-D) are shown; ns, non-significant; *, p <0.05; **, p <0.01 (paired t-test).

bioRxiv preprint doi: https://doi.org/10.1101/2023.06.08.542766; this version posted April 20, 2024. The copyright holder for this preprint (which was not certified by peer review) is the author/funder, who has granted bioRxiv a license to display the preprint in perpetuity. It is made available under aCC-BY 4.0 International license.

A. ***M. tuberculosis***

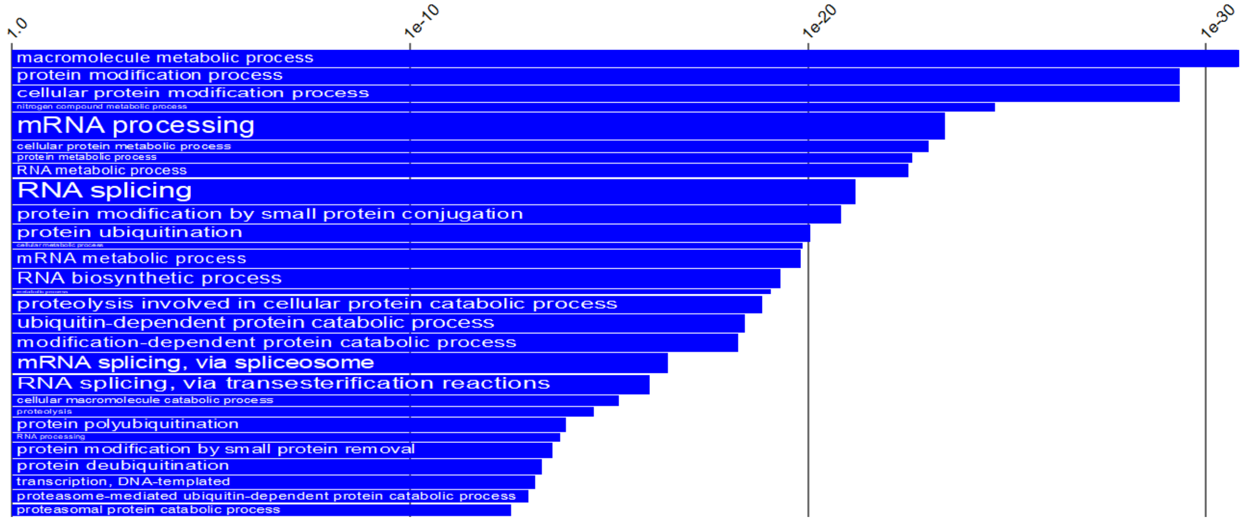

B. ***C. neoformans***

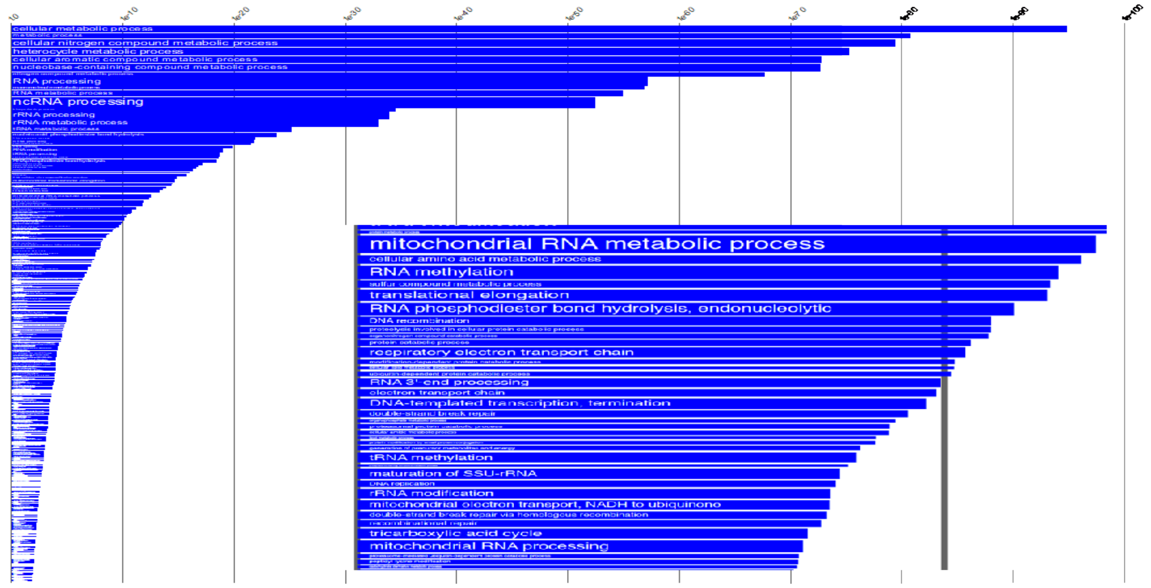

Supplementary Fig. S4. A. Upregulated metabolism-associated pathways in *M. tuberculosis*-infected human monocyte-derived macrophages. B. Downregulated metabolism-associated pathways in *C. neoformans*-infected human monocyte-derived macrophages. The panels show Gene Ontology (GO) annotations related to metabolic processes that were upregulated in *M. tuberculosis*-infected MDM and downregulated in *C. neoformans*-infected MDM, relative to uninfected control cells. The differential expression between sample classes (infected vs uninfected) was tested with coincident extreme ranks in numerical observations (CERNO). Pathways were selected using a cutoff false discovery rate of

bioRxiv preprint doi: https://doi.org/10.1101/2023.06.08.542766; this version posted April 20, 2024. The copyright holder for this preprint (which was not certified by peer review) is the author/funder, who has granted bioRxiv a license to display the preprint in perpetuity. It is made available under aCC-BY 4.0 International license.

0.05; the *p*-values for these pathways were plotted on the *x*-axis. To represent effect size, pathway gene sets containing fewer genes were given greater bar height/font size than were larger sets that yielded similar *p* values. For visualization purposes, panel A shows top-ranking annotations, and panel B. contains an inset highlighting mitochondria-related annotations.

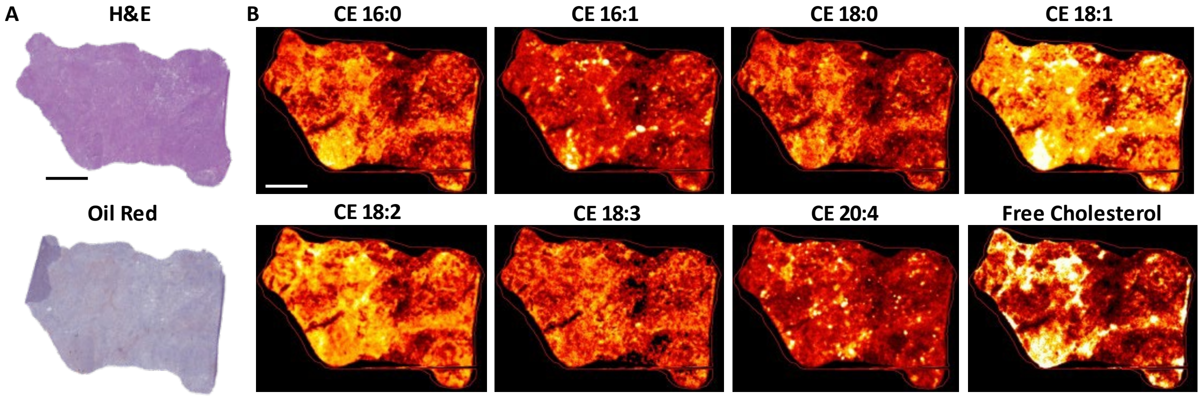

Supplementary Fig. S5. Spatial distribution of cholesterol ester (CE) species in papillary renal cell carcinoma (pRCC) (patient P1). A. Hematoxylin-eosin- and oil-red-stained kidney sections contiguous to those used for MALDI imaging. **B.** MALDI-2-MSI signal distribution for the major detected cholesterol ester species. Scale bar = 5 mm

bioRxiv preprint doi: https://doi.org/10.1101/2023.06.08.542766; this version posted April 20, 2024. The copyright holder for this preprint (which was not certified by peer review) is the author/funder, who has granted bioRxiv a license to display the preprint in perpetuity. It is made available under aCC-BY 4.0 International license.

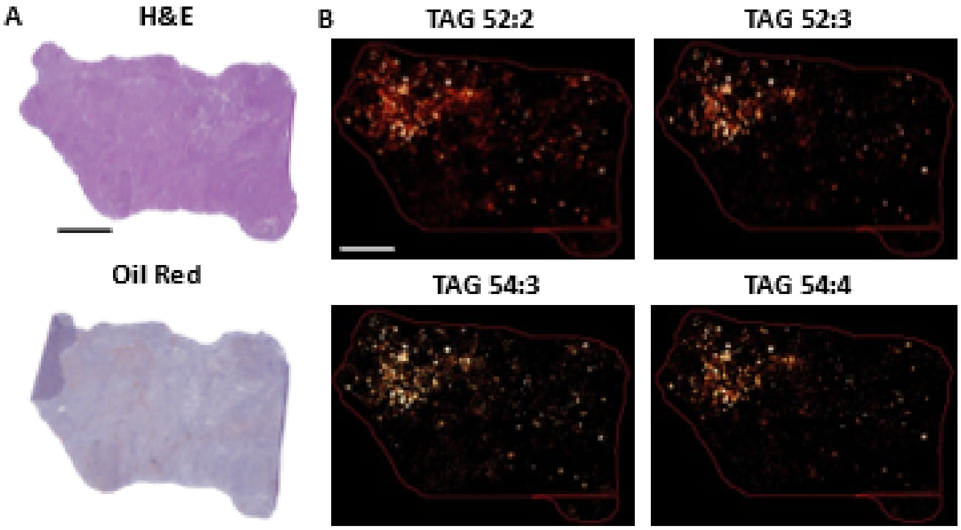

Supplementary Fig. S6. Spatial distribution of triglyceride (TAG) species in papillary renal cell carcinoma (pRCC) (patient P1). A. Hematoxylin-eosin- and oil-red-stained kidney sections contiguous to those used for MALDI imaging. **B.** MALDI-2-MSI signal distribution for four of the major detected TAG species. Scale bar = 5 mm

bioRxiv preprint doi: https://doi.org/10.1101/2023.06.08.542766; this version posted April 20, 2024. The copyright holder for this preprint (which was not certified by peer review) is the author/funder, who has granted bioRxiv a license to display the preprint in perpetuity. It is made available under aCC-BY 4.0 International license.

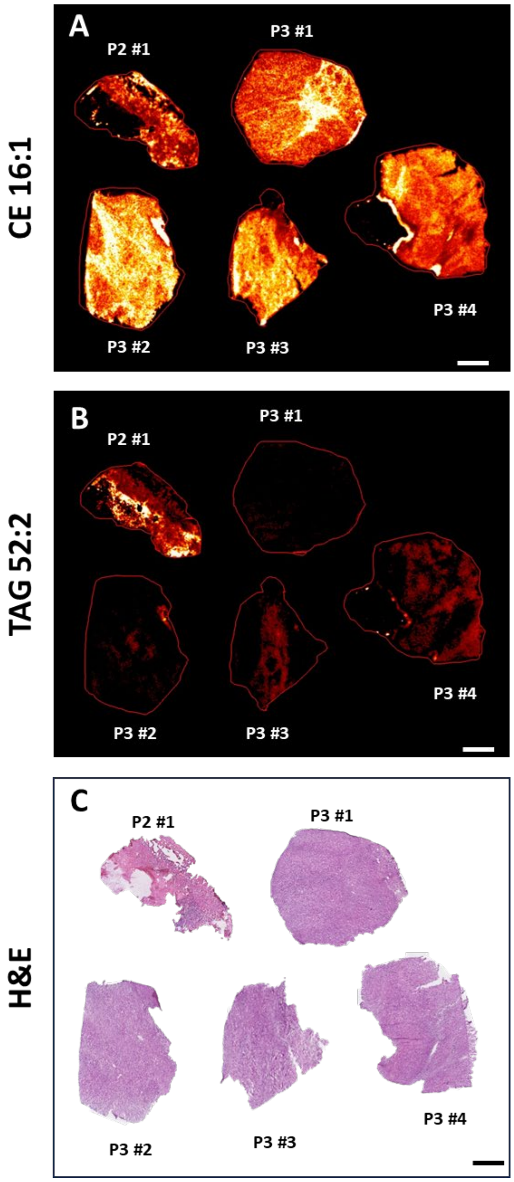

Supplementary Fig. S7 Spatial distribution of triglyceride (TAG) and cholesteryl ester (CE) species in papillary renal cell carcinoma biopsies collected from patients P2 and P3. A. MALDI-2-MSI signal distribution for CE 16:1. **B.** MALDI-2-MSI signal distribution for TAG 52:2. C. Hematoxylin-eosin-stained kidney sections contiguous to those used for MALDI imaging. P = patient ID, # = biopsy number. Scale bar = 5 mm

bioRxiv preprint doi: https://doi.org/10.1101/2023.06.08.542766; this version posted April 20, 2024. The copyright holder for this preprint (which was not certified by peer review) is the author/funder, who has granted bioRxiv a license to display the preprint in perpetuity. It is made available under aCC-BY 4.0 International license.

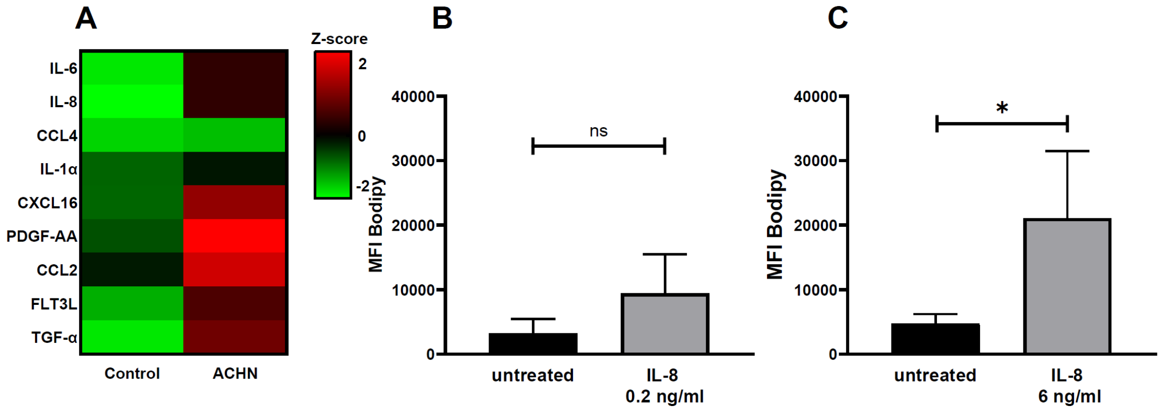

Supplementary Fig. S8. Production of cytokines by human monocyte-derived macrophages treated with ACHN-conditioned culture medium and lipogenic effect of IL-8 at different concentrations. A. The heat map shows cytokines and chemokines differentially expressed by MDM exposed to conditioned medium from ACHN cell cultures relative to control, untreated MDM, as described in Fig. 4. Cytokines/chemokines were measured as technical duplicates using a 43-plex array and Luminex technology. The average *Z*-score for five donors was calculated for each analyte. Color gradient from green to red represents increasing concentrations. Data shown had $p < 0.05$ (paired *t*-test). **B-C.** Lipid droplet content of MDM was measured as median fluorescence intensity (MFI) of Bodipy 493/503 by imaging flow cytometry, following 7-day treatment with recombinant IL-8, at two different concentrations, as indicated. Statistics: mean and SD of triplicate measurements (B) and mean and SEM of multiple donors (C) are shown; ns, non-significant; *, p <0.05 (B, unpaired *t*-test; C, Mann-Whitney test).

bioRxiv preprint doi: https://doi.org/10.1101/2023.06.08.542766; this version posted April 20, 2024. The copyright holder for this preprint (which was not certified by peer review) is the author/funder, who has granted bioRxiv a license to display the preprint in perpetuity. It is made available under aCC-BY 4.0 International license.

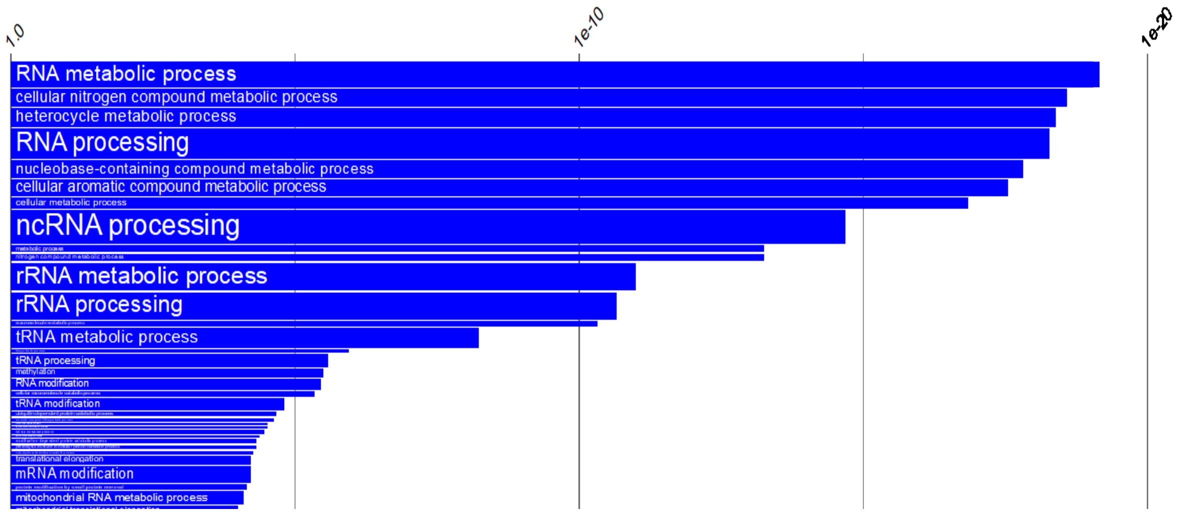

Supplementary Fig. S9. Downregulated metabolism-associated pathways in ACHN-medium-treated human monocyte-derived macrophages. The figure shows Gene Ontology (GO) annotations related to metabolic processes that were downregulated in MDM treated with ACHN conditioned medium, relative to untreated control cells. The differential expression between sample classes (treated vs untreated) was tested and selected pathways were represented as described in Figure S4. For visualization purposes, top-ranking annotations are shown.

bioRxiv preprint doi: https://doi.org/10.1101/2023.06.08.542766; this version posted April 20, 2024. The copyright holder for this preprint (which was not certified by peer review) is the author/funder, who has granted bioRxiv a license to display the preprint in perpetuity. It is made available under aCC-BY 4.0 International license.

Supplementary Tables

Supplementary Table S1. List of triglyceride and cholesteryl ester species detected in tissues by MALDI-MSI.

TAG	Detected in *Cryptococcus*	Detected in pRCC	CE	Detected in *Cryptococcus*	Detected in pRCC
Species	Tissue	Tissue	Species	Tissue	Tissue
48:0	✓	×	16:0	✓	✓
48:1	✓	×	16:1	✓	✓
48:2	✓	×	18:0	✓	✓
48:3	✓	×	18:1	✓	✓
50:0	✓	×	18:2	✓	✓
50:1	✓	✓	18:3	✓	✓
50:2	✓	✓	20:1	✓	✓
50:3	✓	✓	20:2	×	✓
50:4	✓	×	20:4	✓	✓
52:1	✓	×			
52:2	✓	✓			
52:3	✓	✓			
52:4	✓	✓			
52:5	✓	✓			
52:6	✓	×			
54:2	✓	✓			
54:3	✓	✓			
54:4	✓	✓			
54:5	✓	✓			
54:6	✓	×			
54:7	✓	×			
56:3	✓	×			
56:4	✓	×			

Triglyceride (TAG) species (identified by the total number of carbons and double bonds in the esterified fatty acids) and cholesteryl ester (CE) species detected by MALDI-MSI in the cryptococcus-infected lung tissues and papillary renal cell carcinoma (pRCC) tissue. ✓, detected; x, not detected.

bioRxiv preprint doi: https://doi.org/10.1101/2023.06.08.542766; this version posted April 20, 2024. The copyright holder for this preprint (which was not certified by peer review) is the author/funder, who has granted bioRxiv a license to display the preprint in perpetuity. It is made available under aCC-BY 4.0 International license.

Supplementary Table S2a. Top five down- and up-regulated genes in macrophages infected with *M. tuberculosis.*

Gene ID	Gene	log2FoldChange	*p* value
ENSG00000005469	CROT	-0.694245982	6.81E-10
ENSG00000183549	ACSM5	-1.875070163	2.15E-12
ENSG00000072210	ALDH3A2	-0.675169083	1.18E-15
ENSG00000131238	PPT1	-0.769679647	6.47E-16
ENSG00000168765	GSTM4	-0.846851963	4.64E-14
ENSG00000105325	FZR1	0.451795	1.01E-10
ENSG00000186187	ZNRF1	0.884946	4.79E-17
ENSG00000108306	FBXL20	0.849817	9.11E-17
ENSG00000186591	UBE2H	0.795385	3.10E-25
ENSG00000187555	USP7	0.206178	4.81E-06

Genes in differentially expressed metabolism-related Gene Ontology gene sets having FDR < 0.05 were selected based on individual *p* value ($p << 0.05$) and ranked by log2 fold change (infected vs non-infected) and number of occurrences in the differentially expressed gene sets. The top five down- and up-regulated genes were selected for further analysis.

bioRxiv preprint doi: https://doi.org/10.1101/2023.06.08.542766; this version posted April 20, 2024. The copyright holder for this preprint (which was not certified by peer review) is the author/funder, who has granted bioRxiv a license to display the preprint in perpetuity. It is made available under aCC-BY 4.0 International license.

Supplementary Table S2b. Genes functionally related with the top five down- and up-regulated genes in macrophages infected with *M. tuberculosis*.

Gene ID	Gene	log2FoldChange	*p* value
ENSG00000215009	ACSM4	-1.65627	2.66E-05
ENSG00000130377	ACSBG2	-1.09841	3.99E-03
ENSG00000140284	SLC27A2	0.929524	2.40E-03
ENSG00000143554	SLC27A3	0.440446	2.69E-02
ENSG00000110090	CPT1A	-0.69747	6.60E-05
ENSG00000157184	CPT2	-0.32849	3.84E-05
ENSG00000154227	CERS3	1.207873	6.71E-03
ENSG00000139624	CERS5	0.437953	5.94E-06
ENSG00000177076	ACER2	-0.46598	2.77E-02
ENSG00000141510	TP53	-0.34037	1.19E-04
ENSG00000109670	FBXW7	0.514687	0.00000987
ENSG00000135679	MDM2	0.745793	1.91E-10
ENSG00000100219	XBP1	1.329735	1.36E-11
ENSG00000155792	DEPTOR	-2.04205	0.0000007
ENSG00000164181	ELOVL7	4.018986	1.04E-33
ENSG00000175445	LPL	-1.17544	6.57E-08
ENSG00000134184	GSTM1	-0.95459	0.000000069
ENSG00000213366	GSTM2	-1.23318	0.000000626
ENSG00000166851	PLK1	-1.07326	0.0025
ENSG00000092964	DPYSL2	-0.9294	1.38E-06
ENSG00000013588	GPRC5A	6.429513	2.92E-81
ENSG00000181773	GPR3	3.096538	1.07E-34
ENSG00000183484	GPR132	3.238435	2.91E-26
ENSG00000180758	GPR157	2.357273	5.3E-21
ENSG00000173698	GPR64	2.313555	8.38E-17
ENSG00000139572	GPR84	2.131323	4.78E-10
ENSG00000182885	GPR97	2.623284	1.95E-09
ENSG00000188316	ENO4	-1.32246	7.67E-07
ENSG00000077721	UBE2A	0.552901	1.40E-08
ENSG00000160087	UBE2J2	0.405355	2.54E-08
ENSG00000177889	UBE2N	0.348958	3.68E-08
ENSG00000131508	UBE2D2	0.331604	7.16E-07
ENSG00000185651	UBE2L3	0.381012	3.78E-06
ENSG00000160714	UBE2Q1	0.35768	2.01E-05
ENSG00000072401	UBE2D1	0.867935	3.27E-05

bioRxiv preprint doi: https://doi.org/10.1101/2023.06.08.542766; this version posted April 20, 2024. The copyright holder for this preprint (which was not certified by peer review) is the author/funder, who has granted bioRxiv a license to display the preprint in perpetuity. It is made available under aCC-BY 4.0 International license.

ENSG00000104343	UBE2W	0.503851	4.69E-05
ENSG00000182247	UBE2E2	0.746942	0.000065
ENSG00000078140	UBE2K	0.277765	0.000104
ENSG00000170142	UBE2E1	0.523719	0.000327
ENSG00000156587	UBE2L6	1.059927	0.000437
ENSG00000175931	UBE2O	0.349526	0.000465
ENSG00000130725	UBE2M	0.31568	0.000899
ENSG00000107341	UBE2R2	0.236068	0.002875
ENSG00000184182	UBE2F	0.245525	0.021078
ENSG00000198833	UBE2J1	0.305753	0.02493
ENSG00000187531	SIRT7	0.5201	1.33E-10
ENSG00000177463	NR2C2	0.771918	0.000254
ENSG00000182054	IDH2	-0.83851	2.92E-14
ENSG00000138413	IDH1	-0.94364	2.62E-18
ENSG00000142208	AKT1	-0.16012	0.045604
ENSG00000082701	GSK3B	0.515329	0.000158

Differentially expressed genes (p < 0.05) reported in the published literature as functionally related to the top five up- and down-regulated genes in Table S2a.

bioRxiv preprint doi: https://doi.org/10.1101/2023.06.08.542766; this version posted April 20, 2024. The copyright holder for this preprint (which was not certified by peer review) is the author/funder, who has granted bioRxiv a license to display the preprint in perpetuity. It is made available under aCC-BY 4.0 International license.

Supplementary Table S3a. Top five down- and up-regulated genes in macrophages infected with *C. neoformans.*

Gene	Gene Id	log2FoldChange	*p* value
ENSG00000116750	UCHL5	-0.20727	0.04472
ENSG00000138035	PNPT1	-0.54945	1.67E-10
ENSG00000130348	QRSL1	-0.37168	3.88E-07
ENSG00000196510	ANAPC7	-0.39341	0.000302
ENSG00000131467	PSME3	-0.30045	3.30E-05
ENSG00000109107	ALDOC	1.825971589	2.32E-10
ENSG00000111674	ENO2	1.489680497	9.70E-15
ENSG00000159399	HK2	1.121945578	6.74E-16
ENSG00000102144	PGK1	0.746990585	2.14E-08
ENSG00000111640	GAPDH	0.713009241	1.06E-07

Gene identification for gene-level analysis was performed as in Table S2a.

bioRxiv preprint doi: https://doi.org/10.1101/2023.06.08.542766; this version posted April 20, 2024. The copyright holder for this preprint (which was not certified by peer review) is the author/funder, who has granted bioRxiv a license to display the preprint in perpetuity. It is made available under aCC-BY 4.0 International license.

Supplementary Table S3b. Genes functionally related with the top five down- and up-regulated genes in macrophages infected with *C. neoformans.*

Gene	Gene Id	log2FoldChange	*p* value
ENSG00000185920	PTCH1	0.761480524	0.012629752
ENSG00000127528	KLF2	0.62022683	0.047195731
ENSG00000166819	PLIN1	0.973316711	0.003288899
ENSG00000147872	PLIN2	0.692199046	0.00059194
ENSG00000149925	ALDOA	0.568357915	4.63E-06
ENSG00000111669	TPI1	0.6108752	5.94E-07
ENSG00000171314	PGAM1	0.429844596	0.001449782
ENSG00000105220	GPI	0.684479515	7.26E-09
ENSG00000067057	PFKP	0.690877949	0.000281329
ENSG00000152256	PDK1	1.070412291	8.18E-08
ENSG00000067992	PDK3	0.363881857	0.004868894
ENSG00000134333	LDHA	1.050510273	3.90E-08
ENSG00000104812	GYS1	0.794960339	3.17E-07
ENSG00000174437	ATP2A2	-0.330898147	0.029025124
ENSG00000132356	PRKAA1	-0.186245418	0.045627492
ENSG00000131828	PDHA1	-0.222577362	0.025045384
ENSG00000072210	ALDH3A2	-0.331695382	7.63E-05
ENSG00000161618	ALDH16A1	-0.281990927	0.008872148
ENSG00000184254	ALDH1A3	-0.998071894	0.011142259
ENSG00000159423	ALDH4A1	-0.573208906	0.013428796
ENSG00000137124	ALDH1B1	-0.500558706	0.026687482
ENSG00000135218	CD36	-0.435604275	0.045176474

Differentially expressed genes ($p < 0.05$) reported in the published literature as functionally related to the top five up- and down-regulated genes in Table S3a.

bioRxiv preprint doi: https://doi.org/10.1101/2023.06.08.542766; this version posted April 20, 2024. The copyright holder for this preprint (which was not certified by peer review) is the author/funder, who has granted bioRxiv a license to display the preprint in perpetuity. It is made available under aCC-BY 4.0 International license.

Supplementary Table S4. Levels of cytokines in supernatants of ACHN cell cultures and MDM treated with ACHN-conditioned culture medium.

Cytokine	ACHN (pg/ml)	ACHN-medium treated MDM (pg/ml)
IL-6	174	82
IL-8	543	4889
CCL4	137	2414
IL-1α	4	34
CXCL16	23	1898
PDGF-AA	46	37
CCL2	405	13775
FLT3L	10	22
TGF-α	6	17

The level of cytokines produced by ACHN cell cultures was calculated as average of technical duplicates for each analyte. MDM from donors (n=5) was treated with ACHN-conditioned culture medium. The average level in MDM supernatants from the five donors was calculated for each analyte.

bioRxiv preprint doi: https://doi.org/10.1101/2023.06.08.542766; this version posted April 20, 2024. The copyright holder for this preprint (which was not certified by peer review) is the author/funder, who has granted bioRxiv a license to display the preprint in perpetuity. It is made available under aCC-BY 4.0 International license.

Supplementary Table S5a. Top five down and upregulated genes in macrophages treated with ACHN-conditioned culture medium.

Gene	Gene Id	log2FoldChange	*p* value
ENSG00000109819	PPARGC1A	-1.108179567	0.0019129
ENSG00000179598	PLD6	-0.340258537	0.0313289
ENSG00000164742	ADCY1	-1.789945859	1.59E-05
ENSG00000156239	N6AMT1	-0.407562926	0.0036691
ENSG00000215009	ACSM4	-0.732880759	0.0301056
ENSG00000111674	ENO2	0.585748	0.005669
ENSG00000160883	HK3	0.417557	0.035561
ENSG00000111669	TPI1	0.280168	0.035629
ENSG00000102144	PGK1	0.263218	0.041578
ENSG00000162433	AK4	0.550335	0.00025

Gene identification for gene-level analysis was performed as in Table S2a.

bioRxiv preprint doi: https://doi.org/10.1101/2023.06.08.542766; this version posted April 20, 2024. The copyright holder for this preprint (which was not certified by peer review) is the author/funder, who has granted bioRxiv a license to display the preprint in perpetuity. It is made available under aCC-BY 4.0 International license.

Supplementary Table S5b. Genes functionally related with the top five down and upregulated genes in macrophages treated with ACHN conditioned culture medium.

Gene	Gene Id	log2FoldChange	*p* value
ENSG00000135218	CD36	-0.71398	0.002177
ENSG00000166411	IDH3A	-0.36846	0.012774
ENSG00000110090	CPT1A	-0.3713	0.048744
ENSG00000165092	ALDH1A1	-0.6716	0.005934
ENSG00000214456	PLIN5	0.763578	0.033995
ENSG00000007866	TEAD3	0.442895	0.043061
ENSG00000142583	SLC2A5	0.948899	0.001353
ENSG00000114268	PFKFB4	0.682204	3.73E-05

Differentially expressed genes ($p < 0.05$) reported in the published literature as functionally related to the top five up- and down-regulated genes in Table S5a.

bioRxiv preprint doi: https://doi.org/10.1101/2023.06.08.542766; this version posted April 20, 2024. The copyright holder for this preprint (which was not certified by peer review) is the author/funder, who has granted bioRxiv a license to display the preprint in perpetuity. It is made available under aCC-BY 4.0 International license.

Supplementary text on transcriptomics analyses

1. Metabolism-related gene-level analysis of *M. tuberculosis*-infected monocyte-derived macrophages.

Genes in differentially expressed metabolism-related Gene Ontology gene sets having FDR < 0.05 were selected based on individual *p* value ($p << 0.05$) and ranked by log2 fold change (infected vs non-infected) and number of occurrences in the differentially expressed gene sets. We analyzed the top five down- and up-regulated genes (**Table S2a**) and differentially expressed genes ($p < 0.05$) that were functionally related to them (**Table S2b**). In the text below, and in the subsequent sections, the names of the top five up- and down-regulated genes (**Table S2a**) are shown in bold. The names of the up- and down-regulated functionally related genes (**Table S2b**) are underlined.

Multiple mechanisms of decreased fatty acid oxidation were identified. These included downregulation of genes involved in (i) synthesis of acyl-CoA (acyl-CoA synthases ACSM4, **ACSM5**, ACSBG2), (ii) acyl-CoA conversion to acyl-carnitine [(carnitine transferase in peroxisome (**CROT**) and mitochondria (CPT1A, CPT2)], which is required for transport across the mitochondrial membrane (*1-3*), and (iii) oxidation to fatty acids of fatty aldehydes and fatty alcohols derived from lipid catabolism (fatty aldehyde dehydrogenase, **ALDH3A2**) (*4-6*).

Gene expression markers of increased production of triacylglycerols (TAG) were also identified. One set of gene expression changes were associated with increased production of ceramide, which induces triglyceride production (*7-10*). These included (i) downregulation of the aldehyde hydrogenase **ALDH3A2**, which converts hexadecenal to hexadecenoic acid. The resulting hexadecenal accumulation may lead to ceramide production; (ii) downregulation of ceramidase ACER2, and (iii) upregulation of ceramide synthases (CERS3 and CERS5). An additional marker of triglyceride accumulation was the downregulation of the gene encoding palmitoyl-protein thioesterase 1 (**PPT1**), which expresses lipolytic activity (*11, 12*). TAG accumulation in *M. tuberculosis*-infected macrophages may also result from altered cellular redox. For example, we observed downregulation of genes encoding glutathione S-Transferase Mu (GSTM1, GSTM2, **GSTM4**), a scavenger of reactive oxygen species (ROS), which can induce lipid droplet formation by inducing the ASK1/p38/JNK pathway (*13-15*).

bioRxiv preprint doi: https://doi.org/10.1101/2023.06.08.542766; this version posted April 20, 2024. The copyright holder for this preprint (which was not certified by peer review) is the author/funder, who has granted bioRxiv a license to display the preprint in perpetuity. It is made available under aCC-BY 4.0 International license.

As mentioned in the main text, *M. tuberculosis*-infected macrophages exhibited downregulated TP53 gene and upregulated TP53-specific E3 ligases (FBXW7, MDM2) that target this factor for proteasomal degradation (*16-18*). Upregulation of XBP1 can also lead to decreased TP53 levels by inducing MDM2 (*19*). Reduced TP53 activity well correlates with downregulation of TP53-dependent Deptor, which negatively regulates mTOR (*20, 21*). mTORC1 signaling is lipogenic in multiple ways, including increased de novo lipid synthesis, reduced lipolysis, and reduced fatty acid oxidation (*22, 23*).

We also observed upregulation of metabolism-related ubiquitination processes, which was prominent both at the pathway- arufnd at the gene-level (**Fig. S4** and **Table S2a**). At the gene level, the top five upregulated genes featured genes encoding E3 ligases (**ZNRF1**) and associated proteins (**FZR1**, **FBXL20**, **UBE2H**) and the deubiquitinase **USP7,** which increases ZNRF1 activity as well as MDM2 activity (*24-29*). These changes presumably result in substantial macrophage proteome remodeling in response to *M. tuberculosis* infection. In particular, they may affect a variety of lipid metabolism regulators, including AKT1 and PLK1 (*30, 31*). **USP7** also stabilizes sirtuins (e.g., SIRT7, which is also upregulated at the gene level), resulting in increased expression of factors associated with TAG synthesis (*24, 32*).

2. Metabolism-related gene-level analysis of *C. neoformans*-infected monocyte-derived macrophages.

The top five up- and down-regulated genes (**Table S3a**) and functionally related, differentially expressed genes (**Table S3b**) were determined as described above for *M. tuberculosis* infection.

The top five upregulated genes encode glycolytic enzymes, strongly indicating a prominent role of a switch to glycolysis in *C. neoformans*-infected macrophages, which leads to increased triglyceride biosynthesis in multiple ways. Increased expression of hexokinase 2 (**HK2**) and other glycolytic enzymes (GPI, PFKP) results in increased production of fructose 1,6-bisphosphate, which can be converted to dihydroxyacetone phosphate (DHAP) and glyceraldehyde 3-phosphate (G3P), due to the increased production of aldolases (**ALDOC**, ALDOA). Also, the upregulation of the triosephosphate isomerase-encoding gene (TPI) might lead to increased production of DHAP, which is a precursor for triglyceride biosynthesis. The upregulation of additional glycolytic genes (**GAPDH**, **PGK1**, PGAM1, **ENO2**) might result in increased synthesis of phosphoenolpyruvate (PEP) (*33*). Increased PEP levels might inhibit

bioRxiv preprint doi: https://doi.org/10.1101/2023.06.08.542766; this version posted April 20, 2024. The copyright holder for this preprint (which was not certified by peer review) is the author/funder, who has granted bioRxiv a license to display the preprint in perpetuity. It is made available under aCC-BY 4.0 International license.

sarco/endoplasmic reticulum Ca2+-ATPase, resulting in calcium imbalance and endoplasmic reticulum stress, which ultimately lead to lipogenesis (*34-36*). Moreover, the products of glycolytic genes may favor lipid accumulation in ways other than their direct role in glycolysis. For example, the **HK2** product participates in the metabolic switch from oxidative phosphorylation to glycolysis (*37*) and inhibits lipases (*38*). In addition, phosphoglycerate kinase 1 (**PGK1**) can phosphorylate pyruvate dehydrogenase kinases, which are also upregulated (PDK1, PDK3), and inhibit the pyruvate dehydrogenase (PDH) complex (*39*). Inhibition of the PDH Complex, further compounded by the downregulation of one of its subunit-encoding genes (PDHA1), leads to impaired tricarboxylic acid cycle and enhanced glycolysis (*40*). As a result, pyruvate may accumulate and converted to lactate by lactate dehydrogenase (LDHA) (*41*), which we find upregulated. Lactate may inhibit lipolysis, thus resulting in lipid accumulation (*42*).

As mentioned in the main text, we also found indicators of reduced mitochondrial functions in *C. neoformans*-infected macrophages, including downregulation of polyribonucleotide nucleotidyl transferase 1 (**PNPT1**) and a glutaminyl-tRNA amidotransferase subunit 1 (**QRSL1**). The **PNPT1** product regulates the expression of the electron transport chain components at the mRNA and protein levels. Thus, downregulation of this gene might result in reduced function of the respiratory chain and decreased oxidative phosphorylation, leading to lipid accumulation (*43, 44*). The **QRSL1** product enables glutaminyl-tRNA synthase (glutamine-hydrolyzing) activity in the mitochondria. Missense mutations in the human QRSL1 locus have been associated with defects in mitochondrial respiratory chain complex and oxidative phosphorylation (*45, 46*). The resulting oxidative stress enhances lipogenesis (*47, 48*).

Lipid accumulation in *C. neoformans*-infected macrophages may also result from dysregulation of various other cellular activities. First, infected cells may exhibit increased fatty acid synthesis and decreased fatty acid oxidation due to the downregulation of (i) AMP-activated protein kinase (AMPK/PRKAA1), which inhibits de novo biosynthesis of fatty acids, increases fatty acid uptake, and stimulates fatty acid oxidation (*49-51*) and (ii) multiple genes encoding aldehyde dehydrogenases (ALDH3A2, ALDH16A1, ALDH1A3, ALDH4A1, ALDH1B1), which are involved in fatty acid oxidation (*6*). Second, perilipin genes (PLIN1, PLIN2), which are structural components of lipid droplets (*52*), are upregulated. PLIN gene upregulation may be associated, at least in part, with the downregulation of proteasome activator subunit 3 (**PSME3**) (*53*). The PSME3 product induces the degradation of Kruppel Like Factor 2 (KLF2) (*54*). Since KLF2 promotes the transcription of TAG synthesis and lipid storage genes, including PLIN1 and PLIN2 (*55*), increased KLF2 activity secondary to PSME3 downregulation is associated with lipid

bioRxiv preprint doi: https://doi.org/10.1101/2023.06.08.542766; this version posted April 20, 2024. The copyright holder for this preprint (which was not certified by peer review) is the author/funder, who has granted bioRxiv a license to display the preprint in perpetuity. It is made available under aCC-BY 4.0 International license.

accumulation (*56*). Third, one may hypothesize that another system involved in *C. neoformans*-infection-induced lipid accumulation is Hedgehog signaling. Inhibition of Hedgehog signaling results in fat accumulation (*57, 58*). Downregulation of ubiquitin C-terminal hydrolase L5 (**UCHL5**), which positively regulates Hedgehog signaling by deubiquitinating one of its components, the Smoothened (SMO) protein (*59, 60*), might result in reduced Hedgehog signaling and increased lipid accumulation. The observed upregulation of patched1 (PTCH1), which represses SMO (*61, 62*), is consistent with a role of Hedgehog in lipid accumulation in *C. neoformans*-infected macrophages. Fourth, the downregulation of anaphase promoting complex subunit 7 (**ANAPC7**) may also lead to triglyceride accumulation. ANAPC7, which is a component of the anaphase promoting complex/cyclosome (APC/C) (*63*), interacts with the CBP/p300 coactivator and stimulates its acetyl transferase activity (*64*). Among the CBP/p300 targets is farnesoid X receptor (FXR) (*65*). Acetylation of FXR leads to protein stabilization but reduces its activity as transcriptional regulator (*66*). Thus, reduced FXR acetylation resulting from ANAPC7 downregulation might activate FXR signaling. FXR signaling may induce lipid accumulation and reduced fatty acid oxidation (*67-69*). However, some reports show the opposite effect of FXR signaling on lipid accumulation (*70, 71*), suggesting perhaps context-specific effects.

3. Metabolism-related gene-level analysis of monocyte-derived macrophages treated with ACHN culture medium.

The top five up- and down-regulated genes (**Table S5a**) and functionally related, differentially expressed genes (**Table S5b**) were determined as described above for *M. tuberculosis* infection.

The top upregulated genes are enriched for glycolytic genes. Increased expression of hexokinase 3 (**HK3**) and other glycolytic genes (PFKFB4) might result in increased production of fructose 1,6-bisphosphate, which breaks down into dihydroxyacetone phosphate (DHAP) and glyceraldehyde 3-phosphate (G3P) (*37, 72*). Upregulation of triosephosphate isomerase (**TPI1**) also contributes to the conversion of DHAP to G3P, favoring triglyceride synthesis (*73*). G3P formation can also lead to synthesis of phosphoenolpyruvate (PEP), due to upregulation of other glycolytic genes (**PGK1**, **ENO2**) (*33*). Accumulation of PEP may result in enhanced production of pyruvate. Reduced utilization of pyruvate in the TCA cycle, which may be downregulated (see below), might lead to enhanced lipogenesis (*34*).

bioRxiv preprint doi: https://doi.org/10.1101/2023.06.08.542766; this version posted April 20, 2024. The copyright holder for this preprint (which was not certified by peer review) is the author/funder, who has granted bioRxiv a license to display the preprint in perpetuity. It is made available under aCC-BY 4.0 International license.

Macrophages treated with ACHN medium also exhibit transcriptional markers of reduced production/activity of the energy sensor AMP-activated protein kinase (AMPK). One is the upregulation of adenylate kinase 4 (**AK4**), which regulates cellular ATP levels and inhibits AMPK signaling (*74*). Another is the downregulation of phospholipase D family member 6 (**PLD6**), which activates AMPK by altering mitochondrial fusion and fission dynamics (*75*). The resulting decreased activity of AMPK, an energy sensor maintaining energy homeostasis (*76*), might result in increased YAP/TAZ signaling, which regulates metastasis and metabolic reprogramming in cancer cells (*77*), since AMPK inhibits YAP/TAZ via YAP phosphorylation (*78*). This scenario is suggested by the observed upregulation of genes associated with or regulated by YAP/TAZ, such as TEAD3, SLC2A5, and PLIN5, in ACHN-medium-treated macrophages. YAP/TAZ interacts with the TEAD transcription factors (*79*) to induce various target genes. One is the fructose transporter SLC2A5; its upregulation might result in increased fructose uptake, leading to lipogenesis via fructolysis (*80, 81*). YAP/TAZ also induces PLIN5, a member of a family of lipid-droplet-associated proteins (*82*), which protects triglycerides from enzymatic degradation by inhibiting adipose triglyceride lipase (*83, 84*). More in general, YAP/TAZ signaling and lipid metabolism are intricately intertwined in cancer cells, and YAP/TAZ is involved in the metabolism of fatty acids and sterols (*77*), suggesting a role of this signaling pathway also in the cholesterol accumulation detected in the ACHN-medium-treated cells.

We identified additional gene expression markers of lipid accumulation. Impaired fatty acid oxidation, which may result in lipid accumulation, is suggested by the downregulation of the acyl-CoA synthase **ACSM4** and functionally related genes, such as acyl-CoA conversion to acyl-carnitine (carnitine palmitoyl transferase I, CPT1) and oxidation of fatty aldehydes to fatty acids (aldehyde hydrogenases, ALDH genes) (*1-3, 6*). The downregulation of **N6AMT1**, which encodes a methyltransferase mostly implicated in DNA and protein modification, is also consistent with lipid accumulation, since this gene product has been associated with lipid catabolism and low triglyceride levels (*85, 86*) by yet unknown mechanisms.

One of the top five downregulated genes is peroxisome proliferator-activated receptor gamma coactivator 1-alpha (**PPARGC1a**), a transcriptional coactivator that regulates cellular metabolism (*87*). Downregulation of PPARGC1a is consistent with the observed downregulation of two of its target genes, NAD-dependent isocitrate dehydrogenase 3 catalytic subunit alpha (IDH3A) (*88*) and CD36. Isocitrate dehydrogenases catalyze the oxidative decarboxylation of isocitrate to 2-oxoglutarate, which is a rate-limiting step of the TCA cycle. Thus, IDH3A downregulation might result in TCA cycle disruption. An additional indicator of dampened TCA

bioRxiv preprint doi: https://doi.org/10.1101/2023.06.08.542766; this version posted April 20, 2024. The copyright holder for this preprint (which was not certified by peer review) is the author/funder, who has granted bioRxiv a license to display the preprint in perpetuity. It is made available under aCC-BY 4.0 International license.

cycle is the above-mentioned upregulation of **AK4**, since this adenylate kinase is associated with decreased expression of key TCA cycle genes (*89*). The scavenger receptor CD36 is a critical fatty acid sensor and regulator of lipid metabolism (*90, 91*). CD36 mediates fatty acid uptake, increases cholesterol efflux, and favors proteasomal degradation of HMG-CoA reductase, the rate-limiting enzyme in sterol synthesis (*92, 93*). CD36 downregulation might result in cholesterol accumulation, reduced fatty acid oxidation and mitochondrial function, and increased fatty acid storage (*92*). Thus, CD36 downregulation is consistent with increased accumulation of both cholesterol and triglycerides in ACHN-medium-treated cells. The decreased activity of AMPK proposed above would further decrease CD36 function, since AMPK promotes CD36 expression and translocation (*50*).

An additional marker of dysregulated cholesterol homeostasis is the downregulation of adenylate cyclase (**ADCY1**). In response to G-protein signaling, adenylate cyclases are activated and generate cyclic AMP (cAMP) (*94*). cAMP participates in various signal transduction mechanisms regulating adipogenesis, lipolysis, and cholesterol efflux (*94, 95*). Reduced cAMP production may be associated with decreased cholesterol efflux and decreased lipolysis (*94*-*97*).

bioRxiv preprint doi: https://doi.org/10.1101/2023.06.08.542766; this version posted April 20, 2024. The copyright holder for this preprint (which was not certified by peer review) is the author/funder, who has granted bioRxiv a license to display the preprint in perpetuity. It is made available under aCC-BY 4.0 International license.

Supplementary References

1. A. Rinaldi *et al.*, Impaired fatty acid metabolism perpetuates lipotoxicity along the transition to chronic kidney injury. *JCI Insight* **7**, (2022).
2. K. R. Dunning, M. R. Anastasi, V. J. Zhang, D. L. Russell, R. L. Robker, Regulation of fatty acid oxidation in mouse cumulus-oocyte complexes during maturation and modulation by PPAR agonists. *PLoS One* **9**, e87327 (2014).
3. F. Le Borgne, A. Ben Mohamed, M. Logerot, E. Garnier, J. Demarquoy, Changes in carnitine octanoyltransferase activity induce alteration in fatty acid metabolism. *Biochem Biophys Res Commun* **409**, 699-704 (2011).
4. W. B. Rizzo, Fatty aldehyde and fatty alcohol metabolism: review and importance for epidermal structure and function. *Biochim Biophys Acta* **1841**, 377-389 (2014).
5. W. B. Rizzo, Sjogren-Larsson syndrome: molecular genetics and biochemical pathogenesis of fatty aldehyde dehydrogenase deficiency. *Mol Genet Metab* **90**, 1-9 (2007).
6. S. Li, Z. Yang, H. Zhang, M. Peng, H. Ma, Potential role of ALDH3A2 on the lipid and glucose metabolism regulated by (-)-hydroxycitric acid in chicken embryos. *Anim Sci J* **90**, 961-976 (2019).
7. S. A. Summers, Ceramides: Nutrient Signals that Drive Hepatosteatosis. *J Lipid Atheroscler* **9**, 50-65 (2020).
8. K. Nakahara *et al.*, The Sjogren-Larsson syndrome gene encodes a hexadecenal dehydrogenase of the sphingosine 1-phosphate degradation pathway. *Mol Cell* **46**, 461-471 (2012).
9. M. Bektas *et al.*, Sphingosine 1-phosphate lyase deficiency disrupts lipid homeostasis in liver. *J Biol Chem* **285**, 10880-10889 (2010).
10. A. Maharaj *et al.*, Isolated glucocorticoid deficiency: Genetic causes and animal models. *J Steroid Biochem Mol Biol* **189**, 73-80 (2019).
11. R. Wang *et al.*, Identification of palmitoyl protein thioesterase 1 in human THP1 monocytes and macrophages and characterization of unique biochemical activities for this enzyme. *Biochemistry* **52**, 7559-7574 (2013).
12. A. Khaibullina *et al.*, In a model of Batten disease, palmitoyl protein thioesterase-1 deficiency is associated with brown adipose tissue and thermoregulation abnormalities. *PLoS One* **7**, e48733 (2012).
13. Y. Jin *et al.*, Glutathione S-transferase Mu 2 inhibits hepatic steatosis via ASK1 suppression. *Commun Biol* **5**, 326 (2022).

bioRxiv preprint doi: https://doi.org/10.1101/2023.06.08.542766; this version posted April 20, 2024. The copyright holder for this preprint (which was not certified by peer review) is the author/funder, who has granted bioRxiv a license to display the preprint in perpetuity. It is made available under aCC-BY 4.0 International license.

14. R. Zhuo *et al.*, Targeting Glutathione S-transferase M4 in Ewing sarcoma. *Front Pediatr* **2**, 83 (2014).
15. X. F. Leong, Lipid Oxidation Products on Inflammation-Mediated Hypertension and Atherosclerosis: A Mini Review. *Front Nutr* **8**, 717740 (2021).
16. D. Cui *et al.*, FBXW7 Confers Radiation Survival by Targeting p53 for Degradation. *Cell Rep* **30**, 497-509 e494 (2020).
17. J. Tang *et al.*, Critical role for Daxx in regulating Mdm2. *Nat Cell Biol* **8**, 855-862 (2006).
18. R. U. Janicke, D. Sohn, K. Schulze-Osthoff, The dark side of a tumor suppressor: anti-apoptotic p53. *Cell Death Differ* **15**, 959-976 (2008).
19. C. Huang *et al.*, Identification of XBP1-u as a novel regulator of the MDM2/p53 axis using an shRNA library. *Sci Adv* **3**, e1701383 (2017).
20. H. Chen *et al.*, DEP domain-containing mTOR-interacting protein suppresses lipogenesis and ameliorates hepatic steatosis and acute-on-chronic liver injury in alcoholic liver disease. *Hepatology* **68**, 496-514 (2018).
21. V. Catena, M. Fanciulli, Deptor: not only a mTOR inhibitor. *J Exp Clin Cancer Res* **36**, 12 (2017).
22. G. A. Soliman, The integral role of mTOR in lipid metabolism. *Cell Cycle* **10**, 861-862 (2011).
23. M. Laplante, D. M. Sabatini, An emerging role of mTOR in lipid biosynthesis. *Curr Biol* **19**, R1046-1052 (2009).
24. S. Ruf *et al.*, PLK1 (polo like kinase 1) inhibits MTOR complex 1 and promotes autophagy. *Autophagy* **13**, 486-505 (2017).
25. S. Zhu *et al.*, Regulation of Glucose, Fatty Acid and Amino Acid Metabolism by Ubiquitination and SUMOylation for Cancer Progression. *Front Cell Dev Biol* **10**, 849625 (2022).
26. S. Lambhate, D. Bhattacharjee, N. Jain, APC/C CDH1 ubiquitinates IDH2 contributing to ROS increase in mitosis. *Cell Signal* **86**, 110087 (2021).
27. X. Qiao, L. Zhang, A. M. Gamper, T. Fujita, Y. Wan, APC/C-Cdh1: from cell cycle to cellular differentiation and genomic integrity. *Cell Cycle* **9**, 3904-3912 (2010).
28. M. Li, C. L. Brooks, N. Kon, W. Gu, A dynamic role of HAUSP in the p53-Mdm2 pathway. *Mol Cell* **13**, 879-886 (2004).
29. W. Ni *et al.*, USP7 mediates pathological hepatic de novo lipogenesis through promoting stabilization and transcription of ZNF638. *Cell Death Dis* **11**, 843 (2020).

bioRxiv preprint doi: https://doi.org/10.1101/2023.06.08.542766; this version posted April 20, 2024. The copyright holder for this preprint (which was not certified by peer review) is the author/funder, who has granted bioRxiv a license to display the preprint in perpetuity. It is made available under aCC-BY 4.0 International license.

30. R. Miao *et al.*, Akt: A Potential Drug Target for Metabolic Syndrome. *Front Physiol* **13**, 822333 (2022).
31. Z. Zhang *et al.*, Plk1 inhibition enhances the efficacy of androgen signaling blockade in castration-resistant prostate cancer. *Cancer Res* **74**, 6635-6647 (2014).
32. L. Jiang *et al.*, Ubiquitin-specific peptidase 7 (USP7)-mediated deubiquitination of the histone deacetylase SIRT7 regulates gluconeogenesis. *J Biol Chem* **292**, 13296-13311 (2017).
33. M. Yang *et al.*, Hypoxia-induced MIF induces dysregulation of lipid metabolism in Hep2 laryngocarcinoma through the IL-6/JAK-STAT pathway. *Lipids Health Dis* **21**, 82 (2022).
34. P. C. Ho *et al.*, Phosphoenolpyruvate Is a Metabolic Checkpoint of Anti-tumor T Cell Responses. *Cell* **162**, 1217-1228 (2015).
35. J. Bi *et al.*, Seipin promotes adipose tissue fat storage through the ER Ca(2)(+)-ATPase SERCA. *Cell Metab* **19**, 861-871 (2014).
36. S. Fu *et al.*, Aberrant lipid metabolism disrupts calcium homeostasis causing liver endoplasmic reticulum stress in obesity. *Nature* **473**, 528-531 (2011).
37. L. H. Fairley *et al.*, Mitochondrial control of microglial phagocytosis by the translocator protein and hexokinase 2 in Alzheimer's disease. *Proc Natl Acad Sci U S A* **120**, e2209177120 (2023).
38. L. Leng *et al.*, Microglial hexokinase 2 deficiency increases ATP generation through lipid metabolism leading to β-amyloid clearance. *Nat Metab* **4**, 1287-1305 (2022).
39. X. Li *et al.*, Mitochondria-Translocated PGK1 Functions as a Protein Kinase to Coordinate Glycolysis and the TCA Cycle in Tumorigenesis. *Mol Cell* **61**, 705-719 (2016).
40. L. Xu *et al.*, SIRT3 elicited an anti-Warburg effect through HIF1alpha/PDK1/PDHA1 to inhibit cholangiocarcinoma tumorigenesis. *Cancer Med* **8**, 2380-2391 (2019).
41. S. Chen, L. Zhou, J. Sun, Y. Qu, M. Chen, The Role of cAMP-PKA Pathway in Lactate-Induced Intramuscular Triglyceride Accumulation and Mitochondria Content Increase in Mice. *Front Physiol* **12**, 709135 (2021).
42. C. Liu *et al.*, Lactate inhibits lipolysis in fat cells through activation of an orphan G-protein-coupled receptor, GPR81. *J Biol Chem* **284**, 2811-2822 (2009).
43. V. Vedrenne *et al.*, Mutation in PNPT1, which encodes a polyribonucleotide nucleotidyltransferase, impairs RNA import into mitochondria and causes respiratory-chain deficiency. *Am J Hum Genet* **91**, 912-918 (2012).

bioRxiv preprint doi: https://doi.org/10.1101/2023.06.08.542766; this version posted April 20, 2024. The copyright holder for this preprint (which was not certified by peer review) is the author/funder, who has granted bioRxiv a license to display the preprint in perpetuity. It is made available under aCC-BY 4.0 International license.

44. G. Wang *et al.*, PNPASE regulates RNA import into mitochondria. *Cell* **142**, 456-467 (2010).

45. M. Kohda *et al.*, A Comprehensive Genomic Analysis Reveals the Genetic Landscape of Mitochondrial Respiratory Chain Complex Deficiencies. *PLoS Genet* **12**, e1005679 (2016).

46. F. Dursun *et al.*, Primary adrenal insufficiency in a patient with biallelic QRSL1 mutations. *Eur J Endocrinol* **187**, K27-K32 (2022).

47. K. Puri *et al.*, Diabetes Mellitus Severity and a Switch From Using Lipoprotein Lipase to Adipose-Derived Fatty Acid Results in a Cardiac Metabolic Signature That Embraces Cell Death. *J Am Heart Assoc* **8**, e014022 (2019).

48. D. Hathazi *et al.*, Metabolic shift underlies recovery in reversible infantile respiratory chain deficiency. *EMBO J* **39**, e105364 (2020).

49. M. Foretz, P. C. Even, B. Viollet, AMPK Activation Reduces Hepatic Lipid Content by Increasing Fat Oxidation In Vivo. *Int J Mol Sci* **19**, (2018).

50. W. Wu *et al.*, AMPK facilitates intestinal long-chain fatty acid uptake by manipulating CD36 expression and translocation. *FASEB J* **34**, 4852-4869 (2020).

51. H. Wu *et al.*, Lipid droplet-dependent fatty acid metabolism controls the immune suppressive phenotype of tumor-associated macrophages. *EMBO Mol Med* **11**, e10698 (2019).

52. D. L. Brasaemle, Thematic review series: adipocyte biology. The perilipin family of structural lipid droplet proteins: stabilization of lipid droplets and control of lipolysis. *J Lipid Res* **48**, 2547-2559 (2007).

53. L. C. Lin, A. C. Gao, C. H. Lai, J. T. Hsieh, H. Lin, Induction of neuroendocrine differentiation in castration resistant prostate cancer cells by adipocyte differentiation-related protein (ADRP) delivered by exosomes. *Cancer Lett* **391**, 74-82 (2017).

54. J. Sun *et al.*, The 11S Proteasome Subunit PSME3 Is a Positive Feedforward Regulator of NF-kappaB and Important for Host Defense against Bacterial Pathogens. *Cell Rep* **14**, 737-749 (2016).

55. V. G. Yerra, K. Drosatos, Specificity Proteins (SP) and Krüppel-like Factors (KLF) in Liver Physiology and Pathology. *Int J Mol Sci* **24**, (2023).

56. J. Ling *et al.*, Defective lipid metabolism associated with mutation in klf-2 and klf-3: important roles of essential dietary salts in fat storage. *Nutr Metab (Lond)* **14**, 22 (2017).

57. M. Matz-Soja *et al.*, Hedgehog signaling is a potent regulator of liver lipid metabolism and reveals a GLI-code associated with steatosis. *Elife* **5**, (2016).

bioRxiv preprint doi: https://doi.org/10.1101/2023.06.08.542766; this version posted April 20, 2024. The copyright holder for this preprint (which was not certified by peer review) is the author/funder, who has granted bioRxiv a license to display the preprint in perpetuity. It is made available under aCC-BY 4.0 International license.

58. G. D. Dalton *et al.*, Hepatocyte activity of the cholesterol sensor smoothened regulates cholesterol and bile acid homeostasis in mice. *iScience* **24**, 103089 (2021).
59. Z. Zhou *et al.*, The deubiquitinase UCHL5/UCH37 positively regulates Hedgehog signaling by deubiquitinating Smoothened. *J Mol Cell Biol* **10**, 243-257 (2018).
60. R. Tu *et al.*, The emerging role of deubiquitylating enzymes as therapeutic targets in cancer metabolism. *Cancer Cell Int* **22**, 130 (2022).
61. K. Petrov, T. de Almeida Magalhaes, A. Salic, Mechanism and ultrasensitivity in Hedgehog signaling revealed by Patched1 disease mutations. *Proc Natl Acad Sci U S A* **118**, (2021).
62. U. Styrkarsdottir *et al.*, Sequence variants in the PTCH1 gene associate with spine bone mineral density and osteoporotic fractures. *Nat Commun* **7**, 10129 (2016).
63. J. M. Peters, The anaphase-promoting complex: proteolysis in mitosis and beyond. *Mol Cell* **9**, 931-943 (2002).
64. J. Liu, S. Y. Fuchs, Cross-talk between APC/C and CBP/p300. *Cancer Biol Ther* **5**, 760-762 (2006).
65. S. Fang *et al.*, The p300 acetylase is critical for ligand-activated farnesoid X receptor (FXR) induction of SHP. *J Biol Chem* **283**, 35086-35095 (2008).
66. J. K. Kemper *et al.*, FXR acetylation is normally dynamically regulated by p300 and SIRT1 but constitutively elevated in metabolic disease states. *Cell Metab* **10**, 392-404 (2009).
67. K. H. Kim *et al.*, Hepatic FXR/SHP axis modulates systemic glucose and fatty acid homeostasis in aged mice. *Hepatology* **66**, 498-509 (2017).
68. K. Boulias *et al.*, Regulation of hepatic metabolic pathways by the orphan nuclear receptor SHP. *EMBO J* **24**, 2624-2633 (2005).
69. M. Abdelkarim *et al.*, The farnesoid X receptor regulates adipocyte differentiation and function by promoting peroxisome proliferator-activated receptor-gamma and interfering with the Wnt/beta-catenin pathways. *J Biol Chem* **285**, 36759-36767 (2010).
70. Y. Jiao, Y. Lu, X. Y. Li, Farnesoid X receptor: a master regulator of hepatic triglyceride and glucose homeostasis. *Acta Pharmacol Sin* **36**, 44-50 (2015).
71. G. Y. Wu *et al.*, MicroRNA-122 Inhibits Lipid Droplet Formation and Hepatic Triglyceride Accumulation via Yin Yang 1. *Cell Physiol Biochem* **44**, 1651-1664 (2017).
72. W. Xu *et al.*, Hexokinase 3 dysfunction promotes tumorigenesis and immune escape by upregulating monocyte/macrophage infiltration into the clear cell renal cell carcinoma microenvironment. *Int J Biol Sci* **17**, 2205-2222 (2021).

bioRxiv preprint doi: https://doi.org/10.1101/2023.06.08.542766; this version posted April 20, 2024. The copyright holder for this preprint (which was not certified by peer review) is the author/funder, who has granted bioRxiv a license to display the preprint in perpetuity. It is made available under aCC-BY 4.0 International license.

73. G. A. Rao, M. F. Sorrels, R. Reiser, Biosynthesis of triglycerides from triose phosphates by microsomes of intestinal mucosa. *Lipids* **5**, 762-764 (1970).
74. N. J. Lanning *et al.*, A mitochondrial RNAi screen defines cellular bioenergetic determinants and identifies an adenylate kinase as a key regulator of ATP levels. *Cell Rep* **7**, 907-917 (2014).
75. B. von Eyss *et al.*, A MYC-Driven Change in Mitochondrial Dynamics Limits YAP/TAZ Function in Mammary Epithelial Cells and Breast Cancer. *Cancer Cell* **28**, 743-757 (2015).
76. G. R. Steinberg, D. G. Hardie, New insights into activation and function of the AMPK. *Nat Rev Mol Cell Biol* **24**, 255-272 (2023).
77. H. Yamaguchi, G. M. Taouk, A Potential Role of YAP/TAZ in the Interplay Between Metastasis and Metabolic Alterations. *Front Oncol* **10**, 928 (2020).
78. J. S. Mo *et al.*, Cellular energy stress induces AMPK-mediated regulation of YAP and the Hippo pathway. *Nat Cell Biol* **17**, 500-510 (2015).
79. I. M. Moya, G. Halder, Hippo-YAP/TAZ signalling in organ regeneration and regenerative medicine. *Nat Rev Mol Cell Biol* **20**, 211-226 (2019).
80. H. D. Huh, D. H. Kim, H. S. Jeong, H. W. Park, Regulation of TEAD Transcription Factors in Cancer Biology. *Cells* **8**, (2019).
81. K. W. Ter Horst, M. J. Serlie, Fructose Consumption, Lipogenesis, and Non-Alcoholic Fatty Liver Disease. *Nutrients* **9**, (2017).
82. H. Itabe, T. Yamaguchi, S. Nimura, N. Sasabe, Perilipins: a diversity of intracellular lipid droplet proteins. *Lipids Health Dis* **16**, 83 (2017).
83. H. Wang *et al.*, Unique regulation of adipose triglyceride lipase (ATGL) by perilipin 5, a lipid droplet-associated protein. *J Biol Chem* **286**, 15707-15715 (2011).
84. L. L. Listenberger, A. G. Ostermeyer-Fay, E. B. Goldberg, W. J. Brown, D. A. Brown, Adipocyte differentiation-related protein reduces the lipid droplet association of adipose triglyceride lipase and slows triacylglycerol turnover. *J Lipid Res* **48**, 2751-2761 (2007).
85. R. C. Slieker *et al.*, Identification of biomarkers for glycaemic deterioration in type 2 diabetes. *Nat Commun* **14**, 2533 (2023).
86. Z. Si *et al.*, Identification of CYP46A1 as a new regulator of lipid metabolism through CRISPR-based whole-genome screening. *Faseb j* **34**, 13776-13791 (2020).
87. C. F. Cheng, H. C. Ku, H. Lin, PGC-1α as a Pivotal Factor in Lipid and Metabolic Regulation. *Int J Mol Sci* **19**, (2018).

bioRxiv preprint doi: https://doi.org/10.1101/2023.06.08.542766; this version posted April 20, 2024. The copyright holder for this preprint (which was not certified by peer review) is the author/funder, who has granted bioRxiv a license to display the preprint in perpetuity. It is made available under aCC-BY 4.0 International license.

88. Q. Zhang *et al.*, MiR-30b-5p regulates the lipid metabolism by targeting PPARGC1A in Huh-7 cell line. *Lipids Health Dis* **19**, 76 (2020).
89. K. Fujisawa *et al.*, Modulation of anti-cancer drug sensitivity through the regulation of mitochondrial activity by adenylate kinase 4. *Journal of Experimental & Clinical Cancer Research* **35**, 48 (2016).
90. Y. Li *et al.*, CD36 favours fat sensing and transport to govern lipid metabolism. *Prog Lipid Res* **88**, 101193 (2022).
91. R. L. Silverstein, M. Febbraio, CD36, a scavenger receptor involved in immunity, metabolism, angiogenesis, and behavior. *Sci Signal* **2**, re3 (2009).
92. L. Marechal *et al.*, The CD36-PPARgamma Pathway in Metabolic Disorders. *Int J Mol Sci* **19**, (2018).
93. A. Rodrigue-Way *et al.*, Scavenger receptor CD36 mediates inhibition of cholesterol synthesis via activation of the PPARγ/PGC-1α pathway and Insig1/2 expression in hepatocytes. *Faseb j* **28**, 1910-1923 (2014).
94. M. Rogne, K. Tasken, Compartmentalization of cAMP signaling in adipogenesis, lipogenesis, and lipolysis. *Horm Metab Res* **46**, 833-840 (2014).
95. W. Tang *et al.*, Adenylyl cyclase 1 as a major isoform to generate cAMP signaling for apoA-1-mediated cholesterol efflux pathway. *J Lipid Res* **59**, 635-645 (2018).
96. B. M. Stenson *et al.*, Liver X receptor (LXR) regulates human adipocyte lipolysis. *J Biol Chem* **286**, 370-379 (2011).
97. P. Arner, D. P. Andersson, J. Backdahl, I. Dahlman, M. Ryden, Weight Gain and Impaired Glucose Metabolism in Women Are Predicted by Inefficient Subcutaneous Fat Cell Lipolysis. *Cell Metab* **28**, 45-54 e43 (2018).
