# Supplementary material for: Heterogeneity of foam cell biogenesis across diseases
Source: bioRxiv. 2023 Jul 19:2023.06.08.542766. Originally published 2023 Jun 8. Preprint. [Version 2] doi: 10.1101/2023.06.08.542766 (PMC10274805; doi:10.1101/2023.06.08.542766)
Supplement: Supplementary file 1 [file media-1.pdf]

Supplementary Information

Heterogeneity of foam cell biogenesis across diseases

Valentina Guerrini[1*], Brendan Prideaux[2], Rehan Khan[1], Selvakumar Subbian[1], Yina Wang[3], Evita Sadimin[4*], Siddhi Pawar[3], Rahul Ukey[1], Eric A. Singer[4*], Chaoyang Xue[3], Maria Laura Gennaro[1**]

Supplementary Figures

Fig. S1. Low-magnification images of murine lungs uninfected and infected with *C. neoformans* H99.

Fig. S2. Hematoxylin-eosin staining and MALDI imaging of uninfected murine lung sections.

Fig. S3. Macrophage lipid droplet accumulation in response to infection with live *C. neoformans*, or treatment with cell-free fungal culture medium, or with heat-killed fungal cells.

Fig. S4. Metabolism-associated pathways (A) upregulated in *M. tuberculosis*-infected human monocyte-derived macrophages and (B) downregulated in *C. neoformans*-infected human monocyte-derived macrophages.

Fig. S5. Effect of TNFα on lipid droplet accumulation in human monocyte-derived macrophages.

Fig. S6. Spatial distributions of cholesterol ester (CE) species in papillary renal cell carcinoma (pRCC).

Fig.S7. Spatial distribution of triglyceride (TAG) species in papillary renal cell carcinoma (pRCC).

Fig. S8. Production of cytokines by human monocyte-derived macrophages treated with ACHN-conditioned culture medium and role of IL-8 in lipid droplet accumulation.

Fig. S9. Metabolism-associated pathways downregulated in ACHN-medium-treated human monocyte-derived macrophages.

Supplementary Tables

Table S1. List of triglyceride and cholesteryl ester species detected in tissues by MALDI-MSI.

Table S2a. Top five down- and up-regulated genes in macrophages infected with *M. tuberculosis.*

Table S2b. Genes functionally related to top five down- and up-regulated genes in macrophages infected with *M. tuberculosis.*

Table S3a. Top five down- and up-regulated genes in macrophages infected with *C. neoformans.*

Table S3b. Genes functionally related to top five down- and up-regulated genes in macrophages infected with *C. neoformans.*

Table S4. Levels of cytokines in supernatants of ACHN cell cultures and MDM treated with ACHN-conditioned culture medium.

Table S5a. Top five down- and up-regulated genes in macrophages treated with ACHN-conditioned medium.

Table S5b. Genes functionally related to top five down- and up-regulated genes in macrophages treated with ACHN-conditioned culture medium.

Supplementary Materials and Methods

Supplementary Text on transcriptomics analyses

Supplementary References

Supplementary Figures

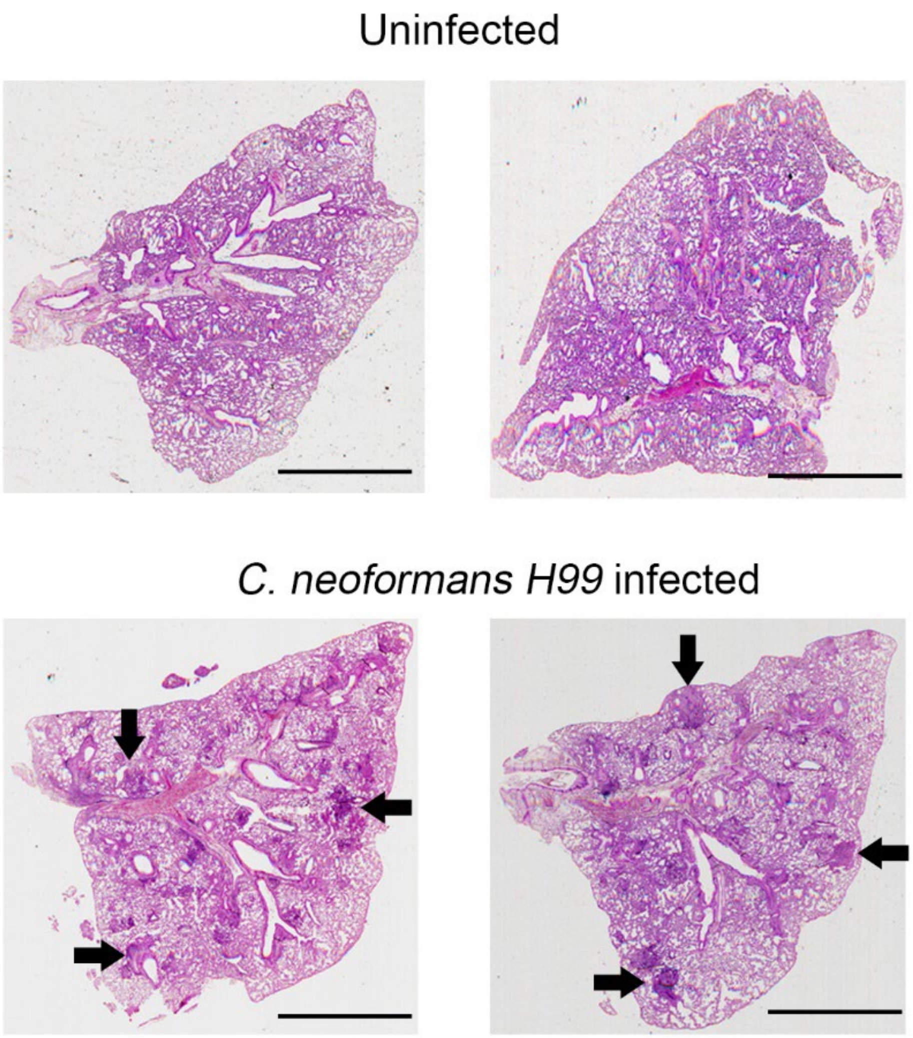

Supplementary Fig. S1. Low-magnification images of murine lungs uninfected and infected with *C. neoformans* H99. H&E staining of uninfected (top panels) and infected (bottom panels) lung sections. Black arrows show cellular aggregates in the infected tissue. Images were photographed at 20x magnification; scale bar is 2 mm.

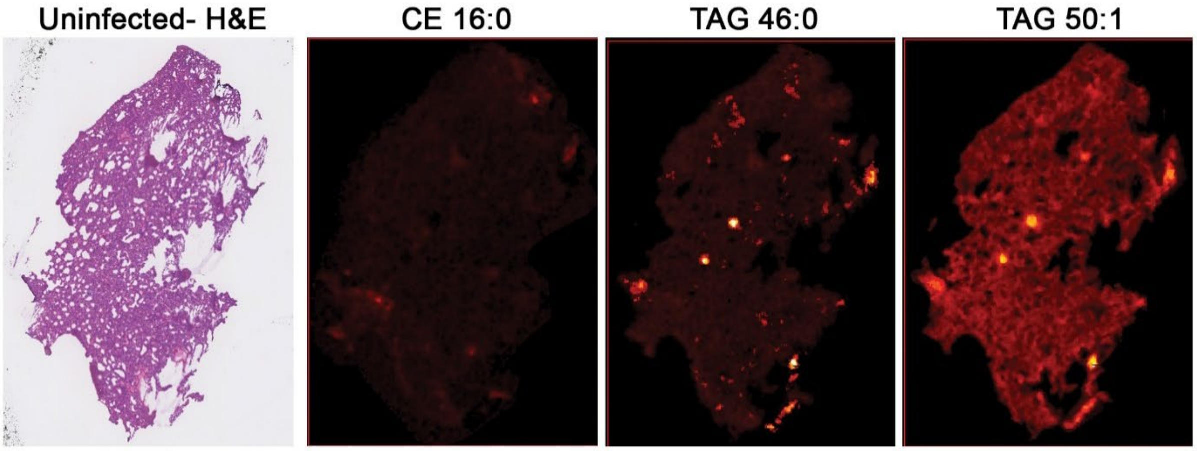

Supplementary Fig. S2. Hematoxylin-eosin staining and MALDI imaging of uninfected murine lung sections. The left panel shows H&E staining of uninfected lung sections. The three additional panels show MALDI imaging of storage lipids in lung sections contiguous to those used for H&E staining. Representative species are shown, CE (16:0) and TAG (46:0 and 50:1), corresponding to those shown in Fig. 1C (TAG and CE species detected are listed in **Table S1**).

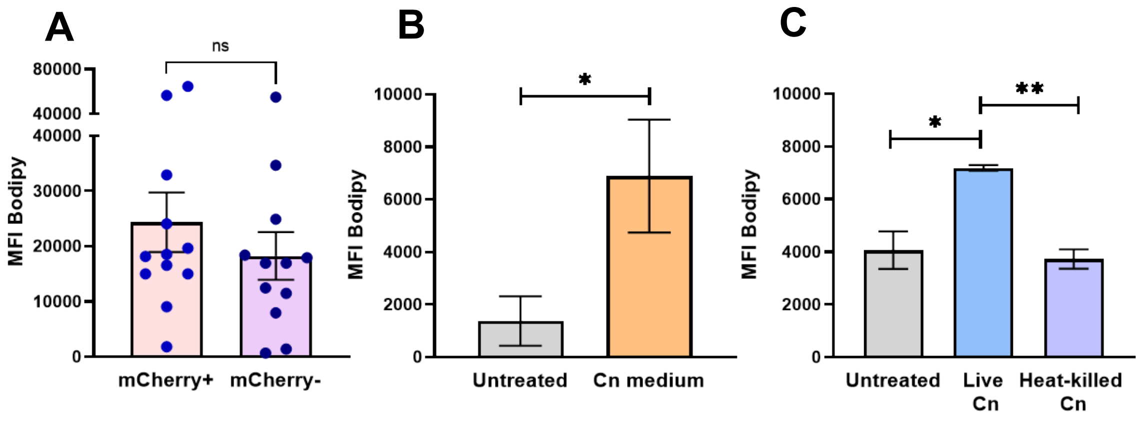

Supplementary Fig. S3. Macrophage lipid droplet accumulation in response to infection with live *C. neoformans*, or treatment with cell-free fungal culture medium, or with heat-killed fungal cells. In all panels, lipid droplet content of monocyte-derived macrophages (MDM) was measured by imaging flow cytometry and expressed as median fluorescence intensity (MFI) of Bodipy 493/503. **A.** MDM were infected with *C. neoformans* expressing mCherry and lipid droplet content was measured at 24 h post-infection in macrophages in the infected culture wells carrying or not carrying intracellular fungi (mCherry+ and mCherry-). Each dot represents one human donor. **B.** MDM were treated for 7 days with cell-free culture filtrate of *C. neoformans* grown in RPMI medium (Cn medium) or sterile RPMI medium (untreated). **C.** THP1 cells were treated with live or heat-killed *C. neoformans* (Cn) for 24 h. Statistics: mean and SEM (A-B) and mean and SD of triplicates (C-D) are shown; ns, non-significant; *, p <0.05; **, p <0.01 (paired t-test).

A. ***M. tuberculosis***

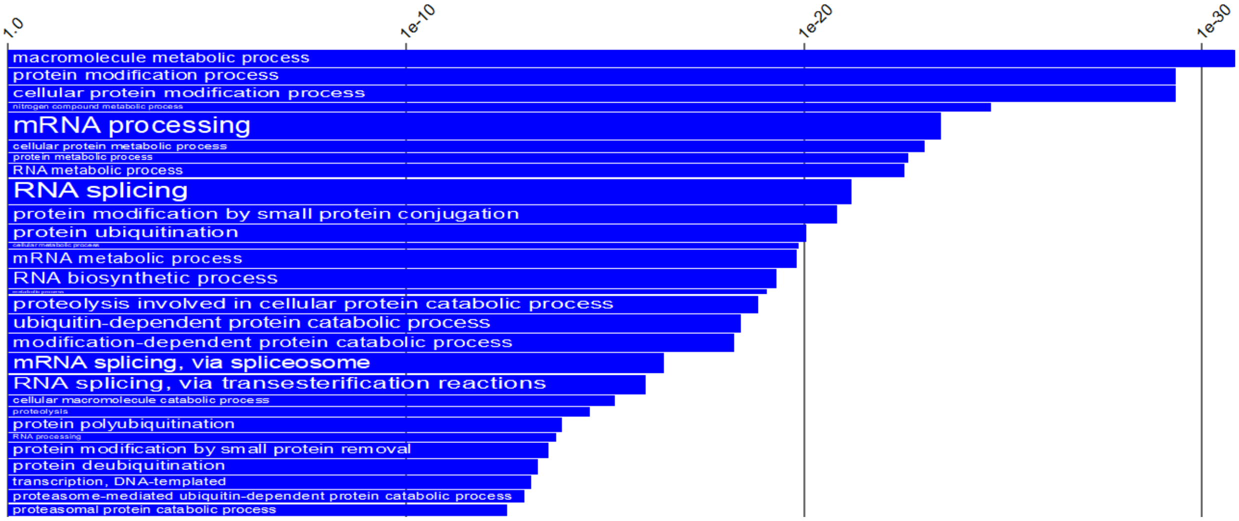

B. ***C. neoformans***

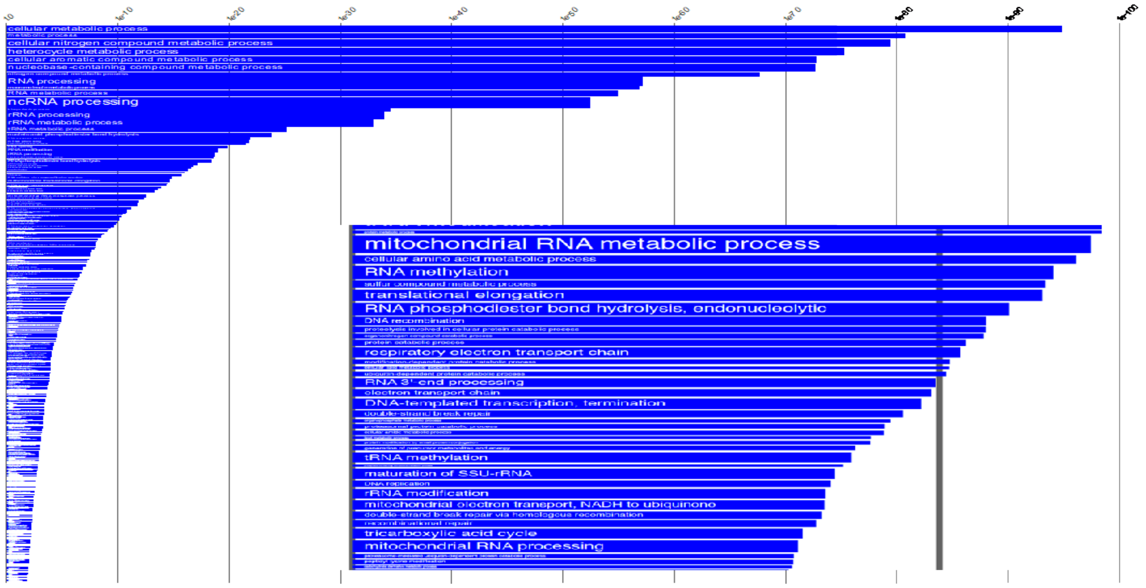

Supplementary Fig. S4. A. Upregulated metabolism-associated pathways in *M. tuberculosis*-infected human monocyte-derived macrophages. B. Downregulated metabolism-associated pathways in *C. neoformans*-infected human monocyte-derived macrophages. The panels show Gene Ontology (GO) annotations related to metabolic processes that were upregulated in *M. tuberculosis*-infected MDM and downregulated in *C. neoformans*-infected MDM, relative to uninfected control cells. The differential expression between sample classes (infected vs uninfected) was tested with coincident extreme ranks in numerical observations (CERNO). Pathways were selected using a cutoff false discovery rate of

0.05; the *p*-values for these pathways were plotted on the *x*-axis. To represent effect size, pathway gene sets containing fewer genes were given greater bar height/font size than were larger sets that yielded similar *p* values. For visualization purposes, panel A shows top-ranking annotations, and panel B. contains an inset highlighting mitochondria-related annotations.

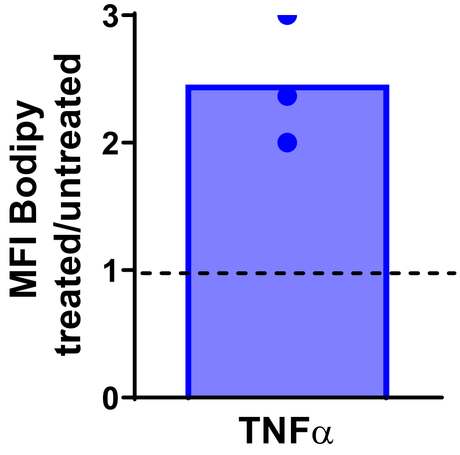

Supplementary Fig. S5. Effect of TNFα on lipid droplet accumulation in human monocyte-derived macrophages. MDM were stimulated with recombinant TNFα (2.5-40 ng/ml – different donors showed maximal effect at different doses within this range) and lipid droplet content was measured by imaging flow cytometry, as described in Fig. 2. Each dot represents a donor. *, p <0.05 (one sample t-test).

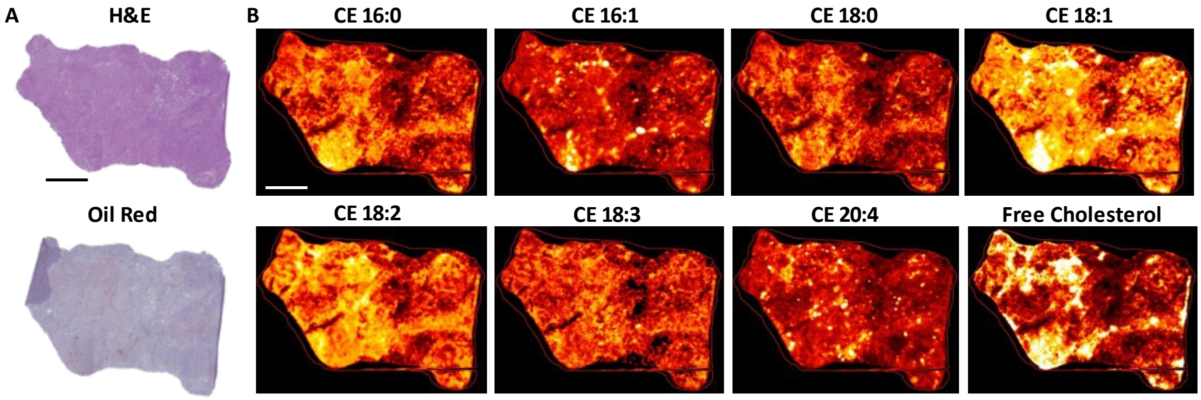

Supplementary Fig. S6. Spatial distribution of cholesterol ester (CE) species in papillary renal cell carcinoma (pRCC). A. Hematoxylin-eosin- and oil-red-stained kidney sections contiguous to those used for MALDI imaging. **B.** MALDI-2-MSI signal distribution for the major detected cholesterol ester species. Scale bar = 5 mm

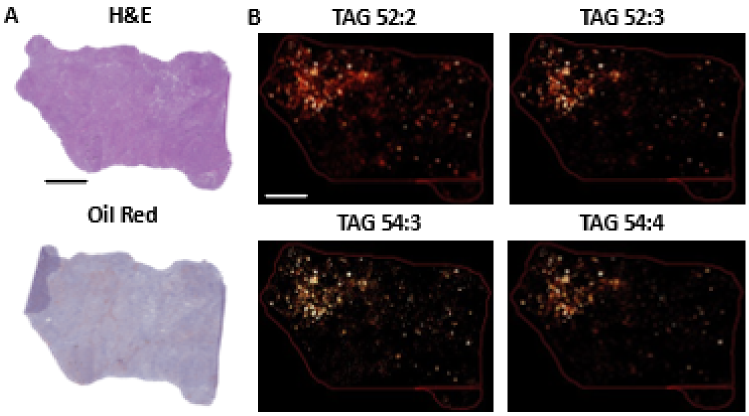

Supplementary Fig. S7. Spatial distribution of triglyceride (TAG) species in papillary renal cell carcinoma (pRCC). A. Hematoxylin-eosin- and oil-red-stained kidney sections contiguous to those used for MALDI imaging. **B.** MALDI-2-MSI signal distribution for four of the major detected TAG species. Scale bar = 5 mm

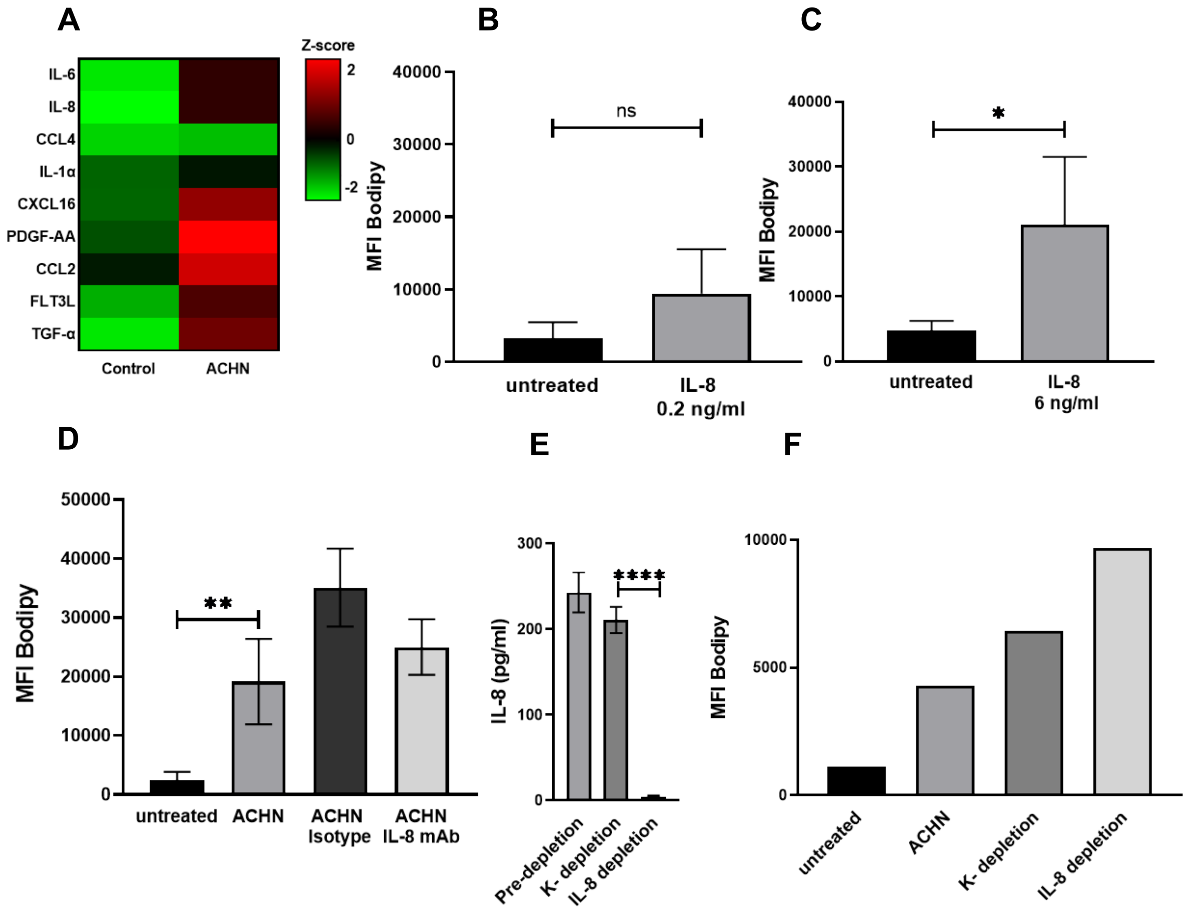

Supplementary Fig. S8. Production of cytokines by human monocyte-derived macrophages treated with ACHN-conditioned culture medium and role of IL-8 in lipid droplet accumulation. A. The heat map shows cytokines and chemokines differentially expressed by MDM exposed to conditioned medium from ACHN cell cultures relative to control, untreated MDM, as described in Fig. 4. Cytokines/chemokines were measured as technical duplicates using a 43-plex array and Luminex technology. The average *Z*-score for five donors was calculated for each analyte. Color gradient from green to red represents increasing concentrations. Data shown had $p < 0.05$ (paired *t*-test). **B-C.** Lipid droplet content of MDM was measured as median fluorescence intensity (MFI) of Bodipy 493/503 by imaging flow cytometry, following 7-day treatment with recombinant IL-8, at two different concentrations, as indicated. **D.** MDM were left untreated or treated for 7 days with ACHN-conditioned medium +/- anti-IL-8 neutralizing and isotype control antibodies (both at 1 µg/ml final concentration). Bodipy MFI was determined as in panels B-C. **E.** ACHN medium was depleted of IL-8 by incubation with

magnetic beads conjugated with anti-IL-8 antibodies, and with negative control, unconjugated beads (K- depletion). IL-8 levels in the medium, pre-and post-depletion, were measured by ELISA. **F.** Lipid droplet content of MDM was measured (as in panels B-C) in MDM left untreated, treated for 7 days with conditioned ACHN medium left untreated (ACHN), mock depleted (K- depletion), or IL-8 depleted, as indicated. Data are shown as means of duplicate measurements. Statistics: mean and SD of triplicate measurements (B-D-E) and mean and SEM of multiple donors (C) are shown; ns, non-significant; *, p <0.05; **, p <0.01, ****, p <0.001 (B-D-E, unpaired t-test; C, Mann-Whitney test).

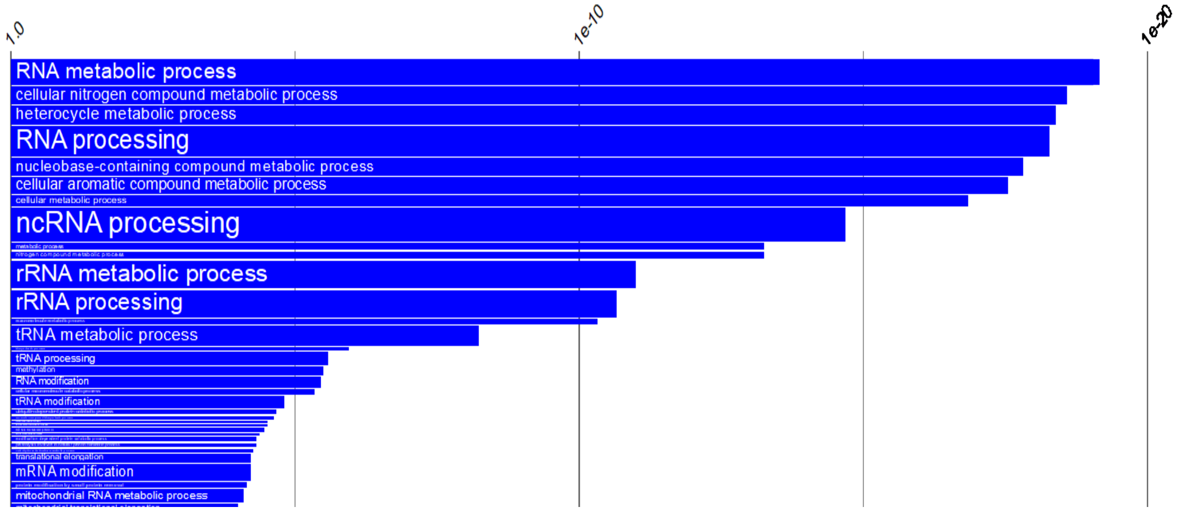

Supplementary Fig. S9. Downregulated metabolism-associated pathways in ACHN-medium-treated human monocyte-derived macrophages. The figure shows Gene Ontology (GO) annotations related to metabolic processes that were downregulated in MDM treated with ACHN conditioned medium, relative to untreated control cells. The differential expression between sample classes (treated vs untreated) was tested and selected pathways were represented as described in Figure S4. For visualization purposes, top-ranking annotations are shown.

Supplementary Tables

Supplementary Table S1. List of triglyceride and cholesteryl ester species detected in tissues by MALDI-MSI.

TAG	Detected in *Cryptococcus*	Detected in pRCC	CE	Detected in *Cryptococcus*	Detected in pRCC
**Species**	**Tissue**	**Tissue**	**Species**	**Tissue**	**Tissue**
**48:0**	✓	✗	**16:0**	✓	✓
**48:1**	✓	✗	**16:1**	✓	✓
**48:2**	✓	✗	**18:0**	✓	✓
**48:3**	✓	✗	**18:1**	✓	✓
**50:0**	✓	✗	**18:2**	✓	✓
**50:1**	✓	✓	**18:3**	✓	✓
**50:2**	✓	✓	**20:1**	✓	✓
**50:3**	✓	✓	**20:2**	✗	✓
**50:4**	✓	✗	**20:4**	✓	✓
**52:1**	✓	✗			
**52:2**	✓	✓			
**52:3**	✓	✓			
**52:4**	✓	✓			
**52:5**	✓	✓			
**52:6**	✓	✗			
**54:2**	✓	✓			
**54:3**	✓	✓			
**54:4**	✓	✓			
**54:5**	✓	✓			
**54:6**	✓	✗			
**54:7**	✓	✗			
**56:3**	✓	✗			
**56:4**	✓	✗			

Triglyceride (TAG) species (identified by the total number of carbons and double bonds in the esterified fatty acids) and cholesteryl ester (CE) species detected by MALDI-MSI in the cryptococcus-infected lung tissues and papillary renal cell carcinoma (pRCC) tissue. ✓, detected; x, not detected.

Supplementary Table S2a. Top five down- and up-regulated genes in macrophages infected with *M. tuberculosis.*

Gene ID	Gene	log2FoldChange	*p* value
ENSG00000005469	CROT	-0.694245982	6.81E-10
ENSG00000183549	ACSM5	-1.875070163	2.15E-12
ENSG00000072210	ALDH3A2	-0.675169083	1.18E-15
ENSG00000131238	PPT1	-0.769679647	6.47E-16
ENSG00000168765	GSTM4	-0.846851963	4.64E-14
ENSG00000105325	FZR1	0.451795	1.01E-10
ENSG00000186187	ZNRF1	0.884946	4.79E-17
ENSG00000108306	FBXL20	0.849817	9.11E-17
ENSG00000186591	UBE2H	0.795385	3.10E-25
ENSG00000187555	USP7	0.206178	4.81E-06

Genes in differentially expressed metabolism-related Gene Ontology gene sets having FDR < 0.05 were selected based on individual *p* value ($p << 0.05$) and ranked by log2 fold change (infected vs non-infected) and number of occurrences in the differentially expressed gene sets. The top five down- and up-regulated genes were selected for further analysis.

Supplementary Table S2b. Genes functionally related with the top five down- and up-regulated genes in macrophages infected with *M. tuberculosis*.

Gene ID	Gene	log2FoldChange	*p* value
ENSG00000215009	ACSM4	-1.65627	2.66E-05
ENSG00000130377	ACSBG2	-1.09841	3.99E-03
ENSG00000140284	SLC27A2	0.929524	2.40E-03
ENSG00000143554	SLC27A3	0.440446	2.69E-02
ENSG00000110090	CPT1A	-0.69747	6.60E-05
ENSG00000157184	CPT2	-0.32849	3.84E-05
ENSG00000154227	CERS3	1.207873	6.71E-03
ENSG00000139624	CERS5	0.437953	5.94E-06
ENSG00000177076	ACER2	-0.46598	2.77E-02
ENSG00000141510	TP53	-0.34037	1.19E-04
ENSG00000109670	FBXW7	0.514687	0.00000987
ENSG00000135679	MDM2	0.745793	1.91E-10
ENSG00000100219	XBP1	1.329735	1.36E-11
ENSG00000155792	DEPTOR	-2.04205	0.0000007
ENSG00000164181	ELOVL7	4.018986	1.04E-33
ENSG00000175445	LPL	-1.17544	6.57E-08
ENSG00000134184	GSTM1	-0.95459	0.000000069
ENSG00000213366	GSTM2	-1.23318	0.000000626
ENSG00000166851	PLK1	-1.07326	0.0025
ENSG00000092964	DPYSL2	-0.9294	1.38E-06
ENSG00000013588	GPRC5A	6.429513	2.92E-81
ENSG00000181773	GPR3	3.096538	1.07E-34
ENSG00000183484	GPR132	3.238435	2.91E-26
ENSG00000180758	GPR157	2.357273	5.3E-21
ENSG00000173698	GPR64	2.313555	8.38E-17
ENSG00000139572	GPR84	2.131323	4.78E-10
ENSG00000182885	GPR97	2.623284	1.95E-09
ENSG00000188316	ENO4	-1.32246	7.67E-07
ENSG00000077721	UBE2A	0.552901	1.40E-08
ENSG00000160087	UBE2J2	0.405355	2.54E-08
ENSG00000177889	UBE2N	0.348958	3.68E-08
ENSG00000131508	UBE2D2	0.331604	7.16E-07
ENSG00000185651	UBE2L3	0.381012	3.78E-06
ENSG00000160714	UBE2Q1	0.35768	2.01E-05
ENSG00000072401	UBE2D1	0.867935	3.27E-05

ENSG00000104343	UBE2W	0.503851	4.69E-05
ENSG00000182247	UBE2E2	0.746942	0.000065
ENSG00000078140	UBE2K	0.277765	0.000104
ENSG00000170142	UBE2E1	0.523719	0.000327
ENSG00000156587	UBE2L6	1.059927	0.000437
ENSG00000175931	UBE2O	0.349526	0.000465
ENSG00000130725	UBE2M	0.31568	0.000899
ENSG00000107341	UBE2R2	0.236068	0.002875
ENSG00000184182	UBE2F	0.245525	0.021078
ENSG00000198833	UBE2J1	0.305753	0.02493
ENSG00000187531	SIRT7	0.5201	1.33E-10
ENSG00000177463	NR2C2	0.771918	0.000254
ENSG00000182054	IDH2	-0.83851	2.92E-14
ENSG00000138413	IDH1	-0.94364	2.62E-18
ENSG00000142208	AKT1	-0.16012	0.045604
ENSG00000082701	GSK3B	0.515329	0.000158

Differentially expressed genes ($p < 0.05$) reported in the published literature as functionally related to the top five up- and down-regulated genes in Table S2a.

Supplementary Table S3a. Top five down- and up-regulated genes in macrophages infected with *C. neoformans.*

Gene	Gene Id	log2FoldChange	*p* value
ENSG00000116750	UCHL5	-0.20727	0.04472
ENSG00000138035	PNPT1	-0.54945	1.67E-10
ENSG00000130348	QRSL1	-0.37168	3.88E-07
ENSG00000196510	ANAPC7	-0.39341	0.000302
ENSG00000131467	PSME3	-0.30045	3.30E-05
ENSG00000109107	ALDOC	1.825971589	2.32E-10
ENSG00000111674	ENO2	1.489680497	9.70E-15
ENSG00000159399	HK2	1.121945578	6.74E-16
ENSG00000102144	PGK1	0.746990585	2.14E-08
ENSG00000111640	GAPDH	0.713009241	1.06E-07

Gene identification for gene-level analysis was performed as in Table S2a.

Supplementary Table S3b. Genes functionally related with the top five down- and up-regulated genes in macrophages infected with *C. neoformans.*

Gene	Gene Id	log2FoldChange	*p* value
ENSG00000185920	PTCH1	0.761480524	0.012629752
ENSG00000127528	KLF2	0.62022683	0.047195731
ENSG00000166819	PLIN1	0.973316711	0.003288899
ENSG00000147872	PLIN2	0.692199046	0.00059194
ENSG00000149925	ALDOA	0.568357915	4.63E-06
ENSG00000111669	TPI1	0.6108752	5.94E-07
ENSG00000171314	PGAM1	0.429844596	0.001449782
ENSG00000105220	GPI	0.684479515	7.26E-09
ENSG00000067057	PFKP	0.690877949	0.000281329
ENSG00000152256	PDK1	1.070412291	8.18E-08
ENSG00000067992	PDK3	0.363881857	0.004868894
ENSG00000134333	LDHA	1.050510273	3.90E-08
ENSG00000104812	GYS1	0.794960339	3.17E-07
ENSG00000174437	ATP2A2	-0.330898147	0.029025124
ENSG00000132356	PRKAA1	-0.186245418	0.045627492
ENSG00000131828	PDHA1	-0.222577362	0.025045384
ENSG00000072210	ALDH3A2	-0.331695382	7.63E-05
ENSG00000161618	ALDH16A1	-0.281990927	0.008872148
ENSG00000184254	ALDH1A3	-0.998071894	0.011142259
ENSG00000159423	ALDH4A1	-0.573208906	0.013428796
ENSG00000137124	ALDH1B1	-0.500558706	0.026687482
ENSG00000135218	CD36	-0.435604275	0.045176474

Differentially expressed genes ($p < 0.05$) reported in the published literature as functionally related to the top five up- and down-regulated genes in Table S3a.

Supplementary Table S4. Levels of cytokines in supernatants of ACHN cell cultures and MDM treated with ACHN-conditioned culture medium.

Cytokine	ACHN (pg/ml)	ACHN-medium treated MDM (pg/ml)
IL-6	174	82
IL-8	543	4889
CCL4	137	2414
IL-1α	4	34
CXCL16	23	1898
PDGF-AA	46	37
CCL2	405	13775
FLT3L	10	22
TGF-α	6	17

The level of cytokines produced by ACHN cell cultures was calculated as average of technical duplicates for each analyte. MDM from donors (n=5) was treated with ACHN-conditioned culture medium. The average level in MDM supernatants from the five donors was calculated for each analyte.

Supplementary Table S5a. Top five down and upregulated genes in macrophages treated with ACHN-conditioned culture medium.

Gene	Gene Id	log2FoldChange	*p* value
ENSG00000109819	PPARGC1A	-1.108179567	0.0019129
ENSG00000179598	PLD6	-0.340258537	0.0313289
ENSG00000164742	ADCY1	-1.789945859	1.59E-05
ENSG00000156239	N6AMT1	-0.407562926	0.0036691
ENSG00000215009	ACSM4	-0.732880759	0.0301056
ENSG00000111674	ENO2	0.585748	0.005669
ENSG00000160883	HK3	0.417557	0.035561
ENSG00000111669	TPI1	0.280168	0.035629
ENSG00000102144	PGK1	0.263218	0.041578
ENSG00000162433	AK4	0.550335	0.00025

Gene identification for gene-level analysis was performed as in Table S2a.

Supplementary Table S5b. Genes functionally related with the top five down and upregulated genes in macrophages treated with ACHN conditioned culture medium.

Gene	Gene Id	log2FoldChange	*p* value
ENSG00000135218	CD36	-0.71398	0.002177
ENSG00000166411	IDH3A	-0.36846	0.012774
ENSG00000110090	CPT1A	-0.3713	0.048744
ENSG00000165092	ALDH1A1	-0.6716	0.005934
ENSG00000214456	PLIN5	0.763578	0.033995
ENSG00000007866	TEAD3	0.442895	0.043061
ENSG00000142583	SLC2A5	0.948899	0.001353
ENSG00000114268	PFKFB4	0.682204	3.73E-05

Differentially expressed genes ($p < 0.05$) reported in the published literature as functionally related to the top five up- and down-regulated genes in Table S5a.

Supplementary Materials

Materials and Methods

Cell cultures. Peripheral blood mononuclear cells (PBMC) were isolated and monocyte-derived macrophages (MDM) were generated as previously described (1). Briefly, human blood was obtained from the New York Blood Center (Long Island City, NY, USA), and PBMC were isolated by Ficoll density gradient centrifugation (Ficoll-Paque, GE Healthcare, Uppsala, Sweden). Isolated PBMC were washed and resuspended in serum-free RPMI-1640 medium (Corning, Manassas, VA, USA) supplemented with 4 mM L-glutamine (Corning, Manassas, VA, USA) and seeded at a density of 1×10^7 cells/ml in tissue culture multiwell plates or flasks. After 4 hr, non-adherent cells were removed by washing five times with 1× PBS (Corning, Manassas, VA, USA) and the adherent fraction was cultured over 7 days in complete RPMI medium (RPMI-1640 supplemented with 10% fetal bovine serum (Seradigm, Radnor, PA, USA) and 4 mM L-glutamine.
The ACHN cell line was obtained from the American Type Culture Collection (ATCC). To obtain conditioned medium, cells were grown in complete RPMI for 6-9 passages in humidified atmospheric air containing 5% CO_2 at 37°C. Cell culture medium was collected 24 hr after the last passage, filtered through a 0.4 μM filter to remove any cell debris, and stored in aliquots at -80°C.

IL-8 depletion of ACHN conditioned medium. To deplete IL-8 from ACHN cell culture medium, 1.5 mg of streptavidin-conjugated Dynabeads M-280 (Invitrogen, Waltham, MA, USA) were incubated with 15 μg of a biotinylated antibody against IL-8 (R&D Systems, Minneapolis, MN, USA) for 30 min at RT in rotation. Beads were washed with 1× PBS for 5 times by magnetic sedimentation and incubated with 30 ml of ACHN culture medium in rotation for 30 min at RT. After removal of the beads by magnetic sedimentation and subsequent 0.4 μM filtration, IL-8-depleted ACHN was used for monocyte/macrophage treatments. ACHN medium incubated with unconjugated Dynabeads M-280 was used to treat monocyte/macrophage as negative control. IL-8 depletion was confirmed by using an ELISA assay kit following manufacturer's instructions (Invitrogen, Waltham, MA, USA).

Pathogens. mCherry-expressing *M. tuberculosis* $H_{37}Rv$ strain was grown in liquid medium to mid-log phase and frozen stocks prepared for macrophage infection as described (5). mCherry-

expressing *C. neoformans* H99 strain was grown in YPD medium overnight at 30°C with constant agitation, washed three times with 1× PBS, and resuspended in 1× PBS before use in macrophage infection. The final fungal concentration was adjusted to 1 x 10^8 cell/ml with complete RPMI medium [RPMI-1640 supplemented with 10% heat-inactivated fetal bovine serum (Seradigm, Radnor, PA, USA) and 4 mM L-glutamine]. A suspension of heat-killed *C. neoformans* was prepared by resuspending the fungal cells in 1× PBS and incubating the suspension at 75°C for 1 hr in a heat block. To prepare cell-free culture supernatant, *C. neoformans* H99 was grown in complete RPMI medium for 24 hr (end of logarithmic growth phase), filtered through a 0.2 μM filter, and used for macrophage treatments.

Macrophage treatments. For infection experiments, following 7 days of MDM differentiation, MDM were counted and infected with either *M. tuberculosis or C. neoformans*. The pathogen inoculum for infection was prepared by adding the microorganisms to supplemented RPMI-1640 medium (as above) to obtain an MOI of 4 colony-forming units (cfu) per cell. Pathogen clumps were disrupted by vortexing with sterile 3-mm-diameter glass beads for 2 min; this suspension was used for MDM infection. MDM were incubated with *M. tuberculosis* for 24h. MDM were infected with *C. neoformans* for 3 h, washed with 1× PBS three times, and incubated with fresh medium for 24 hr. When chemical inhibitors were tested, they were added to the MDM at the time of infection. For treatment of uninfected MDMs with recombinant TNFα, the cytokine was incubated with the MDMs for 24 hr.

At 24 hr post-infection, supernatants were collected and frozen for cytokine analysis and MDM were washed with 1× PBS three times and collected for subsequent analysis.
For treatment with ACHN conditioned medium or IL-8 recombinant cytokine (Abcam, Cambridge, England), after PBMC isolation, the adherent fraction of PBMC was treated with conditioned medium (50% fresh medium and 50% conditioned medium) or IL-8 for 7 days. Medium was replaced at 3 and 6 days of incubation. When neutralizing antibody or relative isotype controls were used, antibodies were added to the cells at the same time as the conditioned medium. At day 7 post-treatment, supernatants were collected and frozen for cytokine analysis, and MDM were washed with 1× PBS three times and collected for subsequent analysis.
Antibody/cytokine/chemical inhibitor doses were selected based on available EC_{50} data and toxicity profiles with untreated macrophages (Trypan Blue staining or MTS assay; CellTiter 96 Aqueous One Solution Cell Proliferation Assay Promega, Madison, WI, USA). Only

inhibitor/antibody doses resulting in >90% cell viability were utilized. The following concentrations were used: 30 nM A922500 (DGAT inhibitor) (Santa Cruz Biotechnology, Dallas, TX, USA), 0.4 nM rapamycin (mTORC1 inhibitor) (Selleckchem, Houston, TX, USA), 0.2-6 ng/ml recombinant IL-8 (Research and Diagnostic Systems, Minneapolis, MN, USA) and 2.5-40 ng/ml recombinant TNFα (Abcam, Cambridge, England), 1 µg/ml IL-8 neutralizing antibodies (MAB208; clone #6217) and mouse IgG1 isotype control (MAB002; clone#11711) (Research and Diagnostic Systems, Minneapolis, MN, USA).

Imaging flow cytometry. Imaging flow cytometry of MDM was performed as previously described (1). MDM were detached from tissue culture plates by incubating with 5 mM EDTA in 1× PBS pH 8 for 30 min followed by gentle scraping, washed once with 1× PBS, and fixed with 4% paraformaldehyde in 1× PBS for 45 min at room temperature. Cells were then washed with 1× PBS containing 0.1% bovine serum albumin (PBS-BSA), resuspended in 50 µl of PBS-BSA containing 5 µl of Fc receptor blocking solution, FcX (BioLegend, San Diego, CA), and incubated at room temperature for 5 min. After incubation, 50 µl of PBS-BSA containing 2.5 µl of CD11c APC antibody (clone S-HCL-3; BD Biosciences) were added to each tube, and samples were incubated at 4°C for 30 min. After washing with PBS-BSA, cells were stained with 0.3 µg/ml Bodipy 493/503 (Life Technologies, Carlsbad, CA) in 1× PBS for 15 min. For each experimental condition, data from 5,000–10,000 CD11c+ cells were acquired with an ImageStream^{X}Mark II imaging flow cytometer (Amnis Corporation, Seattle, WA) using 60× magnification. Image data were analyzed by IDEAS software version 6.0 (Amnis Corporation, Seattle, WA) after applying a compensation matrix and selecting the region of interest (lipid droplets) with the Spot Mask tool. Median fluorescence intensity and spots per cell were extracted.

Lipid quantification in macrophages. MDM were detached from the cell culture plates as described above and transferred to microcentrifuge tubes. Cell pellets were obtained by culture centrifugation at 300*x g* and frozen for subsequent lipid measurement. Triglyceride-Glo™ Assay and Cholesterol/Cholesterol Ester-Glo™ Assay kits (Promega; Madison, WI, USA) were used to quantify triglycerides and cholesterol, following the manufacturer's instructions. Cholesterol was quantified following addition of cholesteryl esterase to convert cholesteryl esters to free cholesterol, following manufacturer's instructions.

Mouse infection and tissue collection. Female mice with an average weight of 20-25 g were

used. C57BL/6 mice were purchased from the Jackson Laboratories. Animal studies were performed at the Public Health Research Institute Animal facility. All studies were conducted following biosafety level 2 (BSL-2) protocols and procedures approved by the Institutional Animal Care and Use Committee (IACUC) and Institutional Biosafety Committee of Rutgers University under protocol 999901066. *C. neoformans* wild type strain H99 was cultured on YPD medium. To prepare fungal cells for infection, an overnight culture of *C. neoformans* H99 was washed three times with 1× PBS and the concentration of yeast cells was determined by hemocytometer counting. The final fungal concentration was adjusted with PBS to 2 x 10^6 cell/ml. Each mouse was infected intranasally with 1 x 10^5 H99 cells in a 50 μl volume after being anesthetized with a mix of Ketamine (12.5 mg/mL) and Xylazine (1 mg/mL). After infection, animals were weighed daily and monitored twice daily for progression of disease, including weight loss, gait changes, labored breathing, and fur ruffling. Infected animals were sacrificed at day 7 post-infection, according to the experimental design and the Rutgers University IACUC approved animal protocol. A part of the dissected lung tissues was fixed in 10% formalin solution and sent to the Rutgers histopathological core facility for section preparation and staining with hematoxylin and eosin (H&E) using standard protocols. Another portion of the lung tissues was placed on labeled plastic cryohistology trays and frozen on dry ice for 2 minutes in a Styrofoam container. The samples were then wrapped with aluminum foil and stored in labeled Ziploc bags at -80°C until use for MALDI imaging.

Human Biopsies. Papillary renal cell carcinoma tissue was obtained from the Rutgers CINJ Biorepository. Tissue collected during surgery was frozen by the Biorepository using the PrestoCHILL machine, which allows for ultrafast freezing. The tissue analyzed derived from a radical nephrectomy; the RWJUH surgical pathology report indicated that the tumor was Type 1 and WHO histologic grade G2.

Matrix-assisted laser desorption/ionization (MALDI) mass spectrometry. Frozen lung samples were used to prepare 12 μm-thick cryosections using a Leica CM1860 cryostat. Tissue sections were mounted onto indium tin oxide coated glass slides (Delta Technologies Limited, Loveland, Colorado) and stored individually in Ziploc bags at -80°C. Adjacent sections were prepared on Superfrost Plus™ glass microscope slides (Fisherbrand) for H&E staining, which was performed using standard protocols at the UTMB Histology core. Sections for MALDI imaging were coated with 2′,4′,6′-Trihydroxyacetophenone monohydrate at 10 mg/ml in 50:50 cyclohexane/methanol using a HTX TM Sprayer. 20 passes were performed over each tissue at

a spray volume of 50 ml/min and nozzle temperature of 50°C. Once sprayed, samples were placed individually in Ziploc bags and immediately transferred to a -80°C freezer for storage. Following MALDI-MS image acquisition, matrix was removed from the slides by serial rinses in 50% ethanol, and tissue sections were allowed to airdry at room temperature for 30 min. The tissue sections were then transferred to the UTMB histology core for H&E and Oil Red staining using standard protocols.

MALDI MS imaging was performed using a Q-Exactive HF mass spectrometer (Thermo Scientific, Bremen, DE) fitted with a MALDI/ESI Injector (Spectroglyph LLC, Kennewick, WA). Laser post-ionization (MALDI-2) was used to enhance analytical sensitivity for triglycerides and cholesteryl esters. Images were acquired at 20 micrometer voxel size, using a pulse energy of ~6 mJ and repetition rate of 30Hz. Q Exactive HF MS Scan parameters were optimized for triglyceride and cholesteryl ester detection: polarity – positive, scan range – 350-1500 m/z, resolution – 120,000, automatic gain control - off, maximum inject time 250 ms**.** ImageInsight™ (Spectroglyph LLC) software was used for initial data visualization and to convert data files into imzML format for visualization and further processing in SCiLS™ software (Bruker, Billerica, MA). TAG and CE species were predominantly detected as potassium adducts and lipid identifications were assigned within a 3pm mass tolerance range using Lipid Maps database. All lipid images produced were normalized to the total ion chromatogram.

Cytokine measurement. A panel of cytokines/chemokines/growth factors was measured using a 43-Plex Luminex technology at the Rutgers Immune Monitoring and Advanced Genomics Core Facility (TNF-α, IL-6, PDGF-BB, IL-3, IL-8, CXCL10/IP-10, IL-10, EGF, IL-1β, IFN-ɣ, IL-1ra/IL-1F3, IL-12 p70, CCL3, CCL22/MDC, CCL4/MIP-1β, IL-1α/IL-1F1, CXCL16, IL-17/IL-17a, IL-2, TNF-β, GM-CSF, FGFbasic, M-CSF, CXCL9, IL-5, G-CSF, PDGF-AA, CCL2/JE/MCP-1, IFN-α, IL-13, Chemerin, CD40L, FLT3L, IL-18, IL-27, VEGF, IL-7, IL-17E/IL-25, CCL8/MCP-2, IL-4, IL-15, TGF-α, IL-12/IL-23 P40. Assays were performed in microtiter plates and plates were read with the Luminex 200 instrument. Each sample was measured in duplicate.

Transcriptomics and pathway analysis.

RNA was isolated by using TRI reagent (Molecular Research Center, Cincinnati, OH, USA) according to manufacturer's instructions. RNA-Seq was conducted at the Rutgers Genomics Center. The quality of RNA was first assessed for integrity on an Agilent TapeStation using high sensitivity RNA kit, PN-5067-5579 (Agilent technologies Inc, CA). Samples with RNA integrity number (RIN) >7.0 were considered to have sufficient quality for subsequent processing.

Illumina compatible cDNA libraries generated for polyA selected mRNA using NEB next ultra RNA library preparation kit Cat#E7530L (New England Biolabs Inc, MA). The cDNA libraries were purified using AmpureXP beads, Product No: A63882 (Beckman Coulter) and analyzed on an Agilent TapeStation using HS D1000 screen tape PN- 5067-5584 (Agilent Technologies Inc, CA) to estimate the size of the library and quantitated using Qubit 4 Fluorometer, using HS reagent kit, Cat. No. Q33231 (Thermofisher Scientific, MA). Equimolar amounts of barcoded libraries pooled together and sequenced on Illumina NovaSeq 6000 Instrument (Illumina, San Diego, CA) using SP flow cells kits (cat # 20040326) with 2x150 cycles configuration.
Read count and gene-level transcript abundance analysis were performed by the Rutgers Molecular and Genomics Informatics Core. In brief, raw transcriptome reads were assessed for quality control (FASTQC v0.11.8) and trimmed for quality and adapter contaminant (cutadapt v 2.5). Trimmed reads were aligned to the mouse genome (GRCh37) using STAR (v2.6.1), followed by transcript abundance calculation and hit count extraction with StringTie (v2.0) and featureCounts (v1.6.4), respectively.
The differential expression between sample classes (treated vs non treated for each condition) was tested with coincident extreme ranks in numerical observations (CERNO) (2) for metabolism-related Gene Ontology gene sets. The Benjamini–Hochberg method was used to calculate the false discovery rate (FDR). Gene sets (pathways) were identified at a cutoff false discovery rate of 0.05. Genes in differentially expressed metabolism-related Gene Ontology gene sets having FDR < 0.05 were selected based on individual *p* value ($p << 0.05$) and ranked by log2 fold change (treated vs non-treated in each condition) and number of occurrences in the differentially expressed gene sets. The top five down- and up-regulated genes were selected for further analysis. Differentially expressed genes ($p < 0.05$) reported in the published literature as functionally related to the top five up- and down-regulated genes in each condition were also selected for analysis and data interpretation.

Supplementary text on transcriptomics analyses

1. Metabolism-related gene-level analysis of *M. tuberculosis*-infected monocyte-derived macrophages.

Genes in differentially expressed metabolism-related Gene Ontology gene sets having FDR < 0.05 were selected based on individual *p* value (*p* << 0.05) and ranked by log2 fold change (infected vs non-infected) and number of occurrences in the differentially expressed gene sets. We analyzed the top five down- and up-regulated genes (**Table S2a**) and differentially expressed genes (*p* < 0.05) that were functionally related to them (**Table S2b**). In the text below, and in the subsequent sections, the names of the top five up- and down-regulated genes (**Table S2a**) are shown in bold. The names of the up- and down-regulated functionally related genes (**Table S2b**) are underlined.

Multiple mechanisms of decreased fatty acid oxidation were identified. These included downregulation of genes involved in (i) synthesis of acyl-CoA (acyl-CoA synthases ACSM4, **ACSM5**, ACSBG2), (ii) acyl-CoA conversion to acyl-carnitine [(carnitine transferase in peroxisome (**CROT**) and mitochondria (CPT1A, CPT2)], which is required for transport across the mitochondrial membrane (3-5), and (iii) oxidation to fatty acids of fatty aldehydes and fatty alcohols derived from lipid catabolism (fatty aldehyde dehydrogenase, **ALDH3A2**) (6-8).

Gene expression markers of increased production of triacylglycerols (TAG) were also identified. One set of gene expression changes were associated with increased production of ceramide, which induces triglyceride production (9-12). These included (i) downregulation of the aldehyde hydrogenase **ALDH3A2**, which converts hexadecenal to hexadecenoic acid. The resulting hexadecenal accumulation may lead to ceramide production; (ii) downregulation of ceramidase ACER2, and (iii) upregulation of ceramide synthases (CERS3 and CERS5). An additional marker of triglyceride accumulation was the downregulation of the gene encoding palmitoyl-protein thioesterase 1 (**PPT1**), which expresses lipolytic activity (13, 14). TAG accumulation in *M. tuberculosis*-infected macrophages may also result from altered cellular redox. For example, we observed downregulation of genes encoding glutathione S-Transferase Mu (GSTM1, GSTM2, **GSTM4**), a scavenger of reactive oxygen species (ROS), which can induce lipid droplet formation by inducing the ASK1/p38/JNK pathway (15-17).

As mentioned in the main text, *M. tuberculosis*-infected macrophages exhibited downregulated TP53 gene and upregulated TP53-specific E3 ligases (FBXW7, MDM2) that target this factor for proteasomal degradation (18-20). Upregulation of XBP1 can also lead to decreased TP53 levels by inducing MDM2 (21). Reduced TP53 activity well correlates with downregulation of TP53-dependent Deptor, which negatively regulates mTOR (22, 23). mTORC1 signaling is lipogenic in multiple ways, including increased de novo lipid synthesis, reduced lipolysis, and reduced fatty acid oxidation (24, 25).

We also observed upregulation of metabolism-related ubiquitination processes, which was prominent both at the pathway- arufnd at the gene-level (**Fig. S4** and **Table S2a**). At the gene level, the top five upregulated genes featured genes encoding E3 ligases (**ZNRF1**) and associated proteins (**FZR1**, **FBXL20**, **UBE2H**) and the deubiquitinase **USP7,** which increases ZNRF1 activity as well as MDM2 activity (26-31). These changes presumably result in substantial macrophage proteome remodeling in response to *M. tuberculosis* infection. In particular, they may affect a variety of lipid metabolism regulators, including AKT1 and PLK1 (32, 33). **USP7** also stabilizes sirtuins (e.g., SIRT7, which is also upregulated at the gene level), resulting in increased expression of factors associated with TAG synthesis (26, 34).

2. Metabolism-related gene-level analysis of *C. neoformans*-infected monocyte-derived macrophages.

The top five up- and down-regulated genes (**Table S3a**) and functionally related, differentially expressed genes (**Table S3b**) were determined as described above for *M. tuberculosis* infection.

The top five upregulated genes encode glycolytic enzymes, strongly indicating a prominent role of a switch to glycolysis in *C. neoformans*-infected macrophages, which leads to increased triglyceride biosynthesis in multiple ways. Increased expression of hexokinase 2 (**HK2**) and other glycolytic enzymes (GPI, PFKP) results in increased production of fructose 1,6-bisphosphate, which can be converted to dihydroxyacetone phosphate (DHAP) and glyceraldehyde 3-phosphate (G3P), due to the increased production of aldolases (**ALDOC**, ALDOA). Also, the upregulation of the triosephosphate isomerase-encoding gene (TPI) might lead to increased production of DHAP, which is a precursor for triglyceride biosynthesis. The upregulation of additional glycolytic genes (**GAPDH**, **PGK1**, PGAM1, **ENO2**) might result in increased synthesis of phosphoenolpyruvate (PEP) (35). Increased PEP levels might inhibit

sarco/endoplasmic reticulum Ca2+-ATPase, resulting in calcium imbalance and endoplasmic reticulum stress, which ultimately lead to lipogenesis (36-38). Moreover, the products of glycolytic genes may favor lipid accumulation in ways other than their direct role in glycolysis. For example, the **HK2** product participates in the metabolic switch from oxidative phosphorylation to glycolysis (39) and inhibits lipases (40). In addition, phosphoglycerate kinase 1 (**PGK1**) can phosphorylate pyruvate dehydrogenase kinases, which are also upregulated (PDK1, PDK3), and inhibit the pyruvate dehydrogenase (PDH) complex (41). Inhibition of the PDH Complex, further compounded by the downregulation of one of its subunit-encoding genes (PDHA1), leads to impaired tricarboxylic acid cycle and enhanced glycolysis (42). As a result, pyruvate may accumulate and converted to lactate by lactate dehydrogenase (LDHA) (43), which we find upregulated. Lactate may inhibit lipolysis, thus resulting in lipid accumulation (44).

As mentioned in the main text, we also found indicators of reduced mitochondrial functions in *C. neoformans*-infected macrophages, including downregulation of polyribonucleotide nucleotidyl transferase 1 (**PNPT1**) and a glutaminyl-tRNA amidotransferase subunit 1 (**QRSL1**). The **PNPT1** product regulates the expression of the electron transport chain components at the mRNA and protein levels. Thus, downregulation of this gene might result in reduced function of the respiratory chain and decreased oxidative phosphorylation, leading to lipid accumulation (45, 46). The **QRSL1** product enables glutaminyl-tRNA synthase (glutamine-hydrolyzing) activity in the mitochondria. Missense mutations in the human QRSL1 locus have been associated with defects in mitochondrial respiratory chain complex and oxidative phosphorylation (47, 48). The resulting oxidative stress enhances lipogenesis (49, 50).

Lipid accumulation in *C. neoformans*-infected macrophages may also result from dysregulation of various other cellular activities. First, infected cells may exhibit increased fatty acid synthesis and decreased fatty acid oxidation due to the downregulation of (i) AMP-activated protein kinase (AMPK/PRKAA1), which inhibits de novo biosynthesis of fatty acids, increases fatty acid uptake, and stimulates fatty acid oxidation (51-53) and (ii) multiple genes encoding aldehyde dehydrogenases (ALDH3A2, ALDH16A1, ALDH1A3, ALDH4A1, ALDH1B1), which are involved in fatty acid oxidation (8). Second, perilipin genes (PLIN1, PLIN2), which are structural components of lipid droplets (54), are upregulated. PLIN gene upregulation may be associated, at least in part, with the downregulation of proteasome activator subunit 3 (**PSME3**) (55). The PSME3 product induces the degradation of Kruppel Like Factor 2 (KLF2) (56). Since KLF2 promotes the transcription of TAG synthesis and lipid storage genes, including PLIN1 and PLIN2 (57), increased KLF2 activity secondary to PSME3 downregulation is associated with lipid

accumulation (58). Third, one may hypothesize that another system involved in *C. neoformans*-infection-induced lipid accumulation is Hedgehog signaling. Inhibition of Hedgehog signaling results in fat accumulation (59, 60). Downregulation of ubiquitin C-terminal hydrolase L5 (**UCHL5**), which positively regulates Hedgehog signaling by deubiquitinating one of its components, the Smoothened (SMO) protein (61, 62), might result in reduced Hedgehog signaling and increased lipid accumulation. The observed upregulation of patched1 (PTCH1), which represses SMO (63, 64), is consistent with a role of Hedgehog in lipid accumulation in *C. neoformans*-infected macrophages. Fourth, the downregulation of anaphase promoting complex subunit 7 (**ANAPC7**) may also lead to triglyceride accumulation. ANAPC7, which is a component of the anaphase promoting complex/cyclosome (APC/C) (65), interacts with the CBP/p300 coactivator and stimulates its acetyl transferase activity (66). Among the CBP/p300 targets is farnesoid X receptor (FXR) (67). Acetylation of FXR leads to protein stabilization but reduces its activity as transcriptional regulator (68). Thus, reduced FXR acetylation resulting from ANAPC7 downregulation might activate FXR signaling. FXR signaling may induce lipid accumulation and reduced fatty acid oxidation (69-71). However, some reports show the opposite effect of FXR signaling on lipid accumulation (72, 73), suggesting perhaps context-specific effects.

3. Metabolism-related gene-level analysis of monocyte-derived macrophages treated with ACHN culture medium.

The top five up- and down-regulated genes (**Table S5a**) and functionally related, differentially expressed genes (**Table S5b**) were determined as described above for *M. tuberculosis* infection.

The top upregulated genes are enriched for glycolytic genes. Increased expression of hexokinase 3 (**HK3**) and other glycolytic genes (PFKFB4) might result in increased production of fructose 1,6-bisphosphate, which breaks down into dihydroxyacetone phosphate (DHAP) and glyceraldehyde 3-phosphate (G3P) (39, 74). Upregulation of triosephosphate isomerase (**TPI1**) also contributes to the conversion of DHAP to G3P, favoring triglyceride synthesis (75). G3P formation can also lead to synthesis of phosphoenolpyruvate (PEP), due to upregulation of other glycolytic genes (**PGK1**, **ENO2**) (35). Accumulation of PEP may result in enhanced production of pyruvate. Reduced utilization of pyruvate in the TCA cycle, which may be downregulated (see below), might lead to enhanced lipogenesis (36).

Macrophages treated with ACHN medium also exhibit transcriptional markers of reduced production/activity of the energy sensor AMP-activated protein kinase (AMPK). One is the upregulation of adenylate kinase 4 (**AK4**), which regulates cellular ATP levels and inhibits AMPK signaling (76). Another is the downregulation of phospholipase D family member 6 (**PLD6**), which activates AMPK by altering mitochondrial fusion and fission dynamics (77). The resulting decreased activity of AMPK, an energy sensor maintaining energy homeostasis (78), might result in increased YAP/TAZ signaling, which regulates metastasis and metabolic reprogramming in cancer cells (79), since AMPK inhibits YAP/TAZ via YAP phosphorylation (80). This scenario is suggested by the observed upregulation of genes associated with or regulated by YAP/TAZ, such as TEAD3, SLC2A5, and PLIN5, in ACHN-medium-treated macrophages. YAP/TAZ interacts with the TEAD transcription factors (81) to induce various target genes. One is the fructose transporter SLC2A5; its upregulation might result in increased fructose uptake, leading to lipogenesis via fructolysis (82, 83). YAP/TAZ also induces PLIN5, a member of a family of lipid-droplet-associated proteins (84), which protects triglycerides from enzymatic degradation by inhibiting adipose triglyceride lipase (85, 86). More in general, YAP/TAZ signaling and lipid metabolism are intricately intertwined in cancer cells, and YAP/TAZ is involved in the metabolism of fatty acids and sterols (79)**,** suggesting a role of this signaling pathway also in the cholesterol accumulation detected in the ACHN-medium-treated cells.

We identified additional gene expression markers of lipid accumulation. Impaired fatty acid oxidation, which may result in lipid accumulation, is suggested by the downregulation of the acyl-CoA synthase **ACSM4** and functionally related genes, such as acyl-CoA conversion to acyl-carnitine (carnitine palmitoyl transferase I, CPT1) and oxidation of fatty aldehydes to fatty acids (aldehyde hydrogenases, ALDH genes) (3-5, 8). The downregulation of **N6AMT1**, which encodes a methyltransferase mostly implicated in DNA and protein modification, is also consistent with lipid accumulation, since this gene product has been associated with lipid catabolism and low triglyceride levels (87, 88) by yet unknown mechanisms.

One of the top five downregulated genes is peroxisome proliferator-activated receptor gamma coactivator 1-alpha (**PPARGC1a**), a transcriptional coactivator that regulates cellular metabolism (89). Downregulation of PPARGC1a is consistent with the observed downregulation of two of its target genes, NAD-dependent isocitrate dehydrogenase 3 catalytic subunit alpha (IDH3A) (90) and CD36. Isocitrate dehydrogenases catalyze the oxidative decarboxylation of isocitrate to 2-oxoglutarate, which is a rate-limiting step of the TCA cycle. Thus, IDH3A downregulation might result in TCA cycle disruption. An additional indicator of dampened TCA

cycle is the above-mentioned upregulation of **AK4**, since this adenylate kinase is associated with decreased expression of key TCA cycle genes (91). The scavenger receptor CD36 is a critical fatty acid sensor and regulator of lipid metabolism (92, 93). CD36 mediates fatty acid uptake, increases cholesterol efflux, and favors proteasomal degradation of HMG-CoA reductase, the rate-limiting enzyme in sterol synthesis (94, 95). CD36 downregulation might result in cholesterol accumulation, reduced fatty acid oxidation and mitochondrial function, and increased fatty acid storage (94). Thus, CD36 downregulation is consistent with increased accumulation of both cholesterol and triglycerides in ACHN-medium-treated cells. The decreased activity of AMPK proposed above would further decrease CD36 function, since AMPK promotes CD36 expression and translocation (52).

An additional marker of dysregulated cholesterol homeostasis is the downregulation of adenylate cyclase (**ADCY1**). In response to G-protein signaling, adenylate cyclases are activated and generate cyclic AMP (cAMP) (96). cAMP participates in various signal transduction mechanisms regulating adipogenesis, lipolysis, and cholesterol efflux (96, 97). Reduced cAMP production may be associated with decreased cholesterol efflux and decreased lipolysis (96-99).

Supplementary References

1. V. Guerrini *et al.*, Storage lipid studies in tuberculosis reveal that foam cell biogenesis is disease-specific. *PLoS Pathog* **14**, e1007223 (2018).
2. E. Croze, K. D. Yamaguchi, V. Knappertz, A. T. Reder, H. Salamon, Interferon-beta-1b-induced short- and long-term signatures of treatment activity in multiple sclerosis. *Pharmacogenomics J* 10.1038/tpj.2012.27 (2012).
3. A. Rinaldi *et al.*, Impaired fatty acid metabolism perpetuates lipotoxicity along the transition to chronic kidney injury. *JCI Insight* **7** (2022).
4. K. R. Dunning, M. R. Anastasi, V. J. Zhang, D. L. Russell, R. L. Robker, Regulation of fatty acid oxidation in mouse cumulus-oocyte complexes during maturation and modulation by PPAR agonists. *PLoS One* **9**, e87327 (2014).
5. F. Le Borgne, A. Ben Mohamed, M. Logerot, E. Garnier, J. Demarquoy, Changes in carnitine octanoyltransferase activity induce alteration in fatty acid metabolism. *Biochem Biophys Res Commun* **409**, 699-704 (2011).
6. W. B. Rizzo, Fatty aldehyde and fatty alcohol metabolism: review and importance for epidermal structure and function. *Biochim Biophys Acta* **1841**, 377-389 (2014).
7. W. B. Rizzo, Sjogren-Larsson syndrome: molecular genetics and biochemical pathogenesis of fatty aldehyde dehydrogenase deficiency. *Mol Genet Metab* **90**, 1-9 (2007).
8. S. Li, Z. Yang, H. Zhang, M. Peng, H. Ma, Potential role of ALDH3A2 on the lipid and glucose metabolism regulated by (-)-hydroxycitric acid in chicken embryos. *Anim Sci J* **90**, 961-976 (2019).
9. S. A. Summers, Ceramides: Nutrient Signals that Drive Hepatosteatosis. *J Lipid Atheroscler* **9**, 50-65 (2020).
10. K. Nakahara *et al.*, The Sjogren-Larsson syndrome gene encodes a hexadecenal dehydrogenase of the sphingosine 1-phosphate degradation pathway. *Mol Cell* **46**, 461-471 (2012).
11. M. Bektas *et al.*, Sphingosine 1-phosphate lyase deficiency disrupts lipid homeostasis in liver. *J Biol Chem* **285**, 10880-10889 (2010).
12. A. Maharaj *et al.*, Isolated glucocorticoid deficiency: Genetic causes and animal models. *J Steroid Biochem Mol Biol* **189**, 73-80 (2019).
13. R. Wang *et al.*, Identification of palmitoyl protein thioesterase 1 in human THP1 monocytes and macrophages and characterization of unique biochemical activities for this enzyme. *Biochemistry* **52**, 7559-7574 (2013).

14. A. Khaibullina *et al.*, In a model of Batten disease, palmitoyl protein thioesterase-1 deficiency is associated with brown adipose tissue and thermoregulation abnormalities. *PLoS One* **7**, e48733 (2012).
15. Y. Jin *et al.*, Glutathione S-transferase Mu 2 inhibits hepatic steatosis via ASK1 suppression. *Commun Biol* **5**, 326 (2022).
16. R. Zhuo *et al.*, Targeting Glutathione S-transferase M4 in Ewing sarcoma. *Front Pediatr* **2**, 83 (2014).
17. X. F. Leong, Lipid Oxidation Products on Inflammation-Mediated Hypertension and Atherosclerosis: A Mini Review. *Front Nutr* **8**, 717740 (2021).
18. D. Cui *et al.*, FBXW7 Confers Radiation Survival by Targeting p53 for Degradation. *Cell Rep* **30**, 497-509 e494 (2020).
19. J. Tang *et al.*, Critical role for Daxx in regulating Mdm2. *Nat Cell Biol* **8**, 855-862 (2006).
20. R. U. Janicke, D. Sohn, K. Schulze-Osthoff, The dark side of a tumor suppressor: anti-apoptotic p53. *Cell Death Differ* **15**, 959-976 (2008).
21. C. Huang *et al.*, Identification of XBP1-u as a novel regulator of the MDM2/p53 axis using an shRNA library. *Sci Adv* **3**, e1701383 (2017).
22. H. Chen *et al.*, DEP domain-containing mTOR-interacting protein suppresses lipogenesis and ameliorates hepatic steatosis and acute-on-chronic liver injury in alcoholic liver disease. *Hepatology* **68**, 496-514 (2018).
23. V. Catena, M. Fanciulli, Deptor: not only a mTOR inhibitor. *J Exp Clin Cancer Res* **36**, 12 (2017).
24. G. A. Soliman, The integral role of mTOR in lipid metabolism. *Cell Cycle* **10**, 861-862 (2011).
25. M. Laplante, D. M. Sabatini, An emerging role of mTOR in lipid biosynthesis. *Curr Biol* **19**, R1046-1052 (2009).
26. S. Ruf *et al.*, PLK1 (polo like kinase 1) inhibits MTOR complex 1 and promotes autophagy. *Autophagy* **13**, 486-505 (2017).
27. S. Zhu *et al.*, Regulation of Glucose, Fatty Acid and Amino Acid Metabolism by Ubiquitination and SUMOylation for Cancer Progression. *Front Cell Dev Biol* **10**, 849625 (2022).
28. S. Lambhate, D. Bhattacharjee, N. Jain, APC/C CDH1 ubiquitinates IDH2 contributing to ROS increase in mitosis. *Cell Signal* **86**, 110087 (2021).
29. X. Qiao, L. Zhang, A. M. Gamper, T. Fujita, Y. Wan, APC/C-Cdh1: from cell cycle to cellular differentiation and genomic integrity. *Cell Cycle* **9**, 3904-3912 (2010).

30. M. Li, C. L. Brooks, N. Kon, W. Gu, A dynamic role of HAUSP in the p53-Mdm2 pathway. *Mol Cell* **13**, 879-886 (2004).
31. W. Ni *et al.*, USP7 mediates pathological hepatic de novo lipogenesis through promoting stabilization and transcription of ZNF638. *Cell Death Dis* **11**, 843 (2020).
32. R. Miao *et al.*, Akt: A Potential Drug Target for Metabolic Syndrome. *Front Physiol* **13**, 822333 (2022).
33. Z. Zhang *et al.*, Plk1 inhibition enhances the efficacy of androgen signaling blockade in castration-resistant prostate cancer. *Cancer Res* **74**, 6635-6647 (2014).
34. L. Jiang *et al.*, Ubiquitin-specific peptidase 7 (USP7)-mediated deubiquitination of the histone deacetylase SIRT7 regulates gluconeogenesis. *J Biol Chem* **292**, 13296-13311 (2017).
35. M. Yang *et al.*, Hypoxia-induced MIF induces dysregulation of lipid metabolism in Hep2 laryngocarcinoma through the IL-6/JAK-STAT pathway. *Lipids Health Dis* **21**, 82 (2022).
36. P. C. Ho *et al.*, Phosphoenolpyruvate Is a Metabolic Checkpoint of Anti-tumor T Cell Responses. *Cell* **162**, 1217-1228 (2015).
37. J. Bi *et al.*, Seipin promotes adipose tissue fat storage through the ER Ca(2)(+)-ATPase SERCA. *Cell Metab* **19**, 861-871 (2014).
38. S. Fu *et al.*, Aberrant lipid metabolism disrupts calcium homeostasis causing liver endoplasmic reticulum stress in obesity. *Nature* **473**, 528-531 (2011).
39. L. H. Fairley *et al.*, Mitochondrial control of microglial phagocytosis by the translocator protein and hexokinase 2 in Alzheimer's disease. *Proc Natl Acad Sci U S A* **120**, e2209177120 (2023).
40. L. Leng *et al.*, Microglial hexokinase 2 deficiency increases ATP generation through lipid metabolism leading to β-amyloid clearance. *Nat Metab* **4**, 1287-1305 (2022).
41. X. Li *et al.*, Mitochondria-Translocated PGK1 Functions as a Protein Kinase to Coordinate Glycolysis and the TCA Cycle in Tumorigenesis. *Mol Cell* **61**, 705-719 (2016).
42. L. Xu *et al.*, SIRT3 elicited an anti-Warburg effect through HIF1alpha/PDK1/PDHA1 to inhibit cholangiocarcinoma tumorigenesis. *Cancer Med* **8**, 2380-2391 (2019).
43. S. Chen, L. Zhou, J. Sun, Y. Qu, M. Chen, The Role of cAMP-PKA Pathway in Lactate-Induced Intramuscular Triglyceride Accumulation and Mitochondria Content Increase in Mice. *Front Physiol* **12**, 709135 (2021).
44. C. Liu *et al.*, Lactate inhibits lipolysis in fat cells through activation of an orphan G-protein-coupled receptor, GPR81. *J Biol Chem* **284**, 2811-2822 (2009).

45. V. Vedrenne *et al.*, Mutation in PNPT1, which encodes a polyribonucleotide nucleotidyltransferase, impairs RNA import into mitochondria and causes respiratory-chain deficiency. *Am J Hum Genet* **91**, 912-918 (2012).
46. G. Wang *et al.*, PNPASE regulates RNA import into mitochondria. *Cell* **142**, 456-467 (2010).
47. M. Kohda *et al.*, A Comprehensive Genomic Analysis Reveals the Genetic Landscape of Mitochondrial Respiratory Chain Complex Deficiencies. *PLoS Genet* **12**, e1005679 (2016).
48. F. Dursun *et al.*, Primary adrenal insufficiency in a patient with biallelic QRSL1 mutations. *Eur J Endocrinol* **187**, K27-K32 (2022).
49. K. Puri *et al.*, Diabetes Mellitus Severity and a Switch From Using Lipoprotein Lipase to Adipose-Derived Fatty Acid Results in a Cardiac Metabolic Signature That Embraces Cell Death. *J Am Heart Assoc* **8**, e014022 (2019).
50. D. Hathazi *et al.*, Metabolic shift underlies recovery in reversible infantile respiratory chain deficiency. *EMBO J* **39**, e105364 (2020).
51. M. Foretz, P. C. Even, B. Viollet, AMPK Activation Reduces Hepatic Lipid Content by Increasing Fat Oxidation In Vivo. *Int J Mol Sci* **19** (2018).
52. W. Wu *et al.*, AMPK facilitates intestinal long-chain fatty acid uptake by manipulating CD36 expression and translocation. *FASEB J* **34**, 4852-4869 (2020).
53. H. Wu *et al.*, Lipid droplet-dependent fatty acid metabolism controls the immune suppressive phenotype of tumor-associated macrophages. *EMBO Mol Med* **11**, e10698 (2019).
54. D. L. Brasaemle, Thematic review series: adipocyte biology. The perilipin family of structural lipid droplet proteins: stabilization of lipid droplets and control of lipolysis. *J Lipid Res* **48**, 2547-2559 (2007).
55. L. C. Lin, A. C. Gao, C. H. Lai, J. T. Hsieh, H. Lin, Induction of neuroendocrine differentiation in castration resistant prostate cancer cells by adipocyte differentiation-related protein (ADRP) delivered by exosomes. *Cancer Lett* **391**, 74-82 (2017).
56. J. Sun *et al.*, The 11S Proteasome Subunit PSME3 Is a Positive Feedforward Regulator of NF-kappaB and Important for Host Defense against Bacterial Pathogens. *Cell Rep* **14**, 737-749 (2016).
57. V. G. Yerra, K. Drosatos, Specificity Proteins (SP) and Krüppel-like Factors (KLF) in Liver Physiology and Pathology. *Int J Mol Sci* **24** (2023).
58. J. Ling *et al.*, Defective lipid metabolism associated with mutation in klf-2 and klf-3: important roles of essential dietary salts in fat storage. *Nutr Metab (Lond)* **14**, 22 (2017).
59. M. Matz-Soja *et al.*, Hedgehog signaling is a potent regulator of liver lipid metabolism and reveals a GLI-code associated with steatosis. *Elife* **5** (2016).

60. G. D. Dalton *et al.*, Hepatocyte activity of the cholesterol sensor smoothened regulates cholesterol and bile acid homeostasis in mice. *iScience* **24**, 103089 (2021).
61. Z. Zhou *et al.*, The deubiquitinase UCHL5/UCH37 positively regulates Hedgehog signaling by deubiquitinating Smoothened. *J Mol Cell Biol* **10**, 243-257 (2018).
62. R. Tu *et al.*, The emerging role of deubiquitylating enzymes as therapeutic targets in cancer metabolism. *Cancer Cell Int* **22**, 130 (2022).
63. K. Petrov, T. de Almeida Magalhaes, A. Salic, Mechanism and ultrasensitivity in Hedgehog signaling revealed by Patched1 disease mutations. *Proc Natl Acad Sci U S A* **118** (2021).
64. U. Styrkarsdottir *et al.*, Sequence variants in the PTCH1 gene associate with spine bone mineral density and osteoporotic fractures. *Nat Commun* **7**, 10129 (2016).
65. J. M. Peters, The anaphase-promoting complex: proteolysis in mitosis and beyond. *Mol Cell* **9**, 931-943 (2002).
66. J. Liu, S. Y. Fuchs, Cross-talk between APC/C and CBP/p300. *Cancer Biol Ther* **5**, 760-762 (2006).
67. S. Fang *et al.*, The p300 acetylase is critical for ligand-activated farnesoid X receptor (FXR) induction of SHP. *J Biol Chem* **283**, 35086-35095 (2008).
68. J. K. Kemper *et al.*, FXR acetylation is normally dynamically regulated by p300 and SIRT1 but constitutively elevated in metabolic disease states. *Cell Metab* **10**, 392-404 (2009).
69. K. H. Kim *et al.*, Hepatic FXR/SHP axis modulates systemic glucose and fatty acid homeostasis in aged mice. *Hepatology* **66**, 498-509 (2017).
70. K. Boulias *et al.*, Regulation of hepatic metabolic pathways by the orphan nuclear receptor SHP. *EMBO J* **24**, 2624-2633 (2005).
71. M. Abdelkarim *et al.*, The farnesoid X receptor regulates adipocyte differentiation and function by promoting peroxisome proliferator-activated receptor-gamma and interfering with the Wnt/beta-catenin pathways. *J Biol Chem* **285**, 36759-36767 (2010).
72. Y. Jiao, Y. Lu, X. Y. Li, Farnesoid X receptor: a master regulator of hepatic triglyceride and glucose homeostasis. *Acta Pharmacol Sin* **36**, 44-50 (2015).
73. G. Y. Wu *et al.*, MicroRNA-122 Inhibits Lipid Droplet Formation and Hepatic Triglyceride Accumulation via Yin Yang 1. *Cell Physiol Biochem* **44**, 1651-1664 (2017).
74. W. Xu *et al.*, Hexokinase 3 dysfunction promotes tumorigenesis and immune escape by upregulating monocyte/macrophage infiltration into the clear cell renal cell carcinoma microenvironment. *Int J Biol Sci* **17**, 2205-2222 (2021).
75. G. A. Rao, M. F. Sorrels, R. Reiser, Biosynthesis of triglycerides from triose phosphates by microsomes of intestinal mucosa. *Lipids* **5**, 762-764 (1970).

76. N. J. Lanning *et al.*, A mitochondrial RNAi screen defines cellular bioenergetic determinants and identifies an adenylate kinase as a key regulator of ATP levels. *Cell Rep* **7**, 907-917 (2014).
77. B. von Eyss *et al.*, A MYC-Driven Change in Mitochondrial Dynamics Limits YAP/TAZ Function in Mammary Epithelial Cells and Breast Cancer. *Cancer Cell* **28**, 743-757 (2015).
78. G. R. Steinberg, D. G. Hardie, New insights into activation and function of the AMPK. *Nat Rev Mol Cell Biol* **24**, 255-272 (2023).
79. H. Yamaguchi, G. M. Taouk, A Potential Role of YAP/TAZ in the Interplay Between Metastasis and Metabolic Alterations. *Front Oncol* **10**, 928 (2020).
80. J. S. Mo *et al.*, Cellular energy stress induces AMPK-mediated regulation of YAP and the Hippo pathway. *Nat Cell Biol* **17**, 500-510 (2015).
81. I. M. Moya, G. Halder, Hippo-YAP/TAZ signalling in organ regeneration and regenerative medicine. *Nat Rev Mol Cell Biol* **20**, 211-226 (2019).
82. H. D. Huh, D. H. Kim, H. S. Jeong, H. W. Park, Regulation of TEAD Transcription Factors in Cancer Biology. *Cells* **8** (2019).
83. K. W. Ter Horst, M. J. Serlie, Fructose Consumption, Lipogenesis, and Non-Alcoholic Fatty Liver Disease. *Nutrients* **9** (2017).
84. H. Itabe, T. Yamaguchi, S. Nimura, N. Sasabe, Perilipins: a diversity of intracellular lipid droplet proteins. *Lipids Health Dis* **16**, 83 (2017).
85. H. Wang *et al.*, Unique regulation of adipose triglyceride lipase (ATGL) by perilipin 5, a lipid droplet-associated protein. *J Biol Chem* **286**, 15707-15715 (2011).
86. L. L. Listenberger, A. G. Ostermeyer-Fay, E. B. Goldberg, W. J. Brown, D. A. Brown, Adipocyte differentiation-related protein reduces the lipid droplet association of adipose triglyceride lipase and slows triacylglycerol turnover. *J Lipid Res* **48**, 2751-2761 (2007).
87. R. C. Slieker *et al.*, Identification of biomarkers for glycaemic deterioration in type 2 diabetes. *Nat Commun* **14**, 2533 (2023).
88. Z. Si *et al.*, Identification of CYP46A1 as a new regulator of lipid metabolism through CRISPR-based whole-genome screening. *Faseb j* **34**, 13776-13791 (2020).
89. C. F. Cheng, H. C. Ku, H. Lin, PGC-1α as a Pivotal Factor in Lipid and Metabolic Regulation. *Int J Mol Sci* **19** (2018).
90. Q. Zhang *et al.*, MiR-30b-5p regulates the lipid metabolism by targeting PPARGC1A in Huh-7 cell line. *Lipids Health Dis* **19**, 76 (2020).

91. K. Fujisawa *et al.*, Modulation of anti-cancer drug sensitivity through the regulation of mitochondrial activity by adenylate kinase 4. *Journal of Experimental & Clinical Cancer Research* **35**, 48 (2016).
92. Y. Li *et al.*, CD36 favours fat sensing and transport to govern lipid metabolism. *Prog Lipid Res* **88**, 101193 (2022).
93. R. L. Silverstein, M. Febbraio, CD36, a scavenger receptor involved in immunity, metabolism, angiogenesis, and behavior. *Sci Signal* **2**, re3 (2009).
94. L. Marechal *et al.*, The CD36-PPARgamma Pathway in Metabolic Disorders. *Int J Mol Sci* **19** (2018).
95. A. Rodrigue-Way *et al.*, Scavenger receptor CD36 mediates inhibition of cholesterol synthesis via activation of the PPARγ/PGC-1α pathway and Insig1/2 expression in hepatocytes. *Faseb j* **28**, 1910-1923 (2014).
96. M. Rogne, K. Tasken, Compartmentalization of cAMP signaling in adipogenesis, lipogenesis, and lipolysis. *Horm Metab Res* **46**, 833-840 (2014).
97. W. Tang *et al.*, Adenylyl cyclase 1 as a major isoform to generate cAMP signaling for apoA-1-mediated cholesterol efflux pathway. *J Lipid Res* **59**, 635-645 (2018).
98. B. M. Stenson *et al.*, Liver X receptor (LXR) regulates human adipocyte lipolysis. *J Biol Chem* **286**, 370-379 (2011).
99. P. Arner, D. P. Andersson, J. Backdahl, I. Dahlman, M. Ryden, Weight Gain and Impaired Glucose Metabolism in Women Are Predicted by Inefficient Subcutaneous Fat Cell Lipolysis. *Cell Metab* **28**, 45-54 e43 (2018).
