# Supplementary material for: Heterogeneity of foam cell biogenesis across diseases
Source: bioRxiv. 2023 Jun 8:2023.06.08.542766. Preprint. [Version 1] doi: 10.1101/2023.06.08.542766 (PMC10274805; doi:10.1101/2023.06.08.542766)
Supplement: Supplementary file 1 [file media-1.pdf]

Supplementary Information

Heterogeneity of foam cell biogenesis across diseases

Valentina Guerrini[1*], Brendan Prideaux[2], Rehan Khan[1], Selvakumar Subbian[1], Yina Wang[3], Evita Sadimin[4*], Siddhi Pawar[3], Rahul Ukey[1], Eric A. Singer[4*], Chaoyang Xue[3], Maria Laura Gennaro[1**]

Supplementary Figures

Fig. S1. Low-magnification images of murine lungs uninfected and infected with *C. neoformans* H99.

Fig. S2. Hematoxylin-eosin staining and MALDI imaging of uninfected murine lung sections.

Fig. S3. Macrophage lipid droplet accumulation in response to infection with live *C. neoformans*, or treatment with cell-free fungal culture medium, or with heat-killed fungal cells.

Fig. S4. Upregulated metabolism-associated pathways in *M. tuberculosis*-infected human monocyte-derived macrophages.

Fig. S5. Effect of TNFα on lipid droplet accumulation in human monocyte-derived macrophages.

Fig. S6. Spatial distributions of cholesterol ester (CE) species in papillary renal cell carcinoma (pRCC).

Fig.S7. Spatial distribution of triglyceride (TAG) species in papillary renal cell carcinoma (pRCC).

Fig. S8. Production of cytokines by human monocyte-derived macrophages treated with ACHN-conditioned culture medium and role of IL-8 in lipid droplet accumulation.

Supplementary Tables

Table S1. List of triglyceride and cholesteryl ester species detected in tissues by MALDI-MSI.

Table S2a. Top five down- and up-regulated genes in macrophages infected with *M. tuberculosis.*

Table S2b. Associated genes related to top five down- and up-regulated genes in macrophages infected with *M. tuberculosis.*

Table S3a. Top five down- and up-regulated genes in macrophages infected with *C. neoformans.*

Table S3b. Associated genes related to top five down- and up-regulated genes in macrophages infected with *C. neoformans.*

Table S4. Levels of cytokines in supernatants of ACHN cell cultures and MDM treated with ACHN-conditioned culture medium.

Table S5a. Top five down- and up-regulated genes in macrophages treated with ACHN-conditioned medium.

Table S5b. Associated genes related to top five down- and up-regulated genes in macrophages treated with ACHN-conditioned culture medium.

Supplementary Materials and Methods

Supplementary Figures

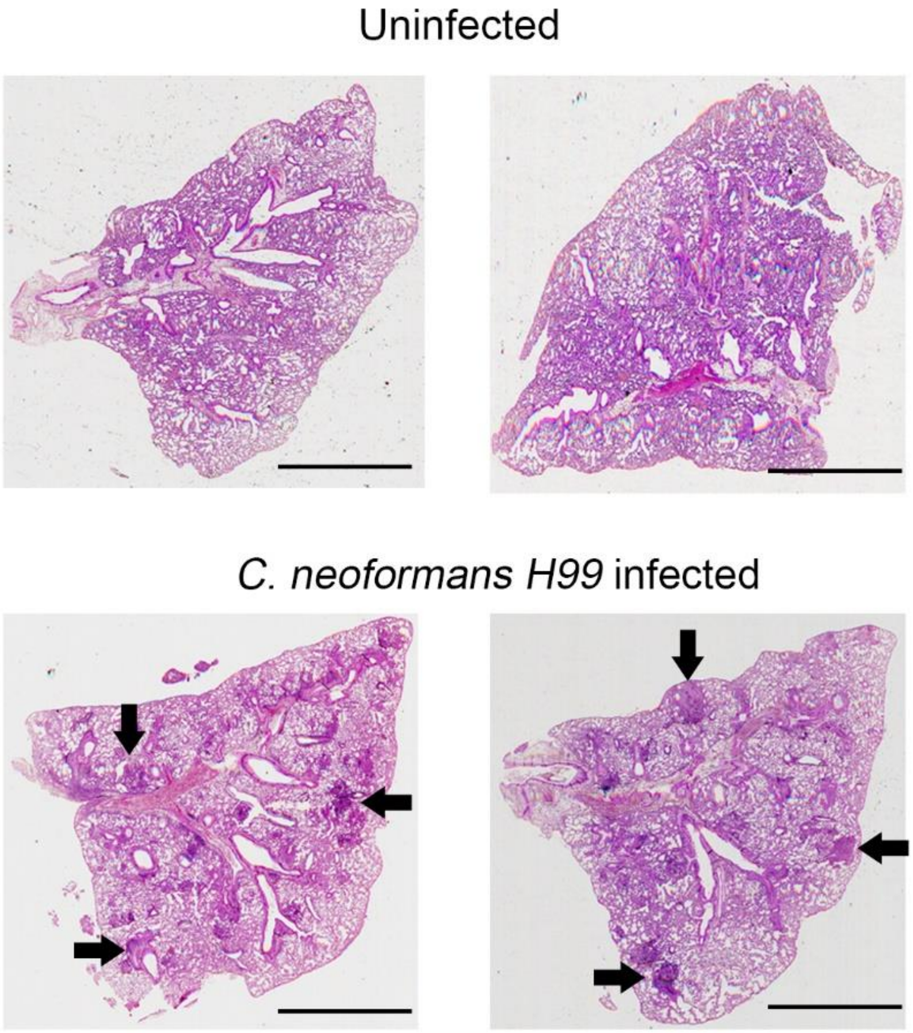

Supplementary Fig. S1. Low-magnification images of murine lungs uninfected and infected with *C. neoformans* H99. H&E staining of uninfected (top panels) and infected (bottom panels) lung sections. Black arrows show cellular aggregates in the infected tissue. Images were photographed at 20x magnification; scale bar is 2 mm.

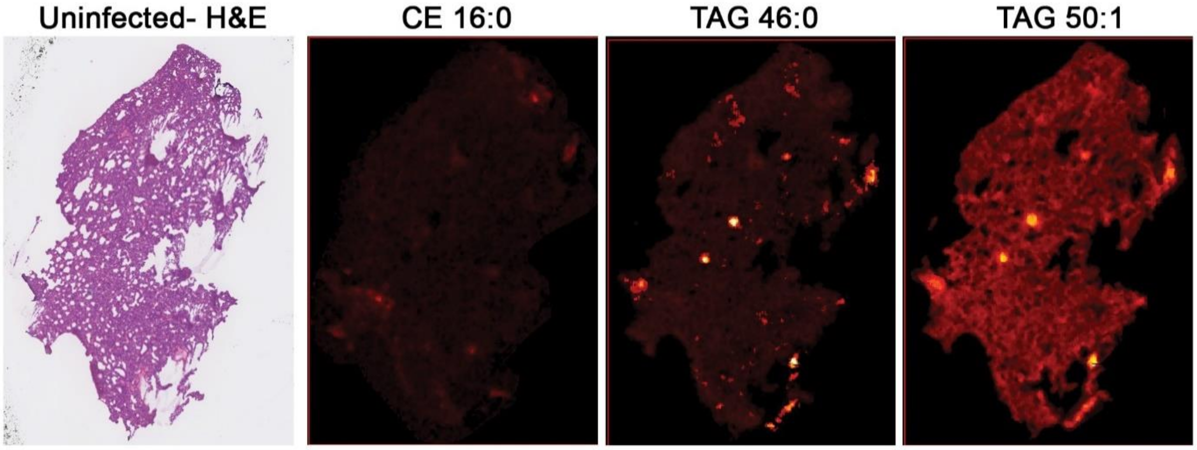

Supplementary Fig. S2. Hematoxylin-eosin staining and MALDI imaging of uninfected murine lung sections. The left panel shows H&E staining of uninfected lung sections. The three additional panels show MALDI imaging of storage lipids in lung sections contiguous to those used for H&E staining. Representative species are shown, CE (16:0) and TAG (46:0 and 50:1), corresponding to those shown in Fig. 1C (TAG and CE species detected are listed in **Table S1**).

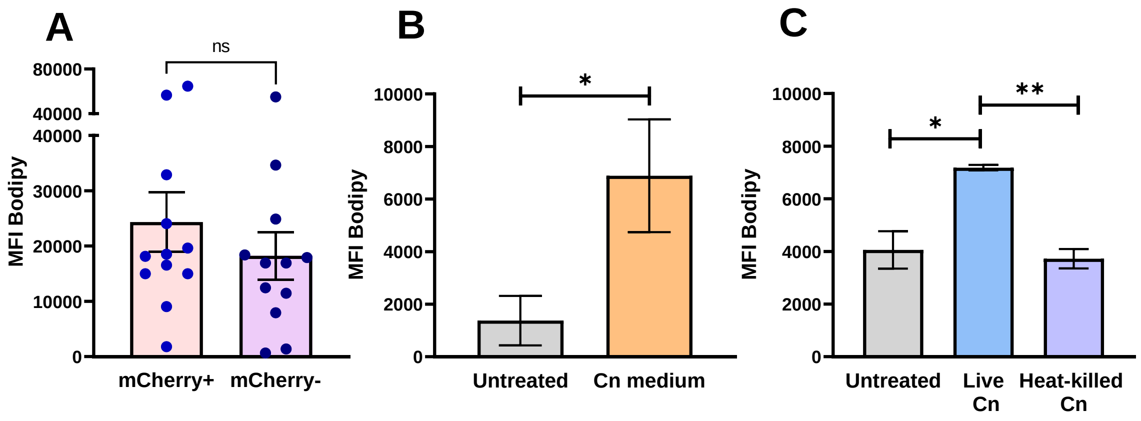

Supplementary Fig. S3. Macrophage lipid droplet accumulation in response to infection with live *C. neoformans*, or treatment with cell-free fungal culture medium, or with heat-killed fungal cells. In all panels, lipid droplet content of monocyte-derived macrophages (MDM) was measured by imaging flow cytometry and expressed as median fluorescence intensity (MFI) of Bodipy 493/503. **A.** MDM were infected with *C. neoformans* expressing mCherry and lipid droplet content was measured at 24 h post-infection in macrophages in the infected culture wells carrying or not carrying intracellular fungi (mCherry+ and mCherry-). Each dot represents one human donor. **B.** MDM were treated for 7 days with cell-free culture filtrate of *C. neoformans* grown in RPMI medium (Cn medium) or sterile RPMI medium (untreated). **C.** THP1 cells were treated with live or heat-killed *C. neoformans* (Cn) for 24 h. Statistics: mean and SEM (A-B) and mean and SD of triplicates (C-D) are shown; ns, non-significant; *, $p < 0.05$; **, $p < 0.01$ (paired *t*-test).

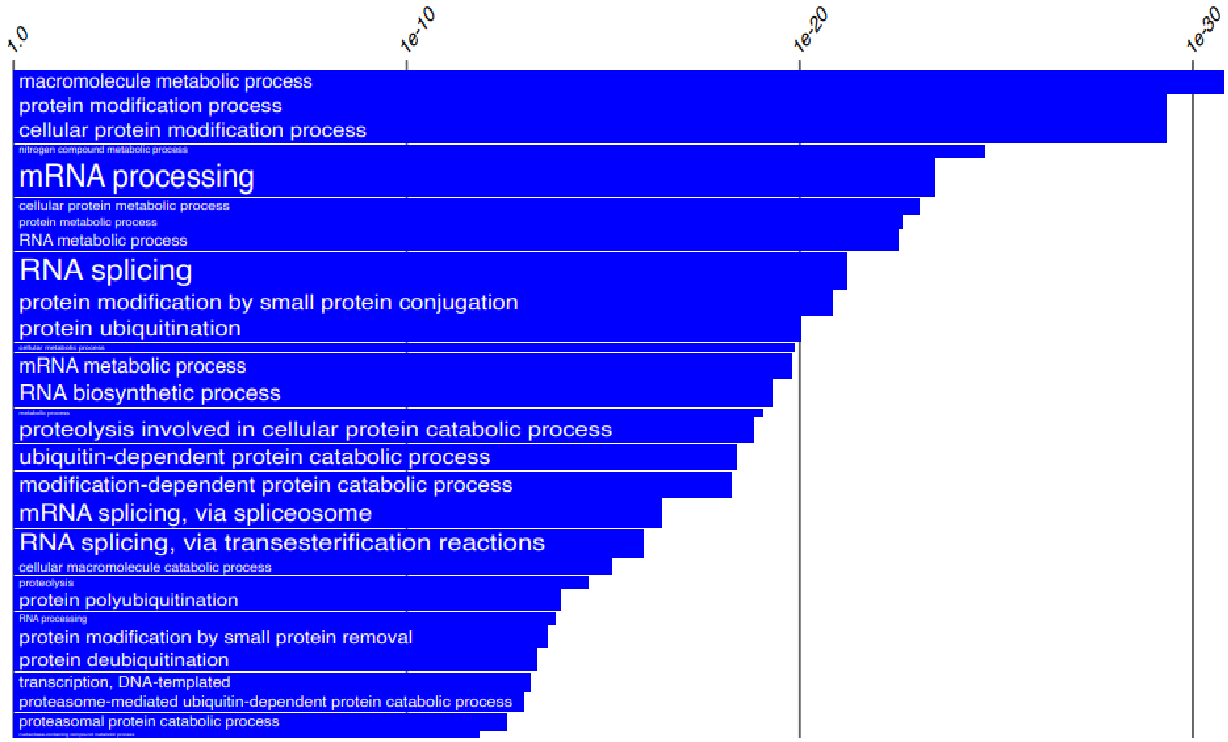

Supplementary Fig. S4. Upregulated metabolism-associated pathways in *M. tuberculosis*-infected human monocyte-derived macrophages. The figure shows Gene Ontology (GO) annotations related to metabolic processes that were upregulated in *M. tuberculosis*-infected MDM relative to uninfected control cells. The differential expression between sample classes (infected vs uninfected) was tested with coincident extreme ranks in numerical observations (CERNO). Pathways were selected using a cutoff false discovery rate of 0.05; the *p*-values for these pathways were plotted on the *x*-axis. To represent effect size, pathway gene sets containing fewer genes were given greater bar height/font size than were larger sets that yielded similar *p* values.

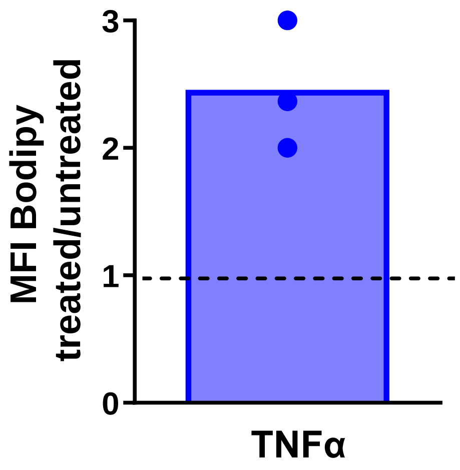

Supplementary Fig. S5. Effect of TNFα on lipid droplet accumulation in human monocyte-derived macrophages. MDM were stimulated with recombinant TNFα (2.5-40 ng/ml – different donors showed maximal effect at different doses within this range) and lipid droplet content was measured by imaging flow cytometry, as described in Fig. 2. Each dot represents a donor. *, p <0.05 (one sample t-test).

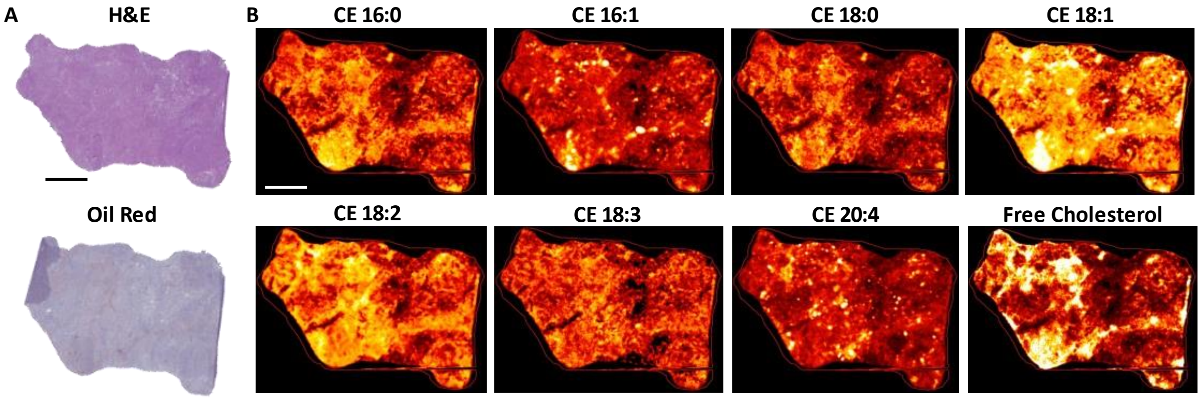

Supplementary Fig. S6. Spatial distribution of cholesterol ester (CE) species in papillary renal cell carcinoma (pRCC). A. Hematoxylin-eosin- and oil-red-stained kidney sections contiguous to those used for MALDI imaging. **B.** MALDI-2-MSI signal distribution for the major detected cholesterol ester species. Scale bar = 5 mm

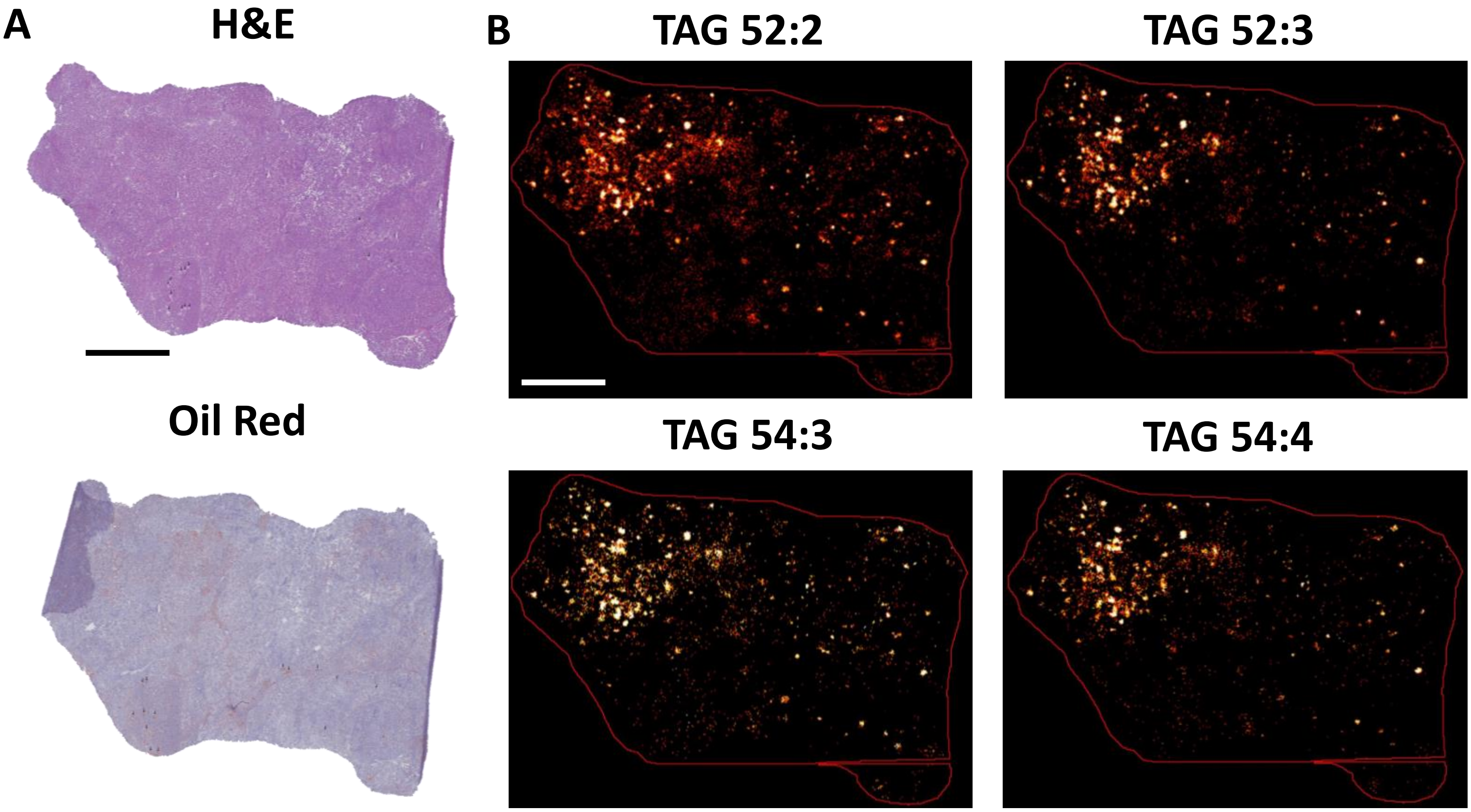

Supplementary Fig. S7. Spatial distribution of triglyceride (TAG) species in papillary renal cell carcinoma (pRCC). A. Hematoxylin-eosin- and oil-red-stained kidney sections contiguous to those used for MALDI imaging. **B.** MALDI-2-MSI signal distribution for four of the major detected TAG species. Scale bar = 5 mm

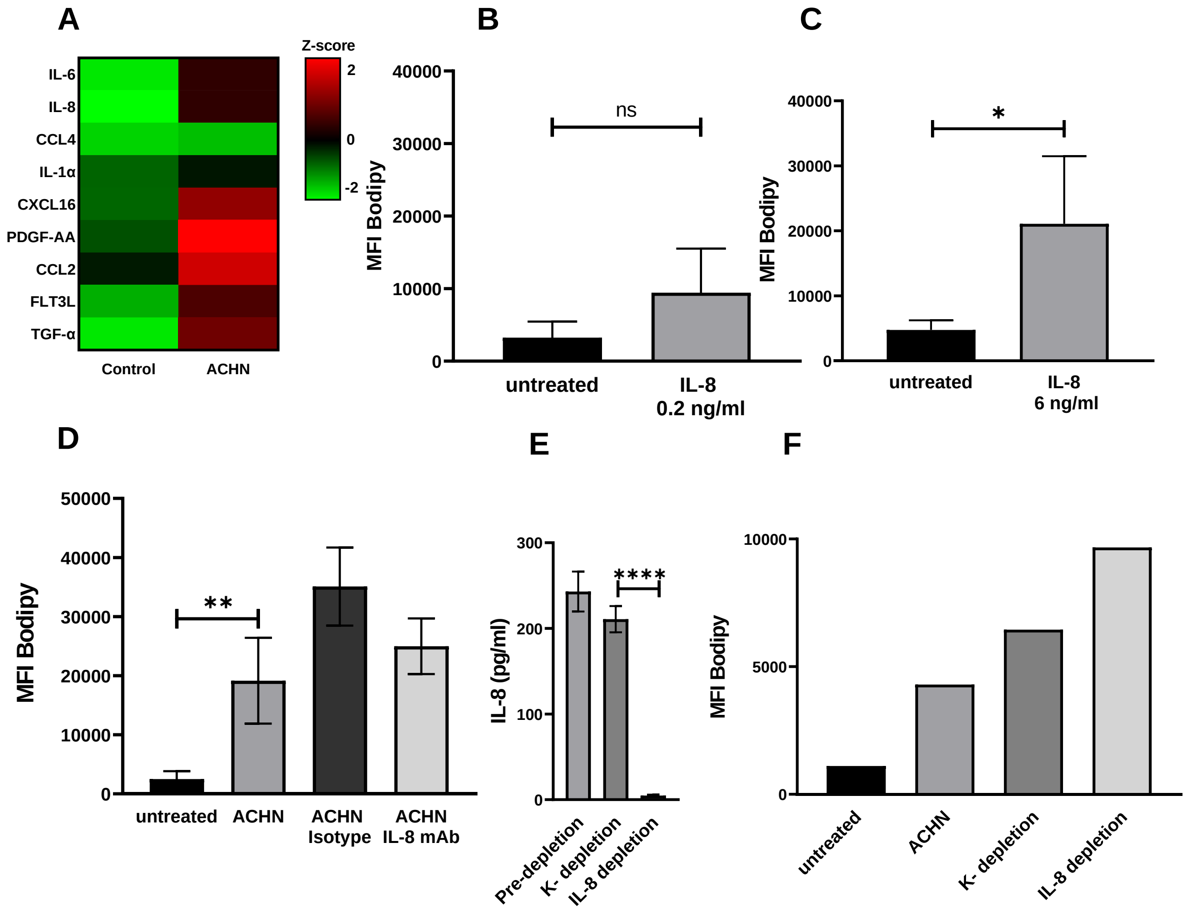

Supplementary Fig. S8. Production of cytokines by human monocyte-derived macrophages treated with ACHN-conditioned culture medium and role of IL-8 in lipid droplet accumulation. A. The heat map shows cytokines and chemokines differentially expressed by MDM exposed to conditioned medium from ACHN cell cultures relative to control, untreated MDM, as described in Fig. 4. Cytokines/chemokines were measured as technical duplicates using a 43-plex array and Luminex technology. The average *Z*-score for five donors was calculated for each analyte. Color gradient from green to red represents increasing concentrations. Data shown had $p < 0.05$ (paired *t*-test). **B-C.** Lipid droplet content of MDM was measured as median fluorescence intensity (MFI) of Bodipy 493/503 by imaging flow cytometry, following 7-day treatment with recombinant IL-8, at two different concentrations, as indicated. **D.** MDM were left untreated or treated for 7 days with ACHN-conditioned medium +/- anti-IL-8

neutralizing and isotype control antibodies (both at 1 μg/ml final concentration). Bodipy MFI was determined as in panels B-C. **E.** ACHN medium was depleted of IL-8 by incubation with magnetic beads conjugated with anti-IL-8 antibodies, and with negative control, unconjugated beads (K- depletion). IL-8 levels in the medium, pre-and post-depletion, were measured by ELISA. **F.** Lipid droplet content of MDM was measured (as in panels B-C) in MDM left untreated, treated for 7 days with conditioned ACHN medium left untreated (ACHN), mock depleted (K- depletion), or IL-8 depleted, as indicated. Data are shown as means of duplicate measurements. Statistics: mean and SD of triplicate measurements (B-D-E) and mean and SEM of multiple donors (C) are shown; ns, non-significant; *, p <0.05; **, p <0.01, ****, p <0.001 (B-D-E, unpaired *t*-test; C, Mann-Whitney test).

Supplementary Tables

Supplementary Table S1. List of triglyceride and cholesteryl ester species detected in tissues by MALDI-MSI.

TAG	Detected in *Cryptococcus*	Detected in pRCC	CE	Detected in *Cryptococcus*	Detected in pRCC
**Species**	**Tissue**	**Tissue**	**Species**	**Tissue**	**Tissue**
**48:00:00**	✓	✗	**16:00**	✓	✓
**48:01:00**	✓	✗	**16:01**	✓	✓
**48:02:00**	✓	✗	**18:00**	✓	✓
**48:03:00**	✓	✗	**18:01**	✓	✓
**50:00:00**	✓	✗	**18:02**	✓	✓
**50:01:00**	✓	✓	**18:03**	✓	✓
**50:02:00**	✓	✓	**20:01**	✓	✓
**50:03:00**	✓	✓	**20:02**	✗	✓
**50:04:00**	✓	✗	**20:04**	✓	✓
**52:01:00**	✓	✗			
**52:02:00**	✓	✓			
**52:03:00**	✓	✓			
**52:04:00**	✓	✓			
**52:05:00**	✓	✓			
**52:06:00**	✓	✗			
**54:02:00**	✓	✓			
**54:03:00**	✓	✓			
**54:04:00**	✓	✓			
**54:05:00**	✓	✓			
**54:06:00**	✓	✗			
**54:07:00**	✓	✗			
**56:03:00**	✓	✗			
**56:04:00**	✓	✗			

Triglyceride (TAG) species (identified by the total number of carbons and double bonds in the esterified fatty acids) and cholesteryl ester (CE) species detected by MALDI-MSI in the cryptococcus-infected lung tissues and papillary renal cell carcinoma (pRCC) tissue. ✓, detected; x, not detected.

Supplementary Table S2a. Top five down- and up-regulated genes in macrophages infected with *M. tuberculosis.*

Gene ID	Gene	log2FoldChange	*p* value
ENSG00000005469	CROT	-0.694245982	6.81E-10
ENSG00000183549	ACSM5	-1.875070163	2.15E-12
ENSG00000072210	ALDH3A2	-0.675169083	1.18E-15
ENSG00000131238	PPT1	-0.769679647	6.47E-16
ENSG00000168765	GSTM4	-0.846851963	4.64E-14
ENSG00000105325	FZR1	0.451795	1.01E-10
ENSG00000186187	ZNRF1	0.884946	4.79E-17
ENSG00000108306	FBXL20	0.849817	9.11E-17
ENSG00000186591	UBE2H	0.795385	3.10E-25
ENSG00000187555	USP7	0.206178	4.81E-06

Gene-level analyses were conducted by using as starting point the top five up- and down-regulated genes, which were ranked on the basis of log2 fold change (treated vs untreated) and number of occurrences in the pathways identified as having FDR < 0.05.

Supplementary Table S2b. Genes associated with the top five down- and up-regulated genes in macrophages infected with *M. tuberculosis*.

Gene ID	Gene	log2FoldChange	*p* value
ENSG00000215009	ACSM4	-1.65627	2.66E-05
ENSG00000130377	ACSBG2	-1.09841	3.99E-03
ENSG00000140284	SLC27A2	0.929524	2.40E-03
ENSG00000143554	SLC27A3	0.440446	2.69E-02
ENSG00000110090	CPT1A	-0.69747	6.60E-05
ENSG00000157184	CPT2	-0.32849	3.84E-05
ENSG00000154227	CERS3	1.207873	6.71E-03
ENSG00000139624	CERS5	0.437953	5.94E-06
ENSG00000177076	ACER2	-0.46598	2.77E-02
ENSG00000141510	TP53	-0.34037	1.19E-04
ENSG00000109670	FBXW7	0.514687	0.00000987
ENSG00000135679	MDM2	0.745793	1.91E-10
ENSG00000100219	XBP1	1.329735	1.36E-11
ENSG00000155792	DEPTOR	-2.04205	0.0000007
ENSG00000164181	ELOVL7	4.018986	1.04E-33
ENSG00000175445	LPL	-1.17544	6.57E-08
ENSG00000134184	GSTM1	-0.95459	0.000000069
ENSG00000213366	GSTM2	-1.23318	0.000000626
ENSG00000166851	PLK1	-1.07326	0.0025
ENSG00000092964	DPYSL2	-0.9294	1.38E-06
ENSG00000013588	GPRC5A	6.429513	2.92E-81
ENSG00000181773	GPR3	3.096538	1.07E-34
ENSG00000183484	GPR132	3.238435	2.91E-26
ENSG00000180758	GPR157	2.357273	5.3E-21
ENSG00000173698	GPR64	2.313555	8.38E-17
ENSG00000139572	GPR84	2.131323	4.78E-10
ENSG00000182885	GPR97	2.623284	1.95E-09
ENSG00000188316	ENO4	-1.32246	7.67E-07
ENSG00000077721	UBE2A	0.552901	1.40E-08
ENSG00000160087	UBE2J2	0.405355	2.54E-08
ENSG00000177889	UBE2N	0.348958	3.68E-08
ENSG00000131508	UBE2D2	0.331604	7.16E-07
ENSG00000185651	UBE2L3	0.381012	3.78E-06
ENSG00000160714	UBE2Q1	0.35768	2.01E-05
ENSG00000072401	UBE2D1	0.867935	3.27E-05

ENSG00000104343	UBE2W	0.503851	4.69E-05
ENSG00000182247	UBE2E2	0.746942	0.000065
ENSG00000078140	UBE2K	0.277765	0.000104
ENSG00000170142	UBE2E1	0.523719	0.000327
ENSG00000156587	UBE2L6	1.059927	0.000437
ENSG00000175931	UBE2O	0.349526	0.000465
ENSG00000130725	UBE2M	0.31568	0.000899
ENSG00000107341	UBE2R2	0.236068	0.002875
ENSG00000184182	UBE2F	0.245525	0.021078
ENSG00000198833	UBE2J1	0.305753	0.02493
ENSG00000187531	SIRT7	0.5201	1.33E-10
ENSG00000177463	NR2C2	0.771918	0.000254
ENSG00000182054	IDH2	-0.83851	2.92E-14
ENSG00000138413	IDH1	-0.94364	2.62E-18
ENSG00000142208	AKT1	-0.16012	0.045604
ENSG00000082701	GSK3B	0.515329	0.000158

Associated genes were differentially expressed genes ($p < 0.05$) found in the literature as functionally related to the top five up- and down-regulated genes in Table S2a.

Supplementary Table S3a. Top five down- and up-regulated genes in macrophages infected with *C. neoformans.*

**Gene**	**Gene Id**	**log2FoldChange**	***p* value**
ENSG00000116750	UCHL5	-0.20727	0.04472
ENSG00000138035	PNPT1	-0.54945	1.67E-10
ENSG00000130348	QRSL1	-0.37168	3.88E-07
ENSG00000196510	ANAPC7	-0.39341	0.000302
ENSG00000131467	PSME3	-0.30045	3.30E-05
ENSG00000109107	ALDOC	1.825971589	2.32E-10
ENSG00000111674	ENO2	1.489680497	9.70E-15
ENSG00000159399	HK2	1.121945578	6.74E-16
ENSG00000102144	PGK1	0.746990585	2.14E-08
ENSG00000111640	GAPDH	0.713009241	1.06E-07

Gene identification for gene-level analysis was performed as in Table S2a.

Supplementary Table S3b. Genes associated with the top five down- and up-regulated genes in macrophages infected with *C. neoformans.*

Gene	Gene Id	log2FoldChange	*p* value
ENSG00000185920	PTCH1	0.761480524	0.012629752
ENSG00000127528	KLF2	0.62022683	0.047195731
ENSG00000166819	PLIN1	0.973316711	0.003288899
ENSG00000147872	PLIN2	0.692199046	0.00059194
ENSG00000149925	ALDOA	0.568357915	4.63E-06
ENSG00000111669	TPI1	0.6108752	5.94E-07
ENSG00000171314	PGAM1	0.429844596	0.001449782
ENSG00000105220	GPI	0.684479515	7.26E-09
ENSG00000067057	PFKP	0.690877949	0.000281329
ENSG00000152256	PDK1	1.070412291	8.18E-08
ENSG00000067992	PDK3	0.363881857	0.004868894
ENSG00000134333	LDHA	1.050510273	3.90E-08
ENSG00000104812	GYS1	0.794960339	3.17E-07
ENSG00000174437	ATP2A2	-0.330898147	0.029025124
ENSG00000132356	PRKAA1	-0.186245418	0.045627492
ENSG00000131828	PDHA1	-0.222577362	0.025045384
ENSG00000072210	ALDH3A2	-0.331695382	7.63E-05
ENSG00000161618	ALDH16A1	-0.281990927	0.008872148
ENSG00000184254	ALDH1A3	-0.998071894	0.011142259
ENSG00000159423	ALDH4A1	-0.573208906	0.013428796
ENSG00000137124	ALDH1B1	-0.500558706	0.026687482
ENSG00000135218	CD36	-0.435604275	0.045176474

Associated genes were differentially expressed genes ($p < 0.05$) found in the literature as functionally related to the top five up- and down-regulated genes in Table S3a.

Supplementary Table S4. Levels of cytokines in supernatants of ACHN cell cultures and MDM treated with ACHN-conditioned culture medium.

Cytokine	ACHN (pg/ml)	ACHN-medium treated MDM (pg/ml)
IL-6	174	82
IL-8	543	4889
CCL4	137	2414
IL-1α	4	34
CXCL16	23	1898
PDGF-AA	46	37
CCL2	405	13775
FLT3L	10	22
TGF-α	6	17

The level of cytokines produced by ACHN cell cultures was calculated as average of technical duplicates for each analyte. MDM from donors (n=5) was treated with ACHN-conditioned culture medium. The average level in MDM supernatants from the five donors was calculated for each analyte.

Supplementary Table S5a. Top five down and upregulated genes in macrophages treated with ACHN-conditioned culture medium.

Gene	Gene Id	log2FoldChange	*p* value
ENSG00000109819	PPARGC1A	-1.108179567	0.0019129
ENSG00000179598	PLD6	-0.340258537	0.0313289
ENSG00000164742	ADCY1	-1.789945859	1.59E-05
ENSG00000156239	N6AMT1	-0.407562926	0.0036691
ENSG00000215009	ACSM4	-0.732880759	0.0301056
ENSG00000111674	ENO2	0.585748	0.005669
ENSG00000160883	HK3	0.417557	0.035561
ENSG00000111669	TPI1	0.280168	0.035629
ENSG00000102144	PGK1	0.263218	0.041578
ENSG00000162433	AK4	0.550335	0.00025

Gene identification for gene-level analysis was performed as in Table S2a.

Supplementary Table S5b. Genes associated with the top five down and upregulated genes in macrophages treated with ACHN conditioned culture medium.

Gene	Gene Id	log2FoldChange	*p* value
ENSG00000135218	CD36	-0.71398	0.002177
ENSG00000166411	IDH3A	-0.36846	0.012774
ENSG00000110090	CPT1A	-0.3713	0.048744
ENSG00000165092	ALDH1A1	-0.6716	0.005934
ENSG00000214456	PLIN5	0.763578	0.033995
ENSG00000007866	TEAD3	0.442895	0.043061
ENSG00000142583	SLC2A5	0.948899	0.001353
ENSG00000114268	PFKFB4	0.682204	3.73E-05

Associated genes were differentially expressed genes ($p < 0.05$) found in the literature as functionally related to the top five up- and down-regulated genes in Table S5a.

Supplementary Materials

Materials and Methods

Cell cultures. Peripheral blood mononuclear cells (PBMC) were isolated and monocyte-derived macrophages (MDM) were generated as previously described (1). Briefly, human blood was obtained from the New York Blood Center (Long Island City, NY, USA), and PBMC were isolated by Ficoll density gradient centrifugation (Ficoll-Paque, GE Healthcare, Uppsala, Sweden). Isolated PBMC were washed and resuspended in serum-free RPMI-1640 medium (Corning, Manassas, VA, USA) supplemented with 4 mM L-glutamine (Corning, Manassas, VA, USA) and seeded at a density of 1×10^7 cells/ml in tissue culture multiwell plates or flasks. After 4 hr, non-adherent cells were removed by washing five times with 1× PBS (Corning, Manassas, VA, USA) and the adherent fraction was cultured over 7 days in complete RPMI medium (RPMI-1640 supplemented with 10% fetal bovine serum (Seradigm, Radnor, PA, USA) and 4 mM L-glutamine.

The ACHN, A549, HeLa, Caco-2, THP-1, Raji cell lines were obtained from the American Type Culture Collection (ATCC). To obtain conditioned medium, cells were grown in complete RPMI for 6-9 passages, and cell culture media collected 24 hr after the last passage. The conditioned medium was filtered through a 0.4 μM filter to remove any cell debris and stored in aliquots at -80°C. All cell cultures were incubated in humidified atmospheric air containing 5% CO_2 at 37°C.

IL-8 depletion of ACHN conditioned medium. To deplete IL-8 from ACHN cell culture medium, 1.5 mg of streptavidin-conjugated Dynabeads M-280 (Invitrogen, Waltham, MA, USA) were incubated with 15 μg of a biotinylated antibody against IL-8 (R&D Systems, Minneapolis, MN, USA) for 30 min at RT in rotation. Beads were washed with 1× PBS for 5 times by magnetic sedimentation and incubated with 30 ml of ACHN culture medium in rotation for 30 min at RT. After removal of the beads by magnetic sedimentation and subsequent 0.4 μM filtration, IL-8-depleted ACHN was used for monocyte/macrophage treatments. ACHN medium incubated with unconjugated Dynabeads M-280 was used to treat monocyte/macrophage as negative control. IL-8 depletion was confirmed by using an ELISA assay kit following manufacturer's instructions (Invitrogen, Waltham, MA, USA).

Pathogens. mCherry-expressing *M. tuberculosis* $H_{37}Rv$ strain was grown in liquid medium to mid-log phase and frozen stocks prepared for macrophage infection as described (5). mCherry-

expressing *C. neoformans* H99 strain was grown in YPD medium overnight at 30°C with constant agitation, washed three times with 1× PBS, and resuspended in 1× PBS before use in macrophage infection. The final fungal concentration was adjusted to 1 x 10^8 cell/ml with complete RPMI medium [RPMI-1640 supplemented with 10% heat-inactivated fetal bovine serum (Seradigm, Radnor, PA, USA) and 4 mM L-glutamine]. A suspension of heat-killed *C. neoformans* was prepared by resuspending the fungal cells in 1× PBS and incubating the suspension at 75°C for 1 hr in a heat block. To prepare cell-free culture supernatant, *C. neoformans* H99 was grown in complete RPMI medium for 24 hr (end of logarithmic growth phase), filtered through a 0.2 μM filter, and used for macrophage treatments.

Macrophage treatments. For infection experiments, following 7 days of MDM differentiation, MDM were counted and infected with either *M. tuberculosis or C. neoformans*. The pathogen inoculum for infection was prepared by adding the microorganisms to supplemented RPMI-1640 medium (as above) to obtain an MOI of 4 colony-forming units (cfu) per cell. Pathogen clumps were disrupted by vortexing with sterile 3-mm-diameter glass beads for 2 min; this suspension was used for MDM infection. MDM were incubated with *M. tuberculosis* for 24h. MDM were infected with *C. neoformans* for 3 h, washed with 1× PBS three times, and incubated with fresh medium for 24 hr. When chemical inhibitors were tested, they were added to the MDM at the time of infection. For treatment of uninfected MDMs with recombinant TNFα, the cytokine was incubated with the MDMs for 24 hr.

At 24 hr post-infection, supernatants were collected and frozen for cytokine analysis and MDM were washed with 1× PBS three times and collected for subsequent analysis.
For treatment with cell line conditioned medium or IL-8 recombinant cytokine (Abcam, Cambridge, England), after PBMC isolation, the adherent fraction of PBMC was treated with conditioned media (50% fresh medium and 50% conditioned medium) or IL-8 for 7 days. Medium was replaced at 3 and 6 days of incubation. When neutralizing antibody or relative isotype controls were used, antibodies were added to the cells at the same time as the conditioned medium. At day 7 post-treatment, supernatants were collected and frozen for cytokine analysis, and MDM were washed with 1× PBS three times and collected for subsequent analysis.
Antibody/cytokine/chemical inhibitor doses were selected based on available EC_{50} data and toxicity profiles with untreated macrophages (Trypan Blue staining or MTS assay; CellTiter 96 Aqueous One Solution Cell Proliferation Assay Promega, Madison, WI, USA). Only

inhibitor/antibody doses resulting in >90% cell viability were utilized. The following concentrations were used: 30 nM A922500 (DGAT inhibitor) (Santa Cruz Biotechnology, Dallas, TX, USA), 0.4 nM rapamycin (mTORC1 inhibitor) (Selleckchem, Houston, TX, USA), 0.2-6 ng/ml recombinant IL-8 (Research and Diagnostic Systems, Minneapolis, MN, USA) and 2.5-40 ng/ml recombinant TNFα (Abcam, Cambridge, England), 1 μg/ml IL-8 neutralizing antibodies (MAB208; clone #6217) and mouse IgG1 isotype control (MAB002; clone#11711) (Research and Diagnostic Systems, Minneapolis, MN, USA).

Imaging flow cytometry. Imaging flow cytometry of MDM was performed as previously described (1). MDM were detached from tissue culture plates by incubating with 5 mM EDTA in 1× PBS pH 8 for 30 min followed by gentle scraping, washed once with 1× PBS, and fixed with 4% paraformaldehyde in 1× PBS for 45 min at room temperature. Cells were then washed with 1× PBS containing 0.1% bovine serum albumin (PBS-BSA), resuspended in 50 μl of PBS-BSA containing 5 μl of Fc receptor blocking solution, FcX (BioLegend, San Diego, CA), and incubated at room temperature for 5 min. After incubation, 50 μl of PBS-BSA containing 2.5 μl of CD11c APC antibody (clone S-HCL-3; BD Biosciences) were added to each tube, and samples were incubated at 4°C for 30 min. After washing with PBS-BSA, cells were stained with 0.3 μg/ml Bodipy 493/503 (Life Technologies, Carlsbad, CA) in 1× PBS for 15 min. For each experimental condition, data from 5,000–10,000 CD11c+ cells were acquired with an ImageStream^{X}Mark II imaging flow cytometer (Amnis Corporation, Seattle, WA) using 60× magnification. Image data were analyzed by IDEAS software version 6.0 (Amnis Corporation, Seattle, WA) after applying a compensation matrix and selecting the region of interest (lipid droplets) with the Spot Mask tool. Median fluorescence intensity and spots per cell were extracted.

Lipid quantification in macrophages. MDM were detached from the cell culture plates as described above and transferred to microcentrifuge tubes. Cell pellets were obtained by culture centrifugation at 300 *xg* and frozen for subsequent lipid measurement. Triglyceride-Glo™ Assay and Cholesterol/Cholesterol Ester-Glo™ Assay kits (Promega; Madison, WI, USA) were used to quantify triglycerides and free and total cholesterol, following the manufacturer's instructions.

Mouse infection and tissue collection. Female mice with an average weight of 20-25 g were used. C57BL/6 mice were purchased from the Jackson Laboratories. Animal studies were performed at the Public Health Research Institute Animal facility. All studies were conducted

following biosafety level 2 (BSL-2) protocols and procedures approved by the Institutional Animal Care and Use Committee (IACUC) and Institutional Biosafety Committee of Rutgers University under protocol 999901066. *C. neoformans* wild type strain H99 was cultured on YPD medium. To prepare fungal cells for infection, an overnight culture of *C. neoformans* H99 was washed three times with 1× PBS and the concentration of yeast cells was determined by hemocytometer counting. The final fungal concentration was adjusted with PBS to 2 x 10^6 cell/ml. Each mouse was infected intranasally with 1 x 10^5 H99 cells in a 50 µl volume after being anesthetized with a mix of Ketamine (12.5 mg/mL) and Xylazine (1 mg/mL). After infection, animals were weighed daily and monitored twice daily for progression of disease, including weight loss, gait changes, labored breathing, and fur ruffling. Infected animals were sacrificed at day 7 post-infection, according to the experimental design and the Rutgers University IACUC approved animal protocol. A part of the dissected lung tissues was fixed in 10% formalin solution and sent to the Rutgers histopathological core facility for section preparation and staining with hematoxylin and eosin (H&E) using standard protocols. Another portion of the lung tissues was placed on labeled plastic cryo histology trays and frozen on dry ice for 2 minutes in a Styrofoam container. The samples were then wrapped with aluminum foil and stored in labeled Ziploc bags at -80°C until use for MALDI imaging.

Human Biopsies. Papillary renal cell carcinoma tissue was obtained from the Rutgers CINJ Biorepository. Tissue collected during surgery was frozen by the Biorepository using the PrestoCHILL machine, which allows for ultrafast freezing. The tissue analyzed derived from a radical nephrectomy; the RWJUH surgical pathology report indicated that the tumor was Type 1 and WHO histologic grade G2.

Matrix-assisted laser desorption/ionization (MALDI) mass spectrometry. Frozen lung samples were used to prepare 12 µm-thick cryosections using a Leica CM1860 cryostat. Tissue sections were mounted onto indium tin oxide coated glass slides (Delta Technologies Limited, Loveland, Colorado) and stored individually in Ziploc bags at -80°C. Adjacent sections were prepared on Superfrost Plus™ glass microscope slides (Fisherbrand) for H&E staining, which was performed using standard protocols at the UTMB Histology core. Sections for MALDI imaging were coated with 2′,4′,6′-Trihydroxyacetophenone monohydrate at 10 mg/ml in 50:50 cyclohexane/methanol using a HTX TM Sprayer. 20 passes were performed over each tissue at a spray volume of 50 ml/min and nozzle temperature of 50°C. Once sprayed, samples were placed individually in Ziploc bags and immediately transferred to a -80°C freezer for storage.

Following MALDI-MS image acquisition, matrix was removed from the slides by serial rinses in 50% ethanol, and tissue sections were allowed to airdry at room temperature for 30 min. The tissue sections were then transferred to the UTMB histology core for H&E and Oil Red staining using standard protocols.

MALDI MS imaging was performed using a Q-Exactive HF mass spectrometer (Thermo Scientific, Bremen, DE) fitted with a MALDI/ESI Injector (Spectroglyph LLC, Kennewick, WA). Laser post-ionization (MALDI-2) was used to enhance analytical sensitivity for triglycerides and cholesteryl esters. Images were acquired at 20 micrometer voxel size, using a pulse energy of ~6 mJ and repetition rate of 30Hz. Q Exactive HF MS Scan parameters were optimized for triglyceride and cholesteryl ester detection: polarity – positive, scan range – 350-1500 m/z, resolution – 120,000, automatic gain control - off, maximum inject time 250 ms**.** ImageInsight™ (Spectroglyph LLC) software was used for initial data visualization and to convert data files into imzML format for visualization and further processing in SCiLS™ software (Bruker, Billerica, MA). TAG and CE species were predominantly detected as potassium adducts and lipid identifications were assigned within a 3pm mass tolerance range using Lipid Maps database. All lipid images produced were normalized to the total ion chromatogram.

Cytokine measurement. A panel of cytokines/chemokines/growth factors was measured using a 43-Plex Luminex technology at the Rutgers Immune Monitoring and Advanced Genomics Core Facility (TNF-α, IL-6, PDGF-BB, IL-3, IL-8, CXCL10/IP-10, IL-10, EGF, IL-1β, IFN-ɣ, IL-1ra/IL-1F3, IL-12 p70, CCL3, CCL22/MDC, CCL4/MIP-1β, IL-1α/IL-1F1, CXCL16, IL-17/IL-17a, IL-2, TNF-β, GM-CSF, FGFbasic, M-CSF, CXCL9, IL-5, G-CSF, PDGF-AA, CCL2/JE/MCP-1, IFN-α, IL-13, Chemerin, CD40L, FLT3L, IL-18, IL-27, VEGF, IL-7, IL-17E/IL-25, CCL8/MCP-2, IL-4, IL-15, TGF-α, IL-12/IL-23 P40. Assays were performed in microtiter plates and plates were read with the Luminex 200 instrument. Each sample was measured in duplicate.

Transcriptomics and pathway analysis.

RNA was isolated by using TRI reagent (Molecular Research Center, Cincinnati, OH, USA) according to manufacturer's instructions. RNA-Seq was conducted at the Rutgers Genomics Center. The quality of RNA was first assessed for integrity on an Agilent TapeStation using high sensitivity RNA kit, PN-5067-5579 (Agilent technologies Inc, CA). Samples with RNA integrity number (RIN) >7.0 were considered to have sufficient quality for subsequent processing. Illumina compatible cDNA libraries generated for polyA selected mRNA using NEB next ultra RNA library preparation kit Cat#E7530L (New England Biolabs Inc, MA). The cDNA libraries

were purified using AmpureXP beads, Product No: A63882 (Beckman Coulter) and analyzed on an Agilent TapeStation using HS D1000 screen tape PN- 5067-5584 (Agilent Technologies Inc, CA) to estimate the size of the library and quantitated using Qubit 4 Fluorometer, using HS reagent kit, Cat. No. Q33231 (Thermofisher Scientific, MA). Equimolar amounts of barcoded libraries pooled together and sequenced on Illumina NovaSeq 6000 Instrument (Illumina, San Diego, CA) using SP flow cells kits (cat # 20040326) with 2x150 cycles configuration.

Read count and gene-level transcript abundance analysis were performed by the Rutgers Molecular and Genomics Informatics Core. In brief, raw transcriptome reads were assessed for quality control (FASTQC v0.11.8) and trimmed for quality and adapter contaminant (cutadapt v 2.5). Trimmed reads were aligned to the mouse genome (GRCh37) using STAR (v2.6.1), followed by transcript abundance calculation and hit count extraction with StringTie (v2.0) and featureCounts (v1.6.4), respectively. Differential expression was calculated by paired-sample T-tests.

The differential expression between sample classes (treated vs non treated for each condition) was tested with coincident extreme ranks in numerical observations (CERNO) (2) for metabolism-related Gene Ontology gene sets. The Benjamini–Hochberg method was used to calculate the false discovery rate (FDR). Gene sets (pathways) were identified at a cutoff false discovery rate of 0.05. Gene-level analyses were conducted by using as starting point the top five up- and down-regulated genes, which were ranked on the basis of log2 fold change (treated vs untreated) and frequency in the identified gene sets. Associated genes were differentially expressed genes ($p < 0.05$) found in the literature as functionally related to the top five up- and down-regulated genes.

1. V. Guerrini *et al.*, Storage lipid studies in tuberculosis reveal that foam cell biogenesis is disease-specific. *PLoS Pathog* **14**, e1007223 (2018).
2. E. Croze, K. D. Yamaguchi, V. Knappertz, A. T. Reder, H. Salamon, Interferon-beta-1b-induced short- and long-term signatures of treatment activity in multiple sclerosis. *Pharmacogenomics J* 10.1038/tpj.2012.27 (2012).
